# Cellular Contact Guidance Emerges from Gap Avoidance

**DOI:** 10.1016/j.xcrp.2020.100055

**Published:** 2020-05-20

**Authors:** Antonetta B.C. Buskermolen, Tommaso Ristori, Dylan Mostert, Mark C. van Turnhout, Siamak S. Shishvan, Sandra Loerakker, Nicholas A. Kurniawan, Vikram S. Deshpande, Carlijn V.C. Bouten

**Affiliations:** 1 Department of Biomedical Engineering, Eindhoven University of Technology, Eindhoven, the Netherlands; 2 Institute for Complex Molecular Systems, Eindhoven University of Technology, Eindhoven, the Netherlands; 3 Department of Structural Engineering, University of Tabriz, Tabriz, Iran; 4 Department of Mechanical Engineering, University of Cambridge, Cambridge, UK

**Keywords:** cell alignment, microcontact printing, statistical mechanics, cell adhesion, stress fibers, substrate anisotropy, contact guidance, protein patterning, focal adhesion, cell organization

## Abstract

In the presence of anisotropic biochemical or topographical patterns, cells tend to align in the direction of these cues—a widely reported phenomenon known as “contact guidance.” To investigate the origins of contact guidance, here, we created substrates micropatterned with parallel lines of fibronectin with dimensions spanning multiple orders of magnitude. Quantitative morphometric analysis of our experimental data reveals two regimes of contact guidance governed by the length scale of the cues that cannot be explained by enforced alignment of focal adhesions. Adopting computational simulations of cell remodeling on inhomogeneous substrates based on a statistical mechanics framework for living cells, we show that contact guidance emerges from anisotropic cell shape fluctuation and “gap avoidance,” i.e., the energetic penalty of cell adhesions on non-adhesive gaps. Our findings therefore point to general biophysical mechanisms underlying cellular contact guidance, without the necessity of invoking specific molecular pathways.

## Highlights

Cells exhibit an optimum alignment (contact guidance) to substrate micropatterns
Contact guidance does not require constrained alignment of focal adhesions
Statistical mechanics model reveals the energetic role of non-adhesive gaps
The non-adhesive gap size determines the degree of cell alignment

## Graphical Abstract

**Figure:**
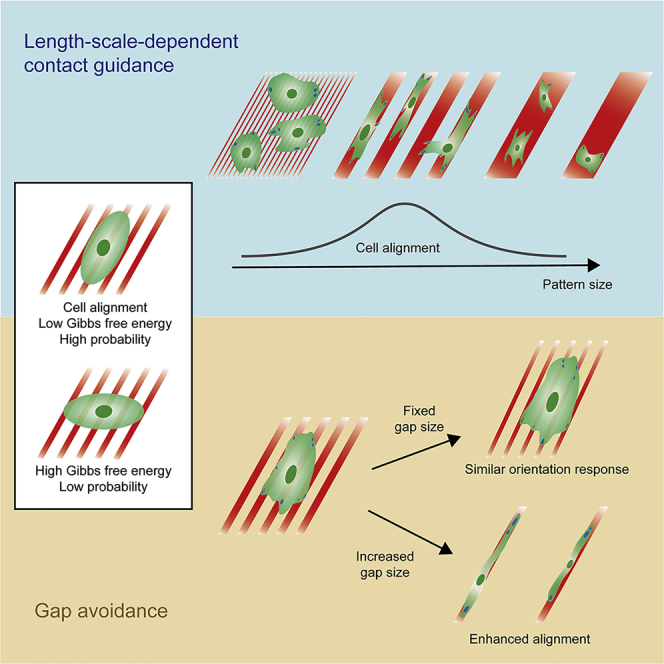

## Teaser

Buskermolen et al. report that cells align to substrate micropatterns in a highly length-scale-dependent manner. This alignment emerges from energetically favorable cell elongation and “gap avoidance”: minimization of cellular adhesions on non-adhesive gaps. This finding points to biophysically driven mechanisms underlying cellular contact guidance without necessarily invoking specific molecular pathways.

## Introduction

Cell organization plays a crucial role in the micro-architecture of tissues, dictating their biological and mechanical functioning.^1^ *In vivo*, cells are embedded in the extracellular matrix (ECM), which generally comprises a network of organized micrometer-scale fibers that provide cells with anisotropic geometrical cues.^2^, ^3^, ^4^ Cells typically align in the direction of the cues—a phenomenon known as cellular contact guidance. Contact guidance has been widely observed for several decades^5^^,^^6^ and has been shown to affect various downstream cell behaviors, including survival, motility, and differentiation.^7^, ^8^, ^9^ Uncovering the underlying mechanisms is critical for a better understanding of tissue and organ morphogenesis and regeneration.^10^

Numerous studies have demonstrated and examined contact guidance effects, typically by culturing cells on microfabricated anisotropic substrates consisting of microgrooves, adhesive lines, or fibers (see Tamiello et al.^11^ for an extensive review). Collectively, these studies have shown that contact guidance intriguingly occurs on substrate patterns of a wide range of sizes. For example, it was shown that contact guidance at length scales smaller than focal adhesions (FAs) arises as a result of constrained FA alignment and maturation in the direction of anisotropy.^9^^,^^12^ More recently, we showed that cells constrained on single lines of fibronectin align in the line direction not only when the line width is smaller than the cell body, thereby enforcing cell alignment, but also on wider lines, as a result of entropy-mediated mechanisms.^13^ However, contact guidance due to cues of intermediate-length scales (μm to hundreds of μm) has remained unexplored.

In the present study, we investigated the emergence of cellular contact guidance resulting from anisotropic cues at these intermediate length scales, from the typical size of FAs to the typical cell length. We found that myofibroblasts exhibited two regimes of cellular alignment: one at small length scales of the anisotropic cue, where cell alignment is induced by multiple patterns, and one at large-length scales, as a result of spatial confinement of the whole cells. Interestingly, these alignments occurred in the absence of spatially constrained FA alignment, suggesting that contact guidance can result from an alternative mechanism. To understand this, we extended a recently established statistical mechanics framework for living cells,^14^ enabling the simulation of cells on substrates with a heterogeneous distribution of ligands. Quantitative comparison between our experimental and computational data indicates that contact guidance arises from the minimization of cellular adhesions on non-adhesive regions and that non-adhesive gaps play a decisive role for cell alignment.

## Results

### Cellular Alignment and Intracellular Organization Are Controlled by Substrate Anisotropy at Micron- to Cell-Size Scale

**Figure 1:**
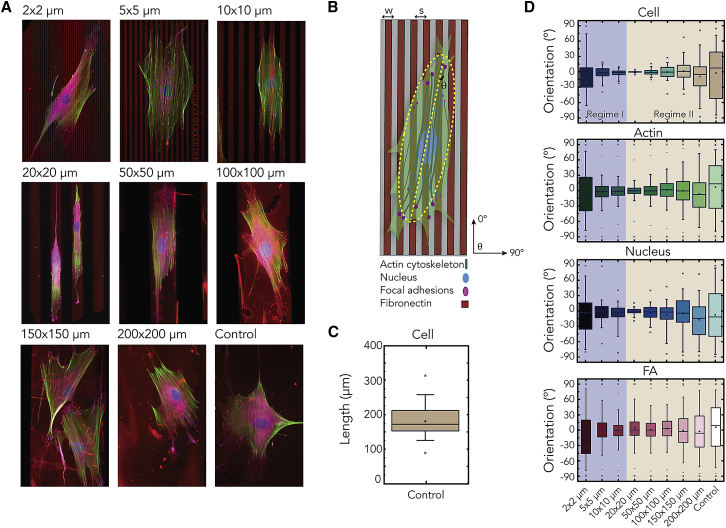
Two Regimes of Cellular Alignment (A) Representative immunofluorescence images of myofibroblasts on parallel lines (*w* × *s* μm) of fibronectin (red) stained for the FAs (magenta), actin cytoskeleton (green), and nucleus (blue). (B) Schematic diagram showing the analysis of cell orientation based on the best-fitted ellipse (dashed yellow). (C) The length of a cell on a homogeneous substrate (control). (D) The cell, actin fiber, nucleus, and FA orientation, where 0° represents the direction of the lines. The boxes of the boxplots represent the quartiles of the distributions, with the whiskers indicating the outliers in the experiments and the 5^th^ and 95^th^ percentiles of the distributions. Note that, with this data representation, the median is at 0° and the box ranges from –45° to 45° when the distribution of cell orientation is perfectly isotropic. The data reported are results from three independent samples; at least 60 cells were considered per condition.

To mimic the fibrillar nature of the ECM and examine the effects of substrate anisotropy on cellular alignment, we created micropatterns consisting of parallel lines of fibronectin with defined widths and inter-line spacings using microcontact printing and seeded human myofibroblasts on these microcontact printed substrates. This experimental approach allowed us to systematically probe cellular contact-guidance response in a purely planar setup, without the need to consider the influence of the third dimension as in studies employing microfabricated grooves and ridges. The line width *w* and inter-line spacing *s* ranged from 2 to 200 μm and were initially chosen to be equal (i.e., *w* = *s*) to maintain a constant cell-substrate contact area. The lower limit of *w* = 2 μm was motivated by the size of typical mature FAs,^15^^,^^16^ whereas the upper limit of *w* = 200 μm is large enough to allow cell spreading on one single line (cf. individual myofibroblast length on fibronectin-coated substrate; Figure 1C).

Images obtained 24 h after seeding showed that cell morphology and orientation are strongly influenced by the width of the lines (Figure 1A). On the thinnest lines (*w* = 2 μm), there was only a weak cell-alignment effect due to the micropatterned lines. When *w* was increased up to 20 μm, cells increasingly elongated and aligned parallel to the lines. The trend inverted when *w* was further increased up to 200 μm. We quantified the changes in cell shape and orientation for more than 600 cells on the substrates using an automated morphometric analysis of the immunofluorescence images.^17^ Briefly, we fitted an ellipse to the cell outline and defined the orientation angle θ as the angle between the major axis of the best-fitted ellipse and direction of the lines (Figure 1B). The analysis revealed that, with increasing *w*, the distribution of θ around 0° strongly narrowed up to *w* = 20 μm, indicating enhanced cell alignment, and then broadened back toward an isotropic distribution, resembling that on homogeneous substrates (control; Figure 1D). Furthermore, we noted that *w* = 20 μm also demarcates the transition from the situation where cells adhered on more than one line to the situation where cells fitted within single lines.

These data demonstrate that the cellular orientation response can be divided into two regimes: regime I for w< 20 μm, where cell alignment was induced by multiple lines, and regime II for *w* ≥ 20 μm, where cell alignment was influenced by the spatial confinement within single lines. In regime II, as we previously observed,^13^ cell alignment decreased with increasing *w*. In regime I, cell alignment decreased with decreasing *w*. The two regimes were also captured by the order parameter Θ (see Equation 1 in the Experimental Procedures and Figure S1), clearly showing that the order transitions at *w* = 20 μm.

Cell alignment on the micropatterned lines was accompanied by changes in cell morphology, as quantified through aspect ratio and adhesion area (Figure S2A). The largest degree of alignment was observed when cells were most elongated. Because cell morphology is governed by the dynamics and properties of intracellular structural components, such as the nucleus and individual actin fibers,^18^ we hypothesized that the two observed regimes should also reflect in the organization of these components. Indeed, the actin fibers and nuclei showed similar trends as for the cell orientations, including the two regimes of alignment (Figures 1D and S2B). This confirms that micrometer-scale variations in the extracellular patterns can tune cell orientation as well as intracellular organization.

### Contact Guidance Does Not Require Constrained Alignment of FAs

On patterns with dimensions smaller than FAs, it has been previously reported that spatially constrained alignment of FAs play a central role in contact guidance.^9^ Generally, when cells adhere to substrates homogeneously coated with fibronectin, they first form small, nascent FAs (0–2 μm long), which can either disappear or develop into 2- to 6-μm-long, mature FAs.^19^ Because a minimum length of 2 μm has been shown to be required for local contacts to establish adhesions^20^ and the FAs of our cells can grow to lengths much larger than 2 μm (Figure S3), we reasoned that the lines with *w* = 2 μm would provide an area for FA maturation and orientation only in the direction of the lines and that FAs could have a wide distribution of orientations on 5- and 10-μm lines, where FA maturation is not spatially constrained. By examining the FA and cell orientation on these line widths, we therefore can test the requirement of FA alignment for cellular contact guidance. Indeed, Ray et al.^9^ have recently suggested that relaxing spatial constraints on FA maturation and consequently FA organization diminishes cellular alignment. Surprisingly, we obtained the opposite result: on lines of *w* = 2 μm, a wide distribution of orientation angles of FAs was observed, whereas the FAs became slightly more aligned at larger line width *w* (Figure 1D). This trend is similar to, but weaker than, the orientation response of the cells. Therefore, our data show that, at length scales larger than FA size, increasing the adhesive area for FAs leads to the counterintuitive increase of FA and cell alignment in the direction of the lines. This suggests that contact guidance at these length scales does not arise from spatially constrained alignment of FAs, which is an underlying mechanism of contact guidance at smaller scales.^9^^,^^12^

**Figure 2:**
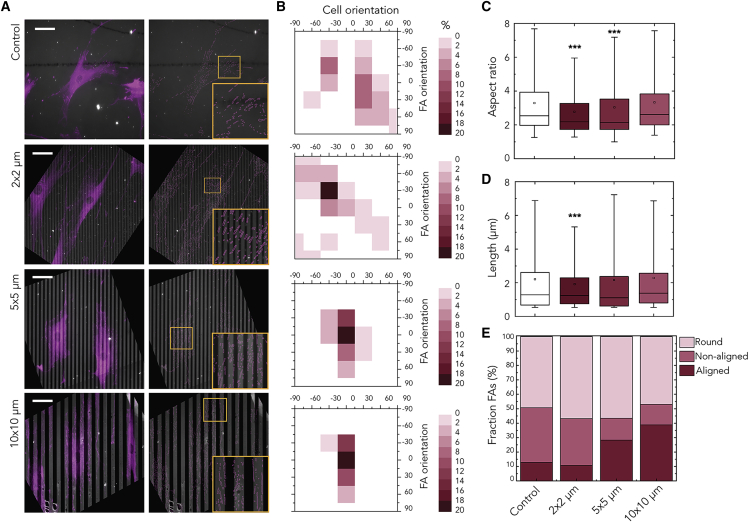
Constrained FAs Do Not Guide Cellular Alignment (A) Representative immunofluorescence images of the FAs (magenta) of myofibroblasts on parallel lines (*w* × *s* μm) of fibronectin (gray). The detected outlines are shown in magenta, and the orange rectangles marked areas show zoom-in images of the FAs. Scale bars: 50 μm. (B) 2D density plots for each line width showing the correlation between cell and FA orientation, where 0° represents the direction of the lines. The color scales show the percentage of FAs in a specific orientation with more intense pink corresponding to more FAs in this direction. These plots indicate that cellular alignment was not fully governed by constrained FAs. (C–E) Quantitative analysis of the aspect ratio (C), length of long axis (D), and percentage of FAs that are round, non-aligned, or aligned (E). The boxes of the boxplots in (C) and (D) represent the quartiles of the distributions, with the whiskers indicating the outliers in the experiments and the 5^th^ and 95^th^ percentiles of the distributions. The data reported are results from three independent samples, and at least 20 cells were considered per condition. ∗∗∗p < 0.001 with respect to control.

To further confirm this observation, we investigated in more detail the morphology and organization of FAs in regime I. The analysis showed that lines of *w* = 2 μm were able to constrain FA maturation. In particular, vinculin staining showed that FAs were primarily formed on the adhesive fibronectin lines and that the distributions of the FAs were markedly influenced by the lines (Figure 2A). Specifically, for *w* = 5 and 10 μm, we observed rows of FAs lining up along the edge of the lines (Figure 2A, insets). For cells on the 2-μm-wide lines, we found small, non-aligned adhesions, in contrast to the large, non-aligned adhesions on homogeneously coated substrates of fibronectin. Moreover, FAs were significantly smaller for 2-μm lines relative to the homogeneous substrate (Figure S2C), consistent with the notion that lines of 2-μm width provided a geometrical constrain for FA maturation. Increasing the line width *w* resulted in more aligned, elongated FAs in the direction of the lines (Figure 2A). We further characterized the size and shape of individual FAs by determining their length and aspect ratio, respectively. The length and aspect ratio of FAs on 2-μm lines were significantly smaller compared to those on homogeneous substrates, whereas the long axes of FAs on 5- and 10-μm lines were equal to those on the homogeneous substrates (Figures 2C and 2D). The aspect ratio of FAs on 5-μm lines was smaller compared to FAs on 10-μm lines, meaning that FAs on 5-μm lines are long and wide, although FAs on 10-μm lines are thinner.

Given these differences in the FA morphology, we classified the FAs into three categories: “round” FAs are those with aspect ratio smaller than 1.6; “non-aligned” FAs are non-round FAs whose orientation angle θ is larger than 20°; and “aligned” FAs are non-round FAs with θ smaller than 20°.^17^ We found that the fraction of aligned FAs increases and the fraction of non-aligned FAs decreases for increasing line widths (Figure 2E). Interestingly, for 2-μm lines, only a small amount of FAs aligned in direction of the lines (11%), comparable to that on a homogeneous substrate (13%). These results strongly suggest that constrained FA maturation on 2-μm lines was not enough to induce a cell alignment distribution different than for cells on homogeneous substrates.

To evaluate further whether the orientation of FAs has a role in contact guidance for linear patterns larger than FAs, we constructed 2D density plots showing the distribution of the orientations of cells and FAs. The data show that strong cell alignment (i.e., contact guidance) does not correspond to a similarly strong alignment of mature FAs. Specifically, the density plots demonstrate that the distribution of orientation angles of FAs was scattered, showing all possible orientations (Figure 2B). This was again comparable to the orientations of FAs on homogeneous substrates (control), despite the increasing cell alignment for increasing *w*. Together, these results indicate that, at the investigated length scales, a mechanism independent of FA alignment determines contact guidance of myofibroblasts.

### Cells Minimize the Number of Non-adhesive Gaps to Bridge

In an earlier study, we demonstrated that contact guidance for cells constrained on single lines (i.e., in regime II) emerges from an entropy-mediated mechanism, driven by the non-thermal shape fluctuations of cells.^13^ We therefore asked whether such a mechanism may in fact be responsible for contact guidance over both regimes that we observed in the present study. To test this, we extended the statistical homeostatic mechanics framework developed by Shishvan et al.^14^ to model cell behavior. This framework was adopted because it captures an essential statistical feature of cell behavior: cells exhibit large fluctuations in terms of single-cell geometrical parameters (e.g., area, orientation, and aspect ratio), with clear trends that arise only when the statistics of these observables are compared for different experimental conditions. This is evident in our experimental observations (e.g., Figure 1D). The main hypothesis is that cells continually change their shape, orientation, and intracellular components by (1) minimizing their free energy over the short-term period (seconds), while (2) maintaining an average homeostatic free energy over the long-term period (minutes). Recent reports strongly suggest that these hypotheses hold for different cell types. For example, Suresh et al.^21^ adopted an analogous framework to predict stem cell differentiation.

Shishvan et al.^14^ computed the free energy associated with each cell configuration on a substrate, *f*, by accounting for the energy contribution of the stress fiber cytoskeleton $f_{cyto}$ and the passive elasticity of other cellular components ${\text{Φ}}_{elas}$. By simulating a large number of possible cell configurations, each with its free energies, previous studies have shown that this modeling framework can give critical insights into the ensemble behavior of adherent cells cultured on homogeneous substrates of varying stiffnesses^14^ or on single adhesive lines of different width.^13^ To model cell behavior on substrates with heterogeneous distribution of ligands as in the micropatterned substrates used in the present study, we extended this framework by considering the free-energy contribution of cell adhesions $f_{adh}$, which are approximated as linear springs with spring constants $k_{n}$ and $k_{a}$ on non-adhesive and adhesive areas, respectively (see Experimental Procedures for a detailed description). As a consequence of these energy contributions, cells elongate up to a certain extent to minimize the contribution of both the stress fibers and passive elastic components to the cell free energy, while at the same time avoid forming adhesions on non-adhesive areas of the substrates because they are associated with high values of free energy.

**Figure 3:**
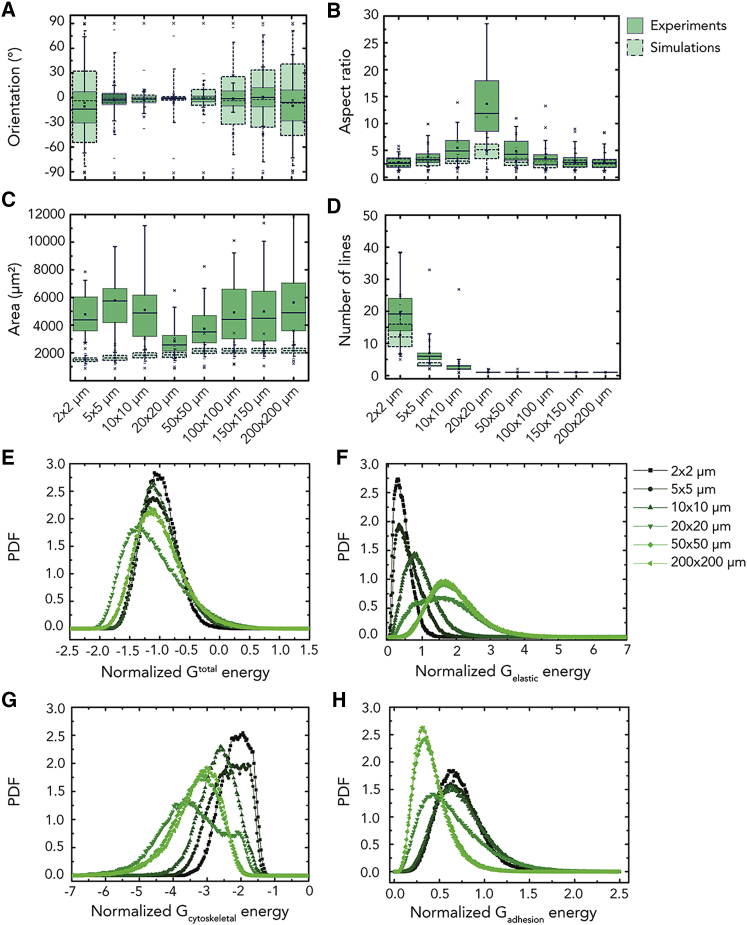
Modeling of Cellular Contact Guidance (A–D) The computational (dashed green boxes) results for myofibroblasts on parallel lines of fibronectin (2–200 μm) is compared against the corresponding experimental results (solid green boxes), in terms of the distribution of the cellular orientation (A), aspect ratio (B), area (C), and number of lines touched by single cells (D). The boxes of the boxplots represent the quartiles of the distributions, with the whiskers indicating the outliers in the experiments and the 5^th^ and 95^th^ percentiles of the distributions. (E–H) Probability density functions (PDFs) of the energies of myofibroblasts associated with the total Gibbs free energy (E), elasticity (F), cytoskeletal (G), and adhesion Gibbs free energy of the cell (H) are also shown. Each color represents a specific pattern of parallel lines (*w* × *s* μm). The energies are normalized by the energy of the free-standing cell. The data reported are results from three independent samples, and at least 60 cells were considered per condition.

A comparison between the computational and experimental results for myofibroblasts on substrates with lines of width *w* is shown in Figures 3 and S6. Remarkably, the simulations were able to model the two distinct regimes of cellular alignment observed experimentally (Figure 3A). Similar to the experimental observations, in regime I, cells increasingly aligned and elongated in the direction of the lines with increasing *w* up to a maximum at *w* = 20 μm (Figures 3A and 3B). It is worth emphasizing that these two regimes of cellular alignment emerged solely from the inclusion of $f_{adh}$, without adjusting any parameters for the other free-energy contributions. Furthermore, dynamic cellular protrusions were not simulated, leading to the expected underestimation of cell area compared to experimental data (Figure 3C). The simulations predicted that cells bridge a decreasing number of adhesive lines with increasing *w* (Figure 3D). This was in qualitative agreement with the experiments and indicated that cells thin along the direction perpendicular to the lines, thereby resulting in a reduced number of gaps they are in contact with. In regime II, cells spread on single adhesive lines (Figure 3D). At the onset of regime II (i.e., on 20-μm-wide lines), cells fit on single lines by thinning along the perpendicular direction, while strongly elongating and orienting in the direction of the lines (Figures 3A and 3B). With increasing *w*, the perpendicular thinning and longitudinal elongation necessary for cells to fit on single lines are smaller and therefore cells have increasing freedom to vary their main orientation, consistent with our earlier study.^13^ These results indicate that the hypotheses of free-energy minimization over the short timescale (seconds) and homeostasis over the long timescale (minutes) can explain the variation of cell morphologies observed for cells on substrates with alternating (non-)adhesive lines.

Given the agreement between the model and experimental observations, we proceeded to analyze the model features that are responsible for cell alignment in the simulations. The probability density functions of the total Gibbs free energy of cells in regime I (i.e., w< 20 μm) exhibited smaller interquartile ranges compared to cells in regime II (Figure 3E), suggesting that adhesion to multiple lines forces cells to explore less free-energy states and morphologies. In addition, we found increasing absolute values of the elastic Gibbs free energy for increasing line widths (Figure 3F), corresponding to an increase in cell deformation, which was compensated by a corresponding decrease in the cytoskeletal Gibbs free energy (Figure 3G). The Gibbs free energy of cell adhesions was relatively large for cells on multiple lines compared to that for the other line widths (Figure 3H). This suggests that cells reduce the adhesion energy by minimizing the contact with the non-adhesive lines by (1) reducing their total spread area (Figure 3C) or by (2) thinning in the direction perpendicular to the lines, resulting in overall cell alignment (Figures 3A and 3B). Interestingly, cells on 50 μm and 200 μm show almost identical probability density functions for the cellular free energies, although a much higher alignment was observed for the 50-μm-wide lines compared to the 200-μm ones (Figures 3A and 3E). Thus, it appears that, in regime II, a relatively high degree of alignment can be achieved without substantially perturbing the cell free energy. Notably, cells on 20-μm-wide lines have energy profiles that are remarkably different from all the others; the total and cytoskeletal Gibbs free energies are much lower, with wider interquartile ranges compared to the other line widths (Figures 3E and 3G). The Gibbs free energy of cells was affected by forcing cells to spread along single lines that are small compared to the size of the cell, inducing a very high degree of alignment. Taken together, the statistical thermodynamics framework suggests that contact guidance results from the tendency of cells to spread to minimize their cytoskeleton free energy while avoiding the formation of cell adhesions on non-adhesive lines. In other words, contact guidance may emerge from the maximization of actomyosin polymerization into stress fibers and minimization of FA formation on non-adhesive areas.

### The Non-adhesive Gap Size Determines the Degree of Cell Alignment

The insights from the model imply that cellular contact guidance may in fact be induced by the cells’ reluctance to bridge across non-adhesive gaps. The gaps between adhesive patterns have been previously suggested to act as a barrier to the formation of stable adhesions and protrusions.^22^ Live-cell imaging of myofibroblasts stained for the actin cytoskeleton (silicon rhodamine [SiR] actin) and FAs (GFP-Talin) for 24 h indicated that, while spreading, cells formed numerous protrusions and adhesions at both ends of the cell body, and smaller adhesions were also formed on the side of the cells, even after complete cell spreading and alignment. Moreover, cells were observed to form exploratory protrusions extending perpendicular to the lines, bridging the non-adhesive area, but these were often short lived and eventually retracted without initiating further cell spreading in that direction (see an example in Figure S3 and Video S1). As a result, over time, the cells elongated and oriented in the direction of the lines. This corroborates the notion that non-adhesive gaps between the adhesive lines hinder stable adhesions and thereby promote anisotropic cell spreading.

Video S1. A Representative Movie of a Spreading Myofibroblast (Stained for FAs, Magenta) on 20 × 20 μm Lines of Fibronectin (Red)

**Figure 4:**
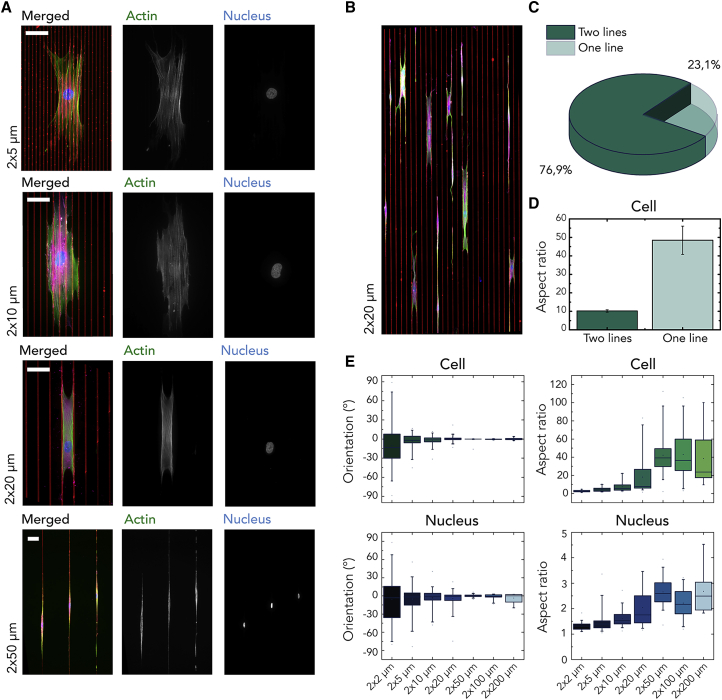
Influence of Inter-line Spacing on Cellular Alignment (A) Representative immunofluorescence images of myofibroblasts on fibronectin lines of *w* = 2 μm (red) and varying inter-line spacings (*s* = 2–50 μm), stained for the actin cytoskeleton (green), nucleus (blue), and FAs (magenta). Scale bars: 50 μm. (B) Cells on 2 × 20 μm (width × spacing) wide lines of fibronectin showing the transition from cells that bridge the non-adhesive gap to cells that do not bridge the non-adhesive gap. (C) The probability of cells spreading on either one or two lines on 2 × 20 μm patterns. (D) The corresponding aspect ratio of these cells. Data are represented as the mean ± standard error of the mean (SEM). (E) Quantitative analysis of cells (top) and nuclei (bottom) demonstrates that the inter-line spacing has a clear influence on the orientation and aspect ratio. The boxes show the quartiles of the distributions, with the whiskers indicating the outliers and the 5^th^ and 95^th^ percentiles of the distributions of the cell and nucleus orientation and aspect ratio. The data reported are results from three independent samples; at least 60 cells were considered per condition.

An implication of these experimental and computational findings is that contact guidance at length scales from the FA to the cell size is induced not by the width of the fibronectin lines *w* but by the inter-line spacing *s*. To verify this inference, we performed a new set of experiments where we varied *s* in the range of 2–50 μm while keeping *w* constant (2 μm). In myofibroblasts spreading over substrates with 2- to 20-μm-wide spacing, actin fibers were observed running in all directions throughout the cells, often crossing several adhesive and non-adhesive areas (Figure 4A). The inter-line spacing of *s* = 20 μm was found to be the transition point where myofibroblasts either spread between two lines (76.9% of all cells on this pattern) or spread along single lines (23.1%; Figures 4B and 4C). These two configurations were also reflected in the cell aspect ratio, which was much higher when cells stayed within single lines (Figure 4D). When *s* > 20 μm, the myofibroblasts failed to reach the neighboring adhesive line and became highly elongated, resulting in a pronounced alignment in direction of the lines (Figure 4E). This was accompanied by intracellular remodeling, such as increasing organization of the actin fibers and elongation of the nucleus. Remarkably, the transition at *s* = 20 μm coincides exactly with the transition from regime I to regime II that we saw earlier (Figure 1D), indicating that the latter is caused by the myofibroblasts’ (in)ability to bridge multiple lines separated by large gaps.

**Figure 5:**
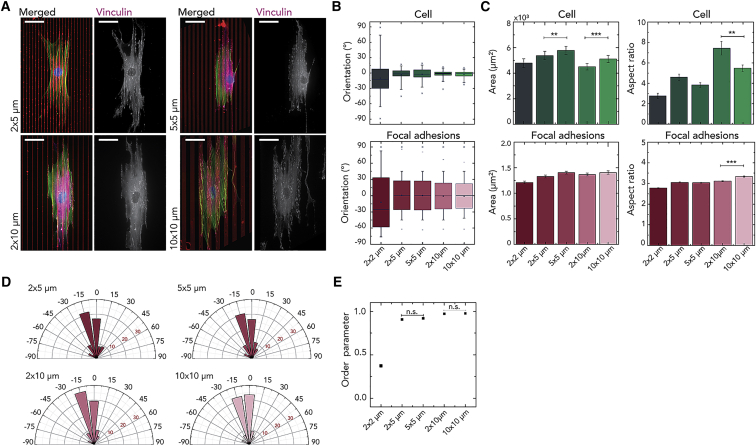
Influence of Line Width on the Cellular and FA Organization (A) Representative immunofluorescence images of myofibroblasts on fibronectin lines (red) of various and inter-line spacings (*w* = 2, 5, and 10 μm and *s* = 2, 5, and 10 μm) stained for the actin cytoskeleton (green), nucleus (blue), and FAs (magenta). Scale bars: 50 μm. The width of the lines has no effect on the cell orientation, although it affects cell area and aspect ratio (B and C). The corresponding FAs are also not affected by line width (B and D). (B) Boxplots represent the mean with 5^th^–95^th^ percentile range, with the whiskers indicating the outliers of the cell and FA orientation. (C) The mean ± SEM of the cellular and FA area and aspect ratio. ∗p < 0.05; ∗∗p < 0.01; ∗∗∗p < 0.001. The data reported come from three independent samples, and at least 20 cells were considered per condition. (D) The angular histogram of the FA orientations, where 0° represents the direction of the lines. (E) Measurements of the cell orientation order parameter Θ versus the line dimensions (width × spacing μm). The values of Θ range within 0–1, corresponding to random alignment when Θ = 0 and perfect alignment when Θ = 1. The results are from three independent experiments. At least 60 cells were considered per condition.

We also checked the effect of varying line widths *w*, but not the inter-line spacing *s*, by comparing patterns of 2 × 5 μm with 5 × 5 μm and 2 × 10 μm with 10 × 10 μm lines. Consistent with our hypothesis, varying the line width *w* alone had little effect on the orientation of cells and FAs (Figures 5B, 5D, and 5E). By contrast, inter-line spacing *s* has a clear effect on cell morphology, as quantified by their area and aspect ratio (Figure 5C). For increasing *s*, cells spread more; however, the FAs were not affected. By comparing the distribution median of the absolute value of cell angles (using the independent-samples median test), we observed that the difference between cells on substrates with different values of *w* and equal values of *s* was not statistically significant (Figure 5E). In contrast, changing values of *s* generally led to statistically different median values (p < 0.05 for all comparisons except for 2 × 5 μm and 2 × 10 μm, p = 0.54).

Taken together, our experiments combined with computational modeling support the idea that cell alignment induced by anisotropic cues larger than FAs and smaller than the typical cell length can result from the minimization of cellular adhesions on non-adhesive gaps—a phenomenon we define as “gap avoidance.”

## Discussion

The ability of cells to align in the direction of topographical or biochemical linear patterns, i.e., contact guidance, is critical in various physiological contexts.^23^, ^24^, ^25^ Previous studies on contact guidance have shown that anisotropic features created by microcontact printing can direct cell alignment.^16^^,^^26^, ^27^, ^28^, ^29^, ^30^ Although numerous studies have already extensively characterized the ability of a variety of cells to respond to anisotropic features present in their environment, a universal mechanism underlying this phenomenon remains elusive. Particularly, although spatial constriction of FAs^9^^,^^12^^,^^31^ or actin fiber structures (e.g., stress fibers, filopodia, lamellipodia)^6^^,^^30^^,^^32^^,^^33^ have been proposed to play a role in this process (Figure 5D), the contribution of each of these intracellular components to the emergence of contact guidance at intermediate length scales is still unclear.

Our combined experimental and computational work demonstrates the existence of two distinct regimes of cellular alignment, governed by the length scale of the anisotropic cue (Figure 1D). These distinct regimes have not been observed previously, as previous studies have focused on limited ranges of line widths, in some cases using microfabricated substrates that present confounding additional cues in the third dimension as well, such as grooves and ridges (see Tamiello et al.^11^ for a review). For *w* <20 μm, myofibroblasts aligned parallel to the lines, bridging multiple lines. Decreasing *w* results in reduced cellular alignment, and at *w* = *s* = 2 μm, contact guidance effect is greatly decreased. A recent study by Ramirez-San Juan et al.^30^ has observed a much stronger alignment of NIH 3T3 fibroblasts on parallel fibronectin lines with *w* = *s* = 2 μm than that of the myofibroblasts in our study. The discrepancy between this previous study and our observations might result from the differences in cell size. Indeed, Ray et al.^9^ showed that the degree to which different cell types respond to anisotropic substrates can vary significantly, and 3T3 fibroblasts are typically much smaller than human myofibroblasts.^34^ The model presented in the present study suggests that the energy penalty of a gap size has in proportion a stronger effect if the cell size is smaller. Thus, the stronger alignment of 3T3 cells compared to that of myofibroblasts in response to 2 × 2 μm lines might derive from the different sizes of these two cell types. To test whether this model prediction is worth considering, we analyzed the alignment of human cardiomyocyte progenitor cells (CMPCs) (average spread length 100 μm) on our fibronectin lines. We found that CMPCs showed a very similar contact-guidance response to that of myofibroblasts (average spread length 180 μm), as expected (Figure S4). Moreover, the transition from cell alignment on single lines to alignment on multiple lines for CMPCs occurs at *s* = 10 μm, smaller than for myofibroblasts (*s* = 20 μm), in qualitative agreement with our model prediction. A future comparative study including different cell types with systematically varying spacing between adhesive lines could further elucidate the role of cell type and size on contact guidance.

We observed that the two regimes of cellular orientation were also reflected in the orientation of the individual actin fibers and nucleus, although the two regimes were less clear for FAs. Previous studies demonstrated that FA dynamics are affected by the geometric dimensions of anisotropic substrates.^9^^,^^12^^,^^31^^,^^35^ In addition, higher levels of aligned mature adhesions were observed along lines or microgrooves. However, a closer examination of the FAs of cells in regime I revealed that many FAs were not fully aligned in direction of the lines, although the cells showed a clear increase in alignment for increasing line widths (Figures 1A and 2B). Given that the typical FA size is larger than 2 μm (Figure 2D), we expected that, on 2-μm-wide lines, FAs would orient and elongate in direction of the lines. However, we found that FAs were non-aligned, smaller, and less elongated for *w* = 2 μm compared to on homogeneously coated substrates (Figures 2C and 2D). These results indicate that, at intermediate-length scales, contact guidance arises from a mechanism other than constrainment of FA alignment.

In a seminal study on contact guidance, Dunn and Heath^6^ postulated that this phenomenon arises when non-adhesive gaps are wide enough to cause a mechanical restriction for the formation of actin protrusions, causing cells to align in direction of the lines. Although the alignment of filopodia and lamellipodia^22^^,^^30^^,^^33^ has been proposed to play a role in this process, the exact contribution of these protrusions is not fully clear. To determine the effects of non-adhesive gaps on cell alignment, we varied *s* and showed that cells can still form protrusions to bridge the non-adhesive gaps of length scales much larger than the characteristic length of filopodia (∼5 μm; Figures 4A and 4B).^36^ This confirms that the formation of actin protrusions was not restricted and mechanical restriction of the formation of actin protrusions is not responsible for cellular contact guidance. Additionally, previous studies have also found that blocking filopodia formation did not affect contact guidance.^37^ Figure 5B shows that cellular alignment is not significantly affected by increasing *w* but is enhanced by increasing *s*, indicating that the inter-line spacing *s* is a more important parameter in guiding cellular alignment than line width *w*.

To understand the mechanisms determining contact guidance, several computational models have been developed that account for the (re)organization of intracellular structural components, i.e., FAs, actin stress fibers, and actin protrusions. The computational model of Loosli et al.^38^ successfully predicted experimental observations reported in the literature,^27^ but the hypotheses at the basis of the model were phenomenological. Similarly, Vigliotti et al.^39^ could predict contact guidance for cells on nanogrooved substrates^40^ with a cooperative feedback model for the interplay between FA formation and actin stress fibers development. However, this approach can only be used for groove sizes that are very small compared to the cell size. Recently, an increasing number of computational models were established for cell spreading on substrates, based on the hypothesis that free energy minimization drives this biological process.^41^, ^42^, ^43^, ^44^ This hypothesis successfully explained the spreading of cells on smooth substrates^42^ or patterned islands.^43^ However, these models were unable to account for the full range of experimental observations, except by including a fitting (phenomenological) parameter for the cell fluctuations. Shishvan et al.^14^ recently postulated that the fluctuations in cell morphology are constrained by the fact that cells remodel their shape and intracellular structures to maintain an overall homeostatic state over the long-term period. Here, we extended this framework by taking the adhesive properties of the substrate into account. With this inclusion, the experimentally observed regimes of cellular alignment and aspect ratio were successfully captured.

The proposed computational framework does not include the directionality of FA formation or the dynamics of actin-rich protrusions. Still, the model was able to fully predict the two regimes of cellular alignment, clearly suggesting that alignment of FA or protrusions are not necessary ingredients for the alignment of cells in response to anisotropic adhesive lines, in agreement with our experimental results. The model further suggests that the tendency of cells to align in the direction of the lines is a direct consequence of minimizing the number of non-adhesive gaps to bridge in order to reduce the contribution of the cellular adhesion energy to the free energy. This is consistent with the observation of Romsey et al.,^29^ who demonstrated that the number of lines a cell contacts affects the fidelity of contact guidance. Indeed, the average number of adhesive gaps to bridge or number of adhesive lines that each cell contacted decreased with increasing *s* (Figure 3D). Altogether, our study showed that cellular alignment is determined by the adhesion properties between the cells and the non-adhesive gaps. Future studies should be directed to dissecting in greater detail the role of dynamic processes, such as actin protrusions, in this gap-avoidance behavior. Moreover, it will be instructive to explore the role of these cellular processes in the transition between adhesion-mediated and gap-mediated contact guidance, for example, by systematically monitoring cellular responses above and below 2 μm, which is technically impossible using our current experimental approach.

To summarize, we have shown that alignment of myofibroblasts can be induced by anisotropic geometrical cues ranging from micron to hundreds-of-micron scale. Our experimental and computational results suggest that cell alignment at these length scales emerges from the tendency of cells to elongate and maximize actin polymerization, while avoiding the formation of cellular adhesions on non-adhesive gaps (“gap avoidance”). This is a generic biophysical mechanism underlying the morphological fluctuations of cells that does not require specific biochemical regulation or molecular pathway. Thus, this understanding not only offers an attractive, alternative explanation for how substrate anisotropy regulates the cellular orientation response but can also be relevant for devising new experimental strategies for directing tissue morphogenesis and regeneration.

## Experimental Procedures

### Design and Fabrication of Stamps

Microcontact printing was used to pattern adhesive lines ranging from 2 μm to 200 μm. The micropatterned substrates were fabricated via standard photolithography techniques, according to previous protocols.^45^ Briefly, the desired features were generated onto a silicon master by deep reactive-ion etching (Philips Innovation Services, Eindhoven, the Netherlands) from a chromium photomask (Toppan Photomask, Corbeil Essonnes, France). The silicon surface was passivated with a fluorosilane, and microstamps were obtained by molding the silanized silicon master with polydimethylsiloxane (PDMS) (Sylgard 184; Dow Corning) and curing agent (10:1), which was cured at 65°C overnight. The cured PDMS stamps containing the desired features were then peeled off from the master.

### Microcontact Printing

For microcontact printing, the stamps were cleaned by sonicating in 70% ethanol for 30 min and dried using compressed air. The structured surface of the PDMS stamps were incubated for 1 h at room temperature with a 50 μg/mL rhodamine fibronectin (FN) solution (Cytoskeleton). The substrates for printing were flat PDMS-coated glass coverslips that were oxidized in a UV/Ozone cleaner (PDS UV-ozone cleaner; Novascan, Ames, IA) for 8 min just before use. The FN-coated stamps were dried under sterile air flow and gently deposited on the substrates for 15 min at room temperature. Uncoated regions were blocked by immersing the micropatterned coverslips for 5 min in a 1% solution of Pluronic F-127 (Sigma-Aldrich). Finally, the coverslips were three times washed with phosphate-buffered saline (PBS) and stored in PBS at 4°C before use. As a control substrate, a homogeneous fibronectin coating was obtained using a flat PDMS stamp.

### Cell Culture

Human vena saphena cells (HVSCs) were harvested from the vena saphena magna obtained from patients according to Dutch guidelines of secondary used material and have previously been characterized as myofibroblasts.^46^ The myofibroblasts were cultured in advanced Dulbecco’s modified Eagle’s medium (Invitrogen, Breda, the Netherlands) supplemented with 10% fetal bovine serum (Greiner Bio-one), 1% penicillin/streptomycin (Lonza, Basel, Switzerland), and 1% GlutaMax (Invitrogen). Only cells with a passage lower than 7 were used in this study. To avoid effects on cell alignment deriving from cell-cell contacts, the microcontact printed substrates were seeded with a cell density of 2,000 cells/cm^2^. The myofibroblasts were cultured for 24 h at 37°C and 5% CO_2_ on these substrates.

Human fetal cardiomyocyte progenitor cells (CMPCs) were isolated and cultured as described previously.^47^^,^^48^ In this study, the L9TB CMPC cell line, a kind gift from Prof. Marie-José Goumans (Leiden UMC), was used, immortalized by lentiviral transduction of hTert and BMI-1. The CMPCs were cultured in SP++ growth medium consisting of M199 (Gibco)/EGM2 (3:1) supplemented with 10% (v/v) FBS (Greiner bio-one), 1% (v/v) non-essential amino acids (Gibco), and 1% (v/v) penicillin/streptomycin (Lonza) on 0.1% (w/v) gelatin (Sigma-Aldrich)/PBS (Sigma)-coated flasks until transfer onto the microcontact printed substrates. The CMPCs were seeded with a cell density of 2,000 cells/cm^2^ and were used with passage number 44–46. The CMPCs were cultured for 24 h at 37°C and 5% CO_2_ on these substrates.

### Immunofluorescence Labeling

After culture on the micropatterned substrates, the myofibroblasts were washed with PBS and fixed with 3.7% formaldehyde in PBS (Sigma-Aldrich) for 15 min at room temperature. The cells were permeabilized with 0.5% Triton X-100 (Merck) in PBS for 10 min at room temperature. Fixed cells were incubated for 30 min with 4% goat serum in PBS in order to block non-specific binding. Subsequently, samples were incubated with Alexa Fluor 647 goat anti-mouse (Molecular Probes) diluted at 1:500 and Phalloidin-Atto (15500, Phalloidin-Atto 488, Sigma-Aldrich) diluted at 1:200 for staining the actin cytoskeleton. Finally, the samples were incubated with DAPI (Sigma-Aldrich) diluted at 1:500 for 1 h at room temperature for immunofluorescence of the nucleus and mounted onto glass slides using Mowiol (Sigma-Aldrich). Fluorescence images were obtained with an inverted microscope (Zeiss Axiovert 200M equipped with an AxioCam HR camera; Zeiss, Sliedrecht, the Netherlands) using a 20×/0.25 Ph1 (0.68 μm/pixel) or a 40×/0.95 (0.16 μm/pixel) objective.

### Live-Cell Imaging

For time-lapse imaging of cell spreading, microcontact printed coverslips were mounted in a custom-made chamber. Prior to the live-cell experiments, the cells were transduced with Talin-GFP CellLight BacMam 2.0 (Life Technologies) according to manufacturer recommendations to stain for the FAs. Culture flasks were incubated with approximately 30 BacMam particles/cell for at least 24 h. Then, the cells were trypsinized and resuspended in 100 nM SiR-actin (Cytoskeleton) for 45 min at 37°C. This cell suspension was centrifuged for 7 min at 1,000*g* and resuspended in medium. We added 100 nM of SiR-actin to the medium and seeded the cells at 2,000 cells/cm^2^ on the printed substrate. After 30 min incubation to allow cell adhesion, the sample was placed in the microscopy incubator under controlled temperature (37°C) and CO_2_ (5%) conditions. Data were collected approximately 90 min after cell seeding by imaging at 512 × 512 pixels every 15–20 min over 24 h with a confocal microscope (Leica TCS SP 5 confocal microscope equipped with a Leica MC170 HD camera) using a 20×/0.7 (0.32 μm/pixel) objective.

### Image Analysis

The cellular, nuclear, and FA morphological parameters were assessed from Phalloidin-Atto, DAPI, and vinculin-stained images, respectively, that were analyzed using a custom-built script publicly available either in Mathematica (Wolfram Research, Mathematica, Version 11.1, Champaign, IL, USA, 2017)^17^ or in MATLAB (The Mathworks, Natick, MA, USA; https://gitlab.tue.nl:443/stem/sfalab/tree/v0.01). Briefly, the images of cells, nuclei, and FAs were binarized and fitted with an ellipse using a least-square algorithm. This was used to quantify the orientation, which was defined as the angle θ between the major axis of the ellipse and the direction of the patterned lines, where θ = 0° represents the direction of the lines. The cell orientation order can be characterized by the order parameter Θ:^49^

(Equation 1) $$\text{Θ}=\sqrt{{\langle \text{cos}\left(2\theta \right)\rangle }^{2}+{\langle \text{sin}\left(2\theta \right)\rangle }^{2}},$$

where <.> denotes ensemble averaging over all measurements. The values of Θ range from 0, representing random alignment, to 1, representing perfect alignment. To represent the shape of the cells, nuclei, and FAs, we computed the aspect ratio (i.e., the ratio of the major axis to the minor axis) and area. For the quantification of the FA orientation, only elongated FAs (aspect ratio larger than 1.6) were taken into account. Actin fiber orientation was quantified using a fiber orientation algorithm, based on the work by Frangi et al.^50^

### Statistical Analysis

The data were obtained and pooled from at least three independent experiments (substrates), each containing multiple (4–8) micropatterns. For each condition, we analyzed at least 60 cells unless indicated otherwise. To assess differences between the different line widths on the morphological features of the FAs, one-way ANOVA with a Bonferroni post hoc test was used.

### Modeling

To get a better understanding of the mechanisms determining the response of myofibroblasts to multiple adhesive (fibronectin) lines alternated with non-adhesive lines, we extended a recently developed statistical thermodynamics framework for cells.^14^ In this study, we extended this framework to simulate cells spreading on multiple (non-)adhesive lines of different width. In what follows, the main characteristics of this framework are briefly summarized, together with the features of the model extension. A complete description of the model and numerical implementation can be found in the original model^14^ and in the Supplemental Experimental Procedures.

A system comprising a single cell in contact with a substrate and surrounded by a nutrient bath was analyzed. This is an open system because the cell exchanges molecular species with the surrounding nutrient bath. Over the short-term period of seconds, this exchange is negligible and the system can be assumed as closed. Therefore, the state of the intracellular molecules over this short-term period can be predicted by Gibbs free-energy minimization as in classical statistical mechanics. Over the long-term period of minutes to hours, the exchange of molecules through the cellular membrane is not negligible. Over this time span, the cell is then out of equilibrium in terms of classical statistical mechanics and exhibits fluctuations in terms of its observables. However, it is known that cells, via intracellular biochemical processes, actively strive to maintain an average homeostatic number of molecular species within their body.^5^ This, in terms of Gibbs free energy, translates to enforcing the constraint that the average Gibbs free energy of the system is constant over the long-term period.^14^ By enforcing that the molecular species within the cell body minimize their Gibbs free energy over the short-term period (seconds), while the average Gibbs free energy of the system is constant over the long-term period (minutes to hours), the probability distributions of the cell orientation, aspect ratio, etc. can be obtained. Due to the cell movement and exchange of nutrients, these observables will exhibit fluctuations much larger than thermal fluctuations but proportional to a homeostatic temperature, a concept that was introduced in Shishvan et al.^14^

Similar to previous studies,^13^^,^^14^ the Gibbs free energy of a cell configuration (j) was defined as

(Equation 2) $$G^{\left(j\right)}=\int_{V_{cell}}fdV,$$

where $V_{cell}$ denotes the cell volume and *f* is the specific Helmholtz free energy of the cell. This last term was computed as

(Equation 3) $$f=f_{cyto}+{\text{Φ}}_{elas}+f_{adh},$$

where the terms $f_{cyto}$ and ${\text{Φ}}_{elas}$, respectively associated with the stress fiber and cell passive components, were defined as in Shishvan et al.^14^ Compared to previous studies, here, the term $f_{adh}$ was added to consider the Helmholtz free energy of cell adhesions. Specifically, cell adhesions on the substrate were approximated as linear springs, and it was assumed that springs associated with non-adhesive parts of the substrate have a very low stiffness $k_{n}$ compared to the stiffness $k_{a}$ of springs on adhesive areas (*k*_*n*_ ≪ *k*_*a*_). From this, it follows that the Helmholtz free energy of a cell adhesion at a cell material point x can be computed as

(Equation 4) $$f_{adh}=\{\begin{array}{cc} \frac{{\left(F\left(x\right)\right)}^{2}}{2k_{a}}, & \text{if }x\text{ is adhesive},\\ \frac{{\left(F\left(x\right)\right)}^{2}}{2k_{n}}, & \text{if }x\text{ is non}-\text{adhesive}, \end{array}$$

where F(x) is the magnitude of the force (in material point x) that the linear spring has to exert to equilibrate the residual forces resulting from the interaction between stress fibers and cell passive components. We observe that, from *k*_*n*_ ≪ *k*_*a*_, it follows $\frac{{\left(F\left(x\right)\right)}^{2}}{2k_{n}}\gg \frac{{\left(F\left(x\right)\right)}^{2}}{2k_{a}}$. Consequently, cell configurations without cell adhesions on non-adhesive points have much lower values of Helmholtz (and Gibbs) free energy and are considerably more likely to occur. Therefore, the approach efficiently models the preference of cells to form and mature adhesions only on adhesive lines.

### Material Parameters for Myofibroblasts

A single set of material parameters was adopted and unchanged for all simulations of this study. Most of the model parameters describe inherent properties of stress fiber proteins that are not expected to differ among cell types. Differences among cell types are recapitulated in terms of the stress fiber protein volume fraction $F_{0}$, the passive elastic properties described by the shear modulus of the cell (μ), the in-plane bulk modulus of the cell *k*, and *m* (a material constant that describes the nonlinearity of the deviatoric elastic cell response). Overall, increasing the term $F_{0}$ causes a widening of the Gibbs free-energy distribution and an increase of the homeostatic area of a cell on a homogeneous substrate, although an increase of the cellular elastic properties has the opposite effect. In addition to these parameters, the homeostatic area can also change because of the inherent size of a cell type. In the model, this parameter is represented by the scaling parameter $l_{w}$. For more details on the model parameters, we refer the reader to the Supplemental Information and to previous studies.^14^^,^^51^

The parameters adopted in the present study are based on validations performed in previous works.^14^^,^^52^ In particular, all parameters except for $k_{a}$ and $k_{n}$ were taken in agreement with Shishvan et al.^14^ ($k_{a}$ and $k_{n}$ were not present in this previous study). Shishvan et al. demonstrated that, with these parameters, the model can predict morphological features (e.g., area and aspect ratio) of smooth muscle cells spreading on substrates with different stiffness. Myofibroblasts have very similar mechanical properties as smooth muscle cells, and therefore, the same parameters were adopted in the present study. The parameter $k_{a}$ was then chosen such that the focal adhesion energy computed for cells on homogeneous and stiff substrates was similar to the energy reported in McEvoy et al.,^52^ who analyzed the focal adhesion free energy for cells on substrates with different rigidities. Finally, $k_{a}$/$k_{n}$ = 100 was chosen such that adhesions formed on non-adhesive areas were hardly observed in the simulations. A list of the material parameters is given in Table S1.

### Computational Simulation

The cell in the initial, undeformed (circular) configuration was discretized with three-noded triangular elements. Different configurations (j) were then obtained by varying the displacements $u^{\left(j\right)}$, which uniquely identify the strains in the cell. From these values, by considering the chemical equilibrium in the cell body, $f_{cyto}$ and ${\text{Φ}}_{elas}$ are computed, as well as the passive stress within the cell and the stress fiber stress. Given these passive and active stress values, the residual traction forces $T\left(x^{\left(j\right)}\right)$ can be identified at each node, together with the consequential values of $F\left(x^{\left(j\right)}\right)=-T\left(x^{\left(j\right)}\right)$. From these forces, the Helmholtz free energy $f_{adh}$ and the total Gibbs free energy can be computed. For obtaining the statistics of cell shapes on a substrate, the homeostatic Gibbs free energy and the associated homeostatic temperature were first computed iteratively using Metropolis algorithm, analyzing 2,000,000 cell-shape perturbations. The same procedure was then repeated to simulate cells on microcontact printed substrates. This was achieved without changing the material parameters but by changing the homeostatic temperature of the system to enforce the condition that the average Gibbs free energy of a cell in every substrate is equal to the homeostatic energy found for the free-standing cell. See Supplemental Experimental Procedures for a complete description of the simulations.

The morphologies of representative cells from the simulations and experiments are compared in Figure S6. In general, the cellular shapes obtained with the computational simulations are comparable to those experimentally observed. For each cell shape resulting from the simulations, the cellular orientation and aspect ratio were obtained by fitting the outline of the cell with the best-fitting ellipse (in a least-square sense), as in the experiments. The statistics of these simulations are reported in Figure S5.

### Data and Code Availability

Codes used to analyze the experimental images are publicly available at https://gitlab.tue.nl:443/stem/sfalab/tree/v0.01 and https://doi.org/10.1371/journal.pone.0195201. Codes used to perform the computational modeling of cells are publicly available at https://zenodo.org/record/3710132. All data associated with the study are included in the paper and the Supplemental Information or from the lead author upon reasonable request.

## Supplemental Information

Document S1. Supplemental Experimental Procedures, Figures S1–S5, and Table S1

Document S2. Article plus Supplemental Information
