# Supplementary material for: Cellular Contact Guidance Emerges from Gap Avoidance
Source: Cell Rep Phys Sci. 2020 May 20;1(5):100055. doi: 10.1016/j.xcrp.2020.100055 (PMC7357833; doi:10.1016/j.xcrp.2020.100055)
Supplement: Document S1. Supplemental Experimental Procedures, Figures S1–S5, and Table S1 [file mmc1.pdf]

Cell Reports Physical Science, Volume *1*

Supplemental Information

Cellular Contact Guidance

Emerges from Gap Avoidance

Antonetta B.C. Buskermolen, Tommaso Ristori, Dylan Mostert, Mark C. van Turnhout, Siamak S. Shishvan, Sandra Loerakker, Nicholas A. Kurniawan, Vikram S. Deshpande, and Carlijn V.C. Bouten

Supplemental Experimental Procedures

Modeling framework

To predict the shape and orientation of cells on multiple adhesive lines of fibronectin, we extended the statistical framework of Shishvan et al. [1], which is here briefly described. In what follows, we refer to: "the cell", to indicate a generic cell to model; "the substrate", as a generic substrate with alternating (non-)adhesive lines; "cellular configuration", to indicate the mapping of the cell material points on the substrate; and "the system", as the system composed of the cell and the substrate.

Characterization of a configuration

The cell was approximated as a two-dimensional body in the $x_1 - x_2$ plane, with through-thickness stress $\Sigma_{33} = 0$. $\boldsymbol{X}$ indicates the coordinates of each material point of the cell in the undeformed configuration, omitting the index of all different material points for brevity. The cell in the undeformed configuration was assumed cylindrical, with radius R_0 and thickness $b_0 = R_0/5$. The other configurations were characterized by the displacements of each material point $\boldsymbol{u}^{(j)}(\boldsymbol{X}) \equiv \boldsymbol{u}^{(j)}$, such that $\boldsymbol{x}^{(j)} = \boldsymbol{X} + \boldsymbol{u}^{(j)}$ are the coordinates of the material points of the cell in the configuration with index (j).

The probability of a configuration

By assuming a rigid and purely elastic substrate not deformed by cells, the Gibbs free-energy of a configuration (j) was defined as:

$$G^{(j)} = \int_{V_{cell}} f dV, \tag{1}$$

where V_{cell} denotes the volume of the cell and f is the specific Helmholtz free-energy of the cell. As described in Shishvan et al. [1], by assuming Gibbs free-energy minimization over the short-term period (seconds) and homeostasis over the long-term period (minutes), it can be derived that a configuration (j) has probability

$$P_{eq}^{(j)} = \frac{1}{Z} \exp\left[-\zeta G^{(j)}\right], \tag{2}$$

where $Z = \sum_j \exp\left[-\zeta G^{(j)}\right]$ is the partition function and ζ is a constant such that

$$\sum_j P_{eq}^{(j)} G^{(j)} = \frac{1}{Z} \sum_j G^{(j)} \exp\left[-\zeta G^{(j)}\right] = \bar{G}. \tag{3}$$

Here, $\bar{G}$ is the homeostatic Gibbs free-energy, corresponding to the free-energy of a free-standing cell (i.e. isolated, not interacting with any substrate). The average magnitude of the fluctuations of the energy of a cell on a specific substrate is proportional to $1/\zeta$, which is referred to as homeostatic temperature [1]. This quantity is a characteristic of the interaction between the cell and the substrate, and was computed for each different substrate.

Definition of the specific Helmholtz free-energy f

In Shishvan et al. [1], the Helmholtz free-energy f was computed as $f = f_{cyto} + \Phi_{elas}$, where f_{cyto} and Φ_{elas} are the specific Helmholtz free-energies associated with stress fibers and other cellular passive components (e.g. cell membrane), respectively. Here, we added a term f_{adh} corresponding to the specific Helmholtz free-energy of cell adhesions to account for the heterogeneity of the substrate in exam, where cells can easily form adhesions on the adhesive lines, while the formation of cell adhesions on the non-adhesive lines is obstructed:

$$f = f_{cyto} + \Phi_{elas} + f_{adh}. \tag{4}$$

The terms f_{cyto} and Φ_{elas} were computed as in [1], by using the model of Vigliotti et al. [2] to compute f_{cyto}, and a two-dimensional adaptation of the Ogden hyperelastic model [3] to calculate Φ_{elas}. In what follows, the computation of these terms is summarized, followed by the definition of the term f_{adh} introduced in the present study.

The specific Helmholtz free-energy of stress fibers f_{cyto}

Vigliotti et al. [2] envisioned stress fibers as formed by a number of functional units with reference length l_0. The quantities describing stress fibers are defined in a representative volume element (RVE) that, in its undeformed configuration, is defined as a cylinder with the axis along the thickness of the two-dimensional cell, radius $n^R l_0/2$, and thickness b_0 (such as the cell). Stress fibers initiate from the center of this RVE, oriented at angles ϕ $(-\pi/2 \leq \phi < \pi/2)$ with respect to the axis x_1. In the undeformed state, n^R functional units are present in the stress fibers in each direction while, along the thickness, n_s layers of identically distributed stress fibers are present. Given an infinitesimal area of the cylinder of the RVE oriented at an angle ϕ, the number of stress fibers crossing this area is $d\Pi = \eta d\phi$, such that $\eta(\phi)$ is defined as the angular stress fiber concentration.

When a nominal strain $\varepsilon_{nom}(\phi)$ is applied along the normal of the surface dA, the stress fibers in that direction deform and, in response, remodel. In particular, due to the deformation of stress fibers and their functional units, the number of these functional units increases (in case of tensile strain) or decreases (for compressive strain) till their deformed length reaches an optimal value l_{ss}. It follows that, in addition to the nominal strain of the RVE, we can define a nominal strain $\tilde{\varepsilon}_{nom}(\phi)$ for the stress fiber functional units, relative to l_0. A relationship between $\varepsilon_{nom}(\phi)$ and $\tilde{\varepsilon}_{nom}(\phi)$ can be found by observing that $n_0 \equiv n^R [1 + \varepsilon_{nom}]$ functional units of length l_0 are necessary to cover the deformed stress fiber length $n^R l_0 [1 + \varepsilon_{nom}]$. In contrast, if n functional units are present along such length, each of them has the length $n^R l_0 [1 + \varepsilon_{nom}]/n$, and thus

$$\tilde{\varepsilon}_{nom} = \frac{n^R l_0 [1 + \varepsilon_{nom}]}{l_0 n} - 1 = \frac{n_0}{n} - 1. \tag{5}$$

At steady-state each unit has length l_{ss}, thus

$$\tilde{\varepsilon}_{nom} = l_{ss}/l_0 - 1 \equiv \tilde{\varepsilon}^{ss}_{nom}, \tag{6}$$

and

$$n^{ss} = n^R \frac{[1 + \varepsilon_{nom}]}{[1 + \tilde{\varepsilon}^{ss}_{nom}]} \tag{7}$$

are the nominal strain and number of stress fiber functional units along a direction ϕ at steady-state.

At this point, we define

$$N_b \equiv \int_{-\pi/2}^{\pi/2} \eta n^{ss} d\phi \tag{8}$$

as the total number of stress fiber functional units within the entire RVE at steady-state. At the same time, within the same RVE, there are a number of unbound functional units N_u, such that the total number of functional units within the RVE is $N_T = N_b + N_u$. In the time-scale analyzed in this study, the total number of functional units within the cell normalized over the cell volume was assumed to be constant and it was denoted with $N_0 \equiv \int_{V_{cell}} N_T dV/V_0$, where V_{cell} and V_0 represent the volume of the cell in the deformed and undeformed configurations, respectively. Given the conservation of this value, normalized quantities were defined as $\hat{N}_u \equiv N_u/N_0$, $\hat{N}_T \equiv N_T/N_0$, $\hat{N}_b = N_b/N_0$, $\hat{\eta} \equiv \eta n^R/N_0$, and $\hat{n}^{ss} \equiv n^{ss}/n^R$.

When the cell is at steady-state, $\dot{\varepsilon}_{nom} = \dot{\tilde{\varepsilon}}_{nom} = 0$, and from the assumption that stress fibers exert tension with mechanisms similar to striated muscle, it follows that in these conditions they exert the maximum stress σ_{max}. For these conditions, the chemical potential associated with each functional unit that is part of a stress fiber is

$$\mathcal{X}_b = \frac{\mu_b}{n^R} + kT \ln \left[\left(\frac{\pi \hat{\eta} \hat{n}^{ss}}{\hat{N}_u \left(1 - \frac{\hat{\eta}}{\hat{\eta}_{max}}\right)} \right)^{\frac{1}{n^{ss}}} \left(\frac{\hat{N}_u}{\pi \hat{N}_L} \right) \right] \tag{9}$$

where the first term on the right-hand side accounts for the enthalpy μ_b of n^R functional units, while the second term considers the entropy of mixing between packets of unbound and bound stress fiber proteins with sites for bound proteins, and the entropy of mixing between unbound proteins and lattice sites. For a complete derivation of this chemical potential, we refer the reader to [1]. In Eq. (9), $\hat{\eta}_{max}$ represents the maximum value of $\hat{\eta}$ such that all available lattice sites N_L are occupied, while $\hat{N}_L \equiv N_L/N_0$. The enthalpy μ_b of bound proteins can be quantified as

$$\mu_b = \mu_{b0} - \sigma_{max} \Omega (1 + \tilde{\varepsilon}^{ss}_{nom}), \tag{10}$$

where μ_{b0} is the internal energy of n^R bound stress fiber functional units and $\Omega \equiv A_0 n^R l_0$ is the volume of n^R functional units of length l_0. The chemical potential associated with every unbound functional unit is

$$\mathcal{X}_u = \frac{\mu_u}{n^R} + kT \ln \left[\frac{\hat{N}_u}{\pi \hat{N}_L} \right], \tag{11}$$

with μ_u the internal energy of unbound functional units potentially forming n^R stress fiber units. Now, given $\rho_0 \equiv N_0/V_{cell}$, the specific Helmholtz free-energy accounting for the stress fiber cytoskeleton is

$$f_{cyto} = \rho_0 \left(\hat{N}_u \mathcal{X}_u + \int_{-\pi/2}^{\pi/2} \hat{\eta} \hat{n}^{ss} \mathcal{X}_b d\phi \right). \tag{12}$$

This quantity can be computed, given the previous definitions, once calculated $\hat{\eta}$ and $\hat{N}_u$. Note that f_{cyto} has to be computed for equilibrium morphological microstates, which means $d\mathcal{G}^{(j)} = 0$. It can

be demonstrated [1] that this requirements translates to the constraints of chemical and mechanical equilibria $\mathcal{X}_b = \mathcal{X}_u$ and $\Sigma_{ij} = 0$, where Σ_{ij} is the total Cauchy stress, characterized as a summation between the stress fiber stress, the stress from the cellular adhesions, and the passive stress of the other passive cellular components. From the chemical equilibrium $\mathcal{X}_b = \mathcal{X}_u$ and Eqs. (9) and (11), an expression for $\hat{\eta}$ in terms of $\hat{N}_u$ follows:

$$\hat{\eta} = \frac{\hat{N}_u \hat{\eta}_{max} \exp\left[\frac{\hat{n}^{ss}(\mu_u - \mu_b)}{kT}\right]}{\hat{n}^{ss}\hat{\eta}_{max} + \hat{N}_u \exp\left[\frac{\hat{n}^{ss}(\mu_u - \mu_b)}{kT}\right]}. \tag{13}$$

The normalized value $\hat{N}_u$ can be computed by recalling that $N_T = N_u + N_b$ is the total number of functional units in a material point x_i, while $N_0 V_0 / V_R$ is the total number of functional units that can be formed within the cell. These two quantities are related as

$$\frac{N_0 V_0}{V_R} = \int_{V_{cell}} N_T dV = \int_{V_{cell}} N_u dV + \int_{V_{cell}} N_b dV. \tag{14}$$

The integral $\int_{V_{cell}} N_u dV$ can be easily computed because, from the chemical equilibrium, it follows that $\mathcal{X}_u$ is constant across the cell and therefore, due to Eq. (11), also N_u is constant. Now, from the definitions of $\hat{N}_u = N_u/N_0$ and $\hat{N}_b = N_b/N_0$ and (14), we have

$$1 = \hat{N}_u + \frac{1}{V_0} \int_{V_{cell}} \int_{-\pi/2}^{\pi/2} \hat{\eta}\hat{n}^{ss} d\phi dV, \tag{15}$$

from which $\hat{N}_u$ can be computed, since it is the only unknown (given Eq. (13)).

The specific Helmholtz free-energy of cellular passive components Φ_{elas}

The specific Helmholtz free-energy of passive components of the cell was modeled with a modified version of the Ogden hyperelastic strain energy density function, with the modification necessary to specialize the formulation to a two-dimensional setting. In particular,

$$\begin{aligned} \Phi_{elas} \equiv \frac{2\mu}{m^2}\left[\left(\frac{\lambda_I}{\lambda_{II}}\right)^{\frac{m}{2}} + \left(\frac{\lambda_{II}}{\lambda_I}\right)^{\frac{m}{2}} - 2\right] + \frac{\kappa}{2}(\lambda_1\lambda_{II} - 1)^2 - \\ - \bar{\kappa}\mathcal{H}(J_c - \lambda_I\lambda_{II})\ln(\lambda_I\lambda_{II} + 1 - J_c) \end{aligned} \tag{16}$$

where μ is the shear modulus, m a material constant, λ_I and λ_{II} are the principal stretches, κ is the in-plane bulk modulus, while $\bar{k}$ and J_c are material parameters introduced to account for the penalty associated with cellular configurations with an areal stretch $\lambda_I\lambda_{II}$ below a threshold J_c. For a complete derivation and motivation of Eq. (16), we refer the reader to [1].

The specific Helmholtz free-energy of cell adhesions f_{adh}

Cell adhesions on the substrate were approximated as linear springs, and it was assumed that springs associated with non-adhesive parts of the substrate have a very low stiffness k_n compared to the stiffness k_a of springs on adhesive areas ($k_n \ll k_a$). From this it follows that

$$f_{adh} = \begin{cases} \frac{(F(\boldsymbol{x}^{(j)}))^2}{2k_a}, & \text{if } \boldsymbol{x}^{(j)} \text{ is adhesive,} \\ \\ \frac{(F(\boldsymbol{x}^{(j)}))^2}{2k_n}, & \text{if } \boldsymbol{x}^{(j)} \text{ is non-adhesive,} \end{cases} \tag{17}$$

where $F(\boldsymbol{x}^{(j)})$ is the magnitude of the force (in material point $\boldsymbol{x}^{(j)}$) that the linear spring has to exert to equilibrate the residual forces resulting from the interaction between stress fibers and cell passive components. From $k_n \ll k_a$ it follows $\frac{(F(\boldsymbol{x}^{(j)}))^2}{2k_n} \gg \frac{(F(\boldsymbol{x}^{(j)}))^2}{2k_a}$; therefore, the approach efficiently models the preference of cells to form and mature adhesions only on adhesive lines (Eqs. (1)-(17)).

The magnitude of the force of cellular adhesions

In the present study, we assumed that cellular adhesions exert forces of a magnitude equal to the forces necessary to mechanically equilibrate the active and passive forces of the cell. This translates to assume that, given the total Cauchy stress Σ_{ij} as a summation between the stress fiber stress, the stress from cellular adhesions, and the passive stress of the other passive cellular components, we have

$$0 = \Sigma_{ij} = \sigma_{ij}^{sf} + \sigma_{ij}^{p} + \sigma_{ij}^{adh}, \tag{18}$$

with σ_{ij}^{sf} stress fiber stress, σ_{ij}^{adh} stress due to cellular adhesions, and σ_{ij}^{p} stress from other passive cellular components. It follows that, to quantify the magnitude of the force of cellular adhesions, computing σ_{ij}^{sf} and σ_{ij}^{p} is sufficient.

In agreement with [1], the stress fiber stress was computed as

$$\begin{bmatrix} \sigma_{11}^{sf} & \sigma_{12}^{sf} \\ \sigma_{12}^{sf} & \sigma_{22}^{sf} \end{bmatrix} = \frac{\mathcal{F}_0 \sigma_{max}}{2} \int_{-\pi/2}^{\pi/2} \hat{\eta}[1 + \varepsilon_{nom}(\phi)] \begin{bmatrix} 2\cos^2\phi^* & \sin 2\phi^* \\ \sin 2\phi^* & 2\sin^2\phi^* \end{bmatrix} d\phi, \tag{19}$$

with $\mathcal{F}_0 \equiv n_s(A_0 l_0)\rho_0$, while ϕ^* is the angle with respect to x_1, in the deformed configuration, that stress fibers originally oriented at an angle ϕ have in the deformed configuration. Note that Eq. (19) was determined with the assumption the cell is incompressible. This assumption, leads also to the determination of the Cauchy stress of the passive components of the cell:

$$\sigma_{ij}^{p} p_j^{(k)} = \sigma_k^{p} p_i^{(k)} \tag{20}$$

where $\sigma_k^p \equiv \lambda_k \frac{\partial \Phi_{elas}}{\partial \lambda_k}$ is the principal passive stress, with associated $p_i^{(k)}$ and $p_j^{(k)}$ unit vectors indicating the principal directions ($k = I, II$).

Numerical method for computing the Gibbs free-energy of a configuration

The cell in the undeformed configuration was discretized with three-noded triangular elements of size $R_0/10$. Different configurations (j) were then obtained by varying the displacements $\boldsymbol{u}^{(j)}$, which uniquely identify the strains in the cell. From these values, by considering the chemical equilibrium in the cell body, f_{cyto} and Φ_{elas} can be computed, as well as the passive stress within the cell and the stress fiber stress. Given these passive and active stress values, the residual traction forces $T(\boldsymbol{x}^{(j)})$ can be identified at each node, together with the consequential values of $F(\boldsymbol{x}^{(j)}) = -T(\boldsymbol{x}^{(j)})$. From

these forces, the Helmholtz free-energy f_{adh} (Eq. (17)) and the total Gibbs free-energy (Eqs. (1) and (4)) can be computed.

For the calculation of the statistics of cell shapes on a substrate (Eq. (17)), the values of $\bar{G}$ and ζ needs to be determined. First, the homeostatic energy $\bar{G}$ of a free-standing cell was computed. Only one equilibrium configuration is possible for a free-standing cell: the cell needs to be traction free and in chemical equilibrium without the contribution of the adhesions with the substrate. The total Gibbs free-energy can be computed for this unique configuration: $\bar{G} = \sum_j P_{eq}^{(j)} G^{(j)} = P_{eq}^1 G^1 = G^1$, where the index 1 refers to the unique configuration of the free-standing cell (which has $P_{eq}^1 = 1$). An iterative method was pursued to calculate ζ. Given a hypothesized value ζ_0, the corresponding average Gibbs free-energy $G_0 = \frac{1}{Z} \sum_j G^{(j)} \exp\left[-\zeta_0 G^{(j)}\right]$ was approximated with the Metropolis algorithm [4]. If $G_0 = \bar{G}$ (with a maximum absolute error equal to $2\%\bar{G}$), ζ_0 was accepted; otherwise, the procedure was repeated with a new value ζ_1. For the random walks among the possible configurations required for the Metropolis algorithm, M points were chosen among the material points of the cell. Hypothetical and random deformations $U_L^{(j)}$ (with $L = 1, âĂę, M$) were assigned to these points. The actual deformations $\boldsymbol{u}^{(j)}$ of the entire cell were generated via Non-Uniform Rational B-Splines [5], by using the values $U_L^{(j)}$ as weights. A perturbation of a specific configuration was then performed by varying the value of one of the weights $U_L^{(j)}$.

Material parameters for myofibroblasts

The material parameters were chosen similar to Buskermolen et al. [6], who simulated myofibroblasts with the same statistical framework, omitting the specific Helmholtz free-energy associated with cell adhesions and considering the contribution of the cell nucleus. The parameter differences between the two studies derive from these dissimilarities. The parameters for the cell adhesions were chosen by considering the assumption that $k_n \ll k_a$. A list of the material parameters is given in Table S1.

Orientation, aspect ratio, and area of a configuration

For each configuration (j), as in experiments, the cellular orientation and aspect ratio were obtained by fitting the outline of the cell with the best-fitting ellipse (in a least square sense), calculated using a least-square algorithm. The area of the cell was calculated by summing the areas of the deformed elements composing the cell in the deformed configuration (j).

Scaling of the computational results

We first performed computational simulations of myofibroblasts cultured on substrates with alternating adhesive and non-adhesive lines equal in widths, where the size of these widths is normalized to the diameter of the undeformed cell. In what follows, we describe the procedure to find the scaled version of these widths.

We first simulated the experiments for a large range of different line widths, spanning from lines that were small enough to enable the cell to spread on multiple lines, to lines wide enough to accommodate the cell on a single line. The computational results were presented with boxplots, with each boxplot corresponding to a different width of adhesive and non-adhesive lines. The value of the line widths is reported on the horizontal axis normalized to the diameter of the undeformed cell. Hereafter, we indicate this normalized value with l_w.

The simulations predicted that cells generally tend to co-align with adhesive lines, with the variability of alignment dependent on l_w. In particular, the alignment increased with l_w when $l_w \leq 0.3$ and decreased with l_w for $l_w \geq 0.7$. Interestingly, l_w values between 0.4 and 0.6 corresponded to a

change of trend, with alignment increasing from 0.3 to 0.5 and again decreasing for l_w from 0.5 to 0.7.

To compare the computational results with the experiments, the normalized line widths l_w have to be scaled with experimental measures. In our experiments, 20 μm seems a characteristic size for myofibroblasts, corresponding to the smallest line width where cells can spread on single lines (Figure 1 of the main text). Moreover, cells on these lines exhibited the maximum degree of alignment. Therefore, the normalized line widths were scaled by identifying the value of l_w that corresponds to 20 μm. For brevity, we indicate this value with l_w^{ref}. To determine l_w^{ref} we observe that, in the computational simulations, cells start spreading on single lines when $l_w \geq 0.6$ (Figure S4C). In addition, cells exhibit very high degrees of alignment for values of l_w in between 0.6 and 0.7. Consequently, given the similarities with the experimental results for 20 μm lines, we can assume that $0.6 < l_w^{ref} \leq 0.7$. To make the scaling more specific, another requirement for l_w^{ref} can be deduced by observing the experimental results of cells on large lines (such as 100 μm and 150 μm); cells on such lines still exhibit a relatively high parallel alignment. However, in the computational simulations, large values of l_w correspond to decreased alignment. Therefore, large experimental measures of line widths should correspond to relatively small l_w, which translates to the requirement that l_w^{ref} should be relatively small. Considering these two requirements, as a first approximation, we assumed that 20 μm corresponds to $l_w^{ref} = 0.625$ (such that $l_w = 1$ becomes equivalent to 32 μm). The computational simulations were then repeated for values of l_w corresponding with the other line widths analyzed in the experiments. The results of these computational simulations are reported in Figure 5 of the main text. In Figure **??**, morphologies of representative cells from the simulations and experiments are compared. In general, the cellular shapes obtained with the computational simulations are similar to the ones that were experimentally observed.

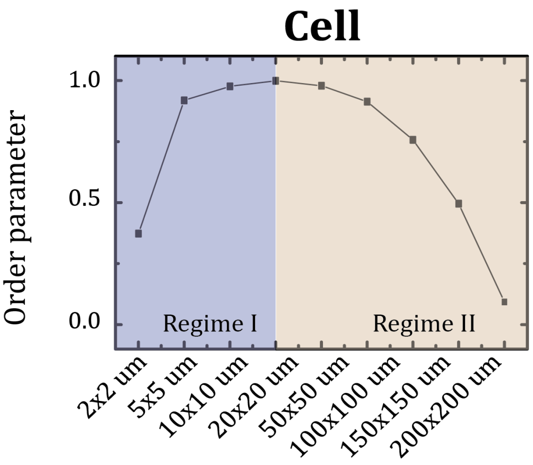

Figure S1. Measurements of the cell orientation order parameter Θ versus the line dimensions (width $\times$ spacing μm). The values of Θ range from 0–1, representing random alignment ($\Theta = 0$) for 2×2 μm lines and for increasing width perfect alignment ($\Theta = 1$). For $w > 20$ μm, Θ decreases for increasing line widths. This order parameter clearly shows the two different observed regimes in cellular alignment transitioning at a line width $w = 20$ μm. The results are expressed as the mean and are from three independent experiments. At least 60 cells were considered per condition.

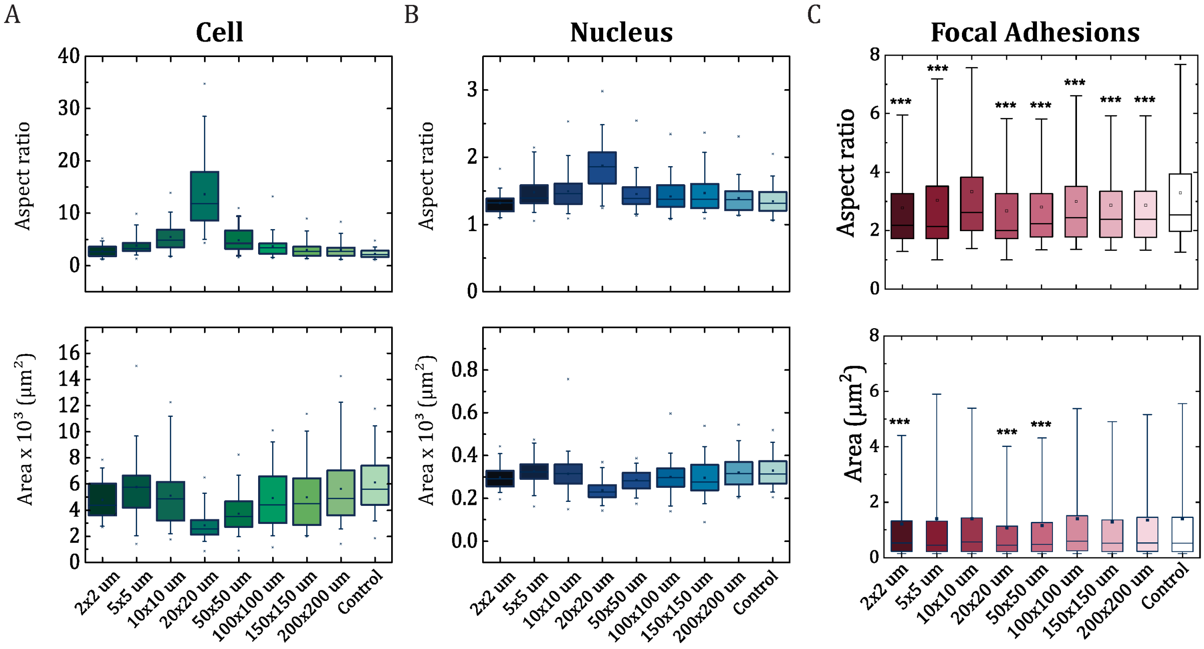

Figure S2. Distributions of area and aspect ratio for A) cells, B) nuclei, and C) FAs determined for myofibroblasts on fibronectin lines of various dimensions (width × spacing μm). The boxes of the boxplots represent the quartiles of the distributions with the whiskers indicating the outliers in the experiments and the 5th and 95th percentiles of the distributions. C) The data reported are results from three independent experiments, at least 60 (cells and nuclei) or 20 cells (FAs) were considered per condition. ***: $p < 0.001$ with respect to control.

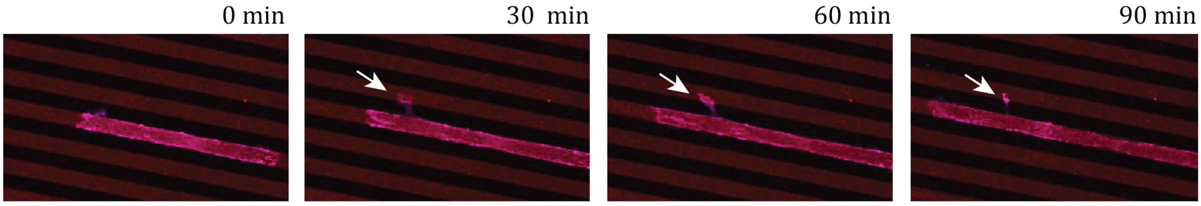

Figure S3. Snapshots from a movie of a spreading myofibroblast on 20×20 μm lines of fibronectin (red). The cell is stained for the FAs (magenta) and forms adhesions primarily on the periphery of the cell on the edges of the lines. The cell can be seem to form a protrusion perpendicular to the line bridging the non adhesive line as indicated by the white arrow. However, this protrusion is not stable and retracts.

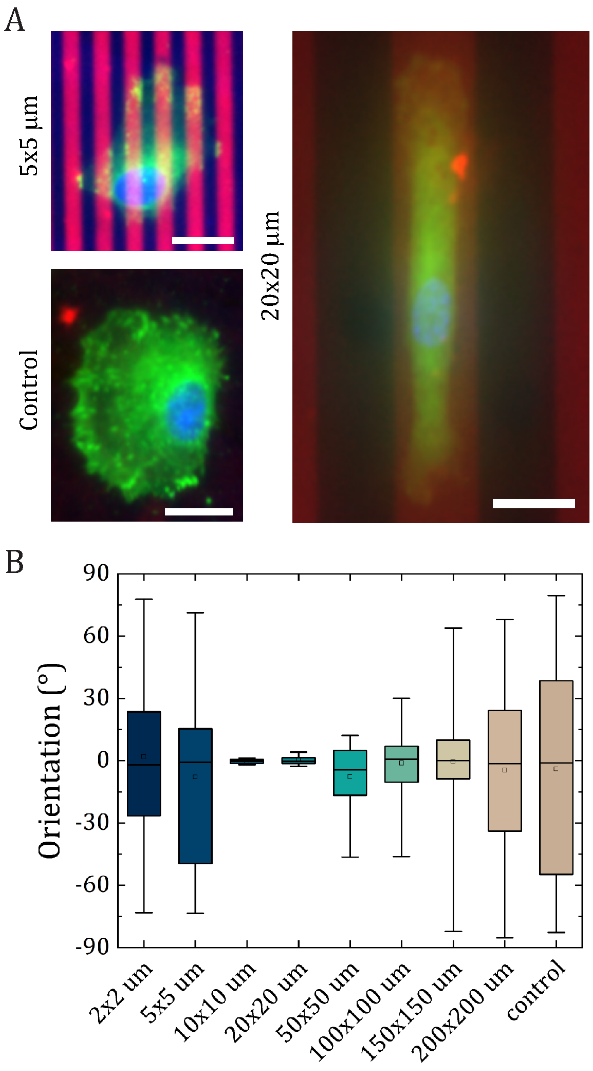

Figure S4. Substrate-pattern-guided contact guidance of cardiomyocyte progenitor cells (CMPCs). (A) Representative images of CMPCs on parallel fibronectin lines ($w \times s$ μm) stained for fibronectin (red), FAs (green), and nucleus (blue). Scale bar = 20 μm. (B) Box-and-whisker plot of the cell orientation distributions. At least 20 cells were analyzed per condition.

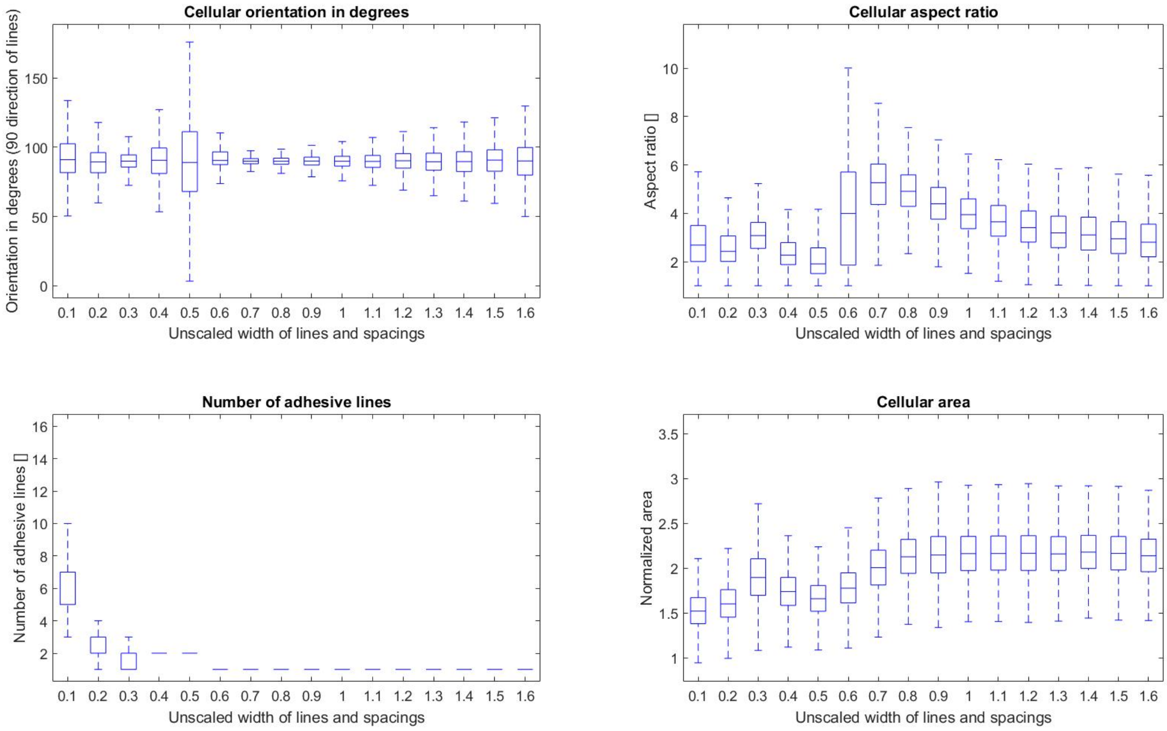

Figure S5. Overview of the results of the simulation of cells on substrates with alternating adhesive and non-adhesive lines of different line widths. The values of these widths are normalized with respect to the diameter of the undeformed cell. For simplicity, the outliers were not reported in the graphs.

Table S1. Parameter set for computational simulation of myofibroblasts on substrates with alternating adhesive and non-adhesive lines

Parameter	Value	Brief description
T	310 K	Thermodynamic temperature
μ	1.67 kPa	In-plane shear modulus of the cellular passive components
κ	35 kPa	In-plane bulk modulus of the cellular passive components
m	6	Material constant modulating the nonlinearity of the deviatoric elastic response of the cellular passive components
$\bar{k}$	1 GPa	Parameter modulating the elastic penalty as a result of a large reduction of cell area
J_c	0.6	Penalty parameter associated with a large reduction of cell area
σ_{max}	240 kPa	Maximum stress-fibre contractile stress
ρ_0	$3 \cdot 10^6\ \mu\text{m}^{-3}$	Density of stress-fibre proteins
$\mathcal{F}_0$	0.032	Stress-fibre protein volume fraction
$\tilde{\varepsilon}_{nom}^{ss}$	0.35	Steady state stress-fibre functional unit strain
$\mu_{b0} - \mu_u$	$1kT$	Difference between bound and unbound stress-fibre potential
Ω	$10^{-7.1}\ \mu\text{m}^3$	Volume of the reference number of functional units that are within a stress-fibre in the undeformed representative volume element
$\hat{\eta}_{max}$	1	Concentration of bound stress-fibre proteins
b_0/R_0	0.2	Ratio between the thickness and radius of an undeformed cell
$k_n/(\mu R_0)$	$1.061 \cdot 10^2$	Nondimensional representation of the stiffness of linear springs approximating focal adhesions on non-adhesive lines
k_a/k_n	10^2	Ratio between the stiffness of linear springs approximating focal adhesions on adhesive areas and the value for non-adhesive areas

Supplemental References

[1] Shishvan SS, Vigliotti A, Deshpande VS. The homeostatic ensemble for cells. Biomechanics and Modeling in Mechanobiology. 2018;17(6):1631–1662.

[2] Vigliotti A, Ronan W, Baaijens FPT, Deshpande VS. A thermodynamically motivated model for stress-fiber reorganization. Biomechanics and Modeling in Mechanobiology. 2016;15(4):761–789.

[3] Ogden RW. Large Deformation Isotropic Elasticity: On the Correlation of Theory and Experiment for Compressible Rubberlike Solids. Proceedings of the Royal Society A: Mathematical, Physical and Engineering Sciences. 1972;328(1575):567–583.

[4] Metropolis N, Rosenbluth AW, Rosenbluth MN, Teller AH, Teller E. Equation of State Calculations by Fast Computing Machines. The Journal of Chemical Physics. 1953;21(6):1087–1092.

[5] Piegl L, Tiller W. Symbolic operators for NURBS. CAD Computer Aided Design. 1997;29(5):361–368.

[6] Buskermolen ABC, Suresh H, Shishvan S, Vigliotti A, DeSimone A, Kurniawan NA, et al. Entropic forces drive cellular contact guidance. Biophysical Journal. 2019;116(10):1994–2008.
