# Supplementary material for: Cellular Contact Guidance Emerges from Gap Avoidance
Source: Cell Rep Phys Sci. 2020 May 20;1(5):100055. doi: 10.1016/j.xcrp.2020.100055 (PMC7357833; doi:10.1016/j.xcrp.2020.100055)
Supplement: Document S2. Article plus Supplemental Information [file mmc3.pdf]

Cell Reports
Physical Science

CellPress
OPEN ACCESS

Article

Cellular Contact Guidance Emerges from Gap Avoidance

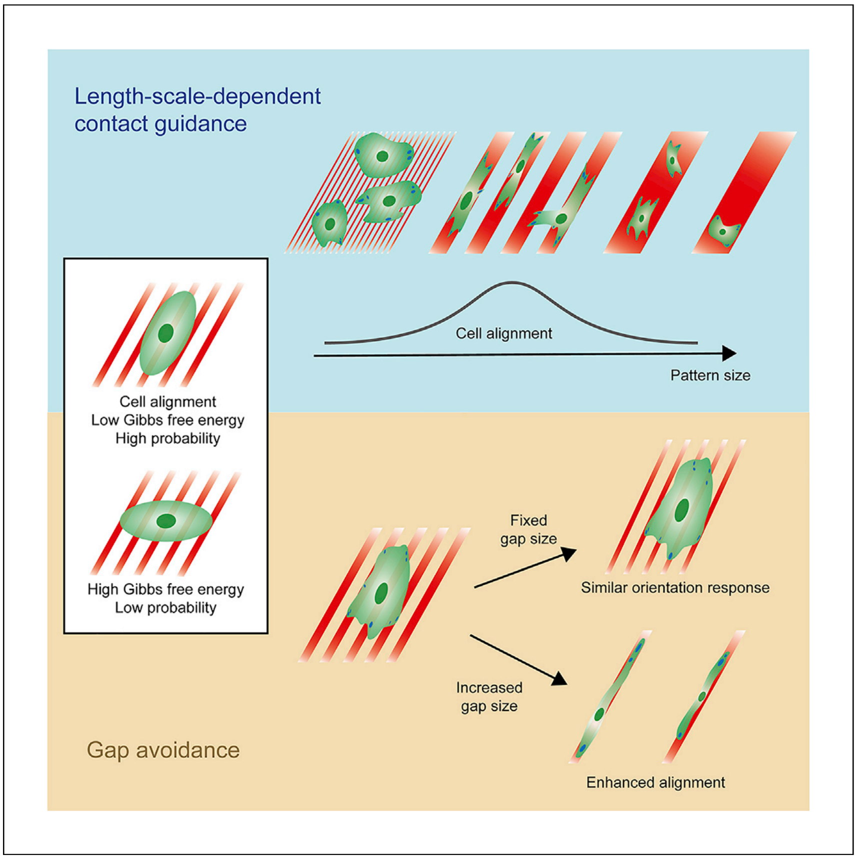

Buskermolen et al. report that cells align to substrate micropatterns in a highly length-scale-dependent manner. This alignment emerges from energetically favorable cell elongation and "gap avoidance": minimization of cellular adhesions on non-adhesive gaps. This finding points to biophysically driven mechanisms underlying cellular contact guidance without necessarily invoking specific molecular pathways.

Antonetta B.C. Buskermolen, Tommaso Ristori, Dylan Mostert, ..., Nicholas A. Kurniawan, Vikram S. Deshpande, Carlijn V.C. Bouten

n.a.kurniawan@tue.nl (N.A.K.)
c.v.c.bouten@tue.nl (C.V.C.B.)

HIGHLIGHTS

Cells exhibit an optimum alignment (contact guidance) to substrate micropatterns

Contact guidance does not require constrained alignment of focal adhesions

Statistical mechanics model reveals the energetic role of non-adhesive gaps

The non-adhesive gap size determines the degree of cell alignment

Buskermolen et al., Cell Reports Physical Science *1*, 100055
May 20, 2020 © 2020 The Author(s).
https://doi.org/10.1016/j.xcrp.2020.100055

Cell Reports
Physical Science

CellPress
OPEN ACCESS

Article

Cellular Contact Guidance Emerges from Gap Avoidance

Antonetta B.C. Buskermolen,[1,2,5] Tommaso Ristori,[1,2,5] Dylan Mostert,[1,2] Mark C. van Turnhout,[1] Siamak S. Shishvan,[3,4] Sandra Loerakker,[1,2] Nicholas A. Kurniawan,[1,2,6,*] Vikram S. Deshpande,[4] and Carlijn V.C. Bouten[1,2,*]

SUMMARY

In the presence of anisotropic biochemical or topographical patterns, cells tend to align in the direction of these cues—a widely reported phenomenon known as "contact guidance." To investigate the origins of contact guidance, here, we created substrates micropatterned with parallel lines of fibronectin with dimensions spanning multiple orders of magnitude. Quantitative morphometric analysis of our experimental data reveals two regimes of contact guidance governed by the length scale of the cues that cannot be explained by enforced alignment of focal adhesions. Adopting computational simulations of cell remodeling on inhomogeneous substrates based on a statistical mechanics framework for living cells, we show that contact guidance emerges from anisotropic cell shape fluctuation and "gap avoidance," i.e., the energetic penalty of cell adhesions on non-adhesive gaps. Our findings therefore point to general biophysical mechanisms underlying cellular contact guidance, without the necessity of invoking specific molecular pathways.

INTRODUCTION

Cell organization plays a crucial role in the micro-architecture of tissues, dictating their biological and mechanical functioning.[1] *In vivo*, cells are embedded in the extracellular matrix (ECM), which generally comprises a network of organized micrometer-scale fibers that provide cells with anisotropic geometrical cues.[2–4] Cells typically align in the direction of the cues—a phenomenon known as cellular contact guidance. Contact guidance has been widely observed for several decades[5,6] and has been shown to affect various downstream cell behaviors, including survival, motility, and differentiation.[7–9] Uncovering the underlying mechanisms is critical for a better understanding of tissue and organ morphogenesis and regeneration.[10]

Numerous studies have demonstrated and examined contact guidance effects, typically by culturing cells on microfabricated anisotropic substrates consisting of microgrooves, adhesive lines, or fibers (see Tamiello et al.[11] for an extensive review). Collectively, these studies have shown that contact guidance intriguingly occurs on substrate patterns of a wide range of sizes. For example, it was shown that contact guidance at length scales smaller than focal adhesions (FAs) arises as a result of constrained FA alignment and maturation in the direction of anisotropy.[9,12] More recently, we showed that cells constrained on single lines of fibronectin align in the line direction not only when the line width is smaller than the cell body, thereby

[1]Department of Biomedical Engineering, Eindhoven University of Technology, Eindhoven, the Netherlands
[2]Institute for Complex Molecular Systems, Eindhoven University of Technology, Eindhoven, the Netherlands
[3]Department of Structural Engineering, University of Tabriz, Tabriz, Iran
[4]Department of Mechanical Engineering, University of Cambridge, Cambridge, UK
[5]These authors contributed equally
[6]Lead Contact
*Correspondence: n.a.kurniawan@tue.nl (N.A.K.), c.v.c.bouten@tue.nl (C.V.C.B.)
https://doi.org/10.1016/j.xcrp.2020.100055

This is an open access article under the CC BY license (http://creativecommons.org/licenses/by/4.0/).

enforcing cell alignment, but also on wider lines, as a result of entropy-mediated mechanisms.[13] However, contact guidance due to cues of intermediate-length scales (μm to hundreds of μm) has remained unexplored.

In the present study, we investigated the emergence of cellular contact guidance resulting from anisotropic cues at these intermediate length scales, from the typical size of FAs to the typical cell length. We found that myofibroblasts exhibited two regimes of cellular alignment: one at small length scales of the anisotropic cue, where cell alignment is induced by multiple patterns, and one at large-length scales, as a result of spatial confinement of the whole cells. Interestingly, these alignments occurred in the absence of spatially constrained FA alignment, suggesting that contact guidance can result from an alternative mechanism. To understand this, we extended a recently established statistical mechanics framework for living cells,[14] enabling the simulation of cells on substrates with a heterogeneous distribution of ligands. Quantitative comparison between our experimental and computational data indicates that contact guidance arises from the minimization of cellular adhesions on non-adhesive regions and that non-adhesive gaps play a decisive role for cell alignment.

RESULTS

Cellular Alignment and Intracellular Organization Are Controlled by Substrate Anisotropy at Micron- to Cell-Size Scale

To mimic the fibrillar nature of the ECM and examine the effects of substrate anisotropy on cellular alignment, we created micropatterns consisting of parallel lines of fibronectin with defined widths and inter-line spacings using microcontact printing and seeded human myofibroblasts on these microcontact printed substrates. This experimental approach allowed us to systematically probe cellular contact-guidance response in a purely planar setup, without the need to consider the influence of the third dimension as in studies employing microfabricated grooves and ridges. The line width w and inter-line spacing s ranged from 2 to 200 μm and were initially chosen to be equal (i.e., $w = s$) to maintain a constant cell-substrate contact area. The lower limit of $w = 2$ μm was motivated by the size of typical mature FAs,[15,16] whereas the upper limit of $w = 200$ μm is large enough to allow cell spreading on one single line (cf. individual myofibroblast length on fibronectin-coated substrate; Figure 1C).

Images obtained 24 h after seeding showed that cell morphology and orientation are strongly influenced by the width of the lines (Figure 1A). On the thinnest lines ($w = 2$ μm), there was only a weak cell-alignment effect due to the micropatterned lines. When w was increased up to 20 μm, cells increasingly elongated and aligned parallel to the lines. The trend inverted when w was further increased up to 200 μm. We quantified the changes in cell shape and orientation for more than 600 cells on the substrates using an automated morphometric analysis of the immunofluorescence images.[17] Briefly, we fitted an ellipse to the cell outline and defined the orientation angle θ as the angle between the major axis of the best-fitted ellipse and direction of the lines (Figure 1B). The analysis revealed that, with increasing w, the distribution of θ around 0° strongly narrowed up to $w = 20$ μm, indicating enhanced cell alignment, and then broadened back toward an isotropic distribution, resembling that on homogeneous substrates (control; Figure 1D). Furthermore, we noted that $w = 20$ μm also demarcates the transition from the situation where cells adhered on more than one line to the situation where cells fitted within single lines.

CellPress
OPEN ACCESS

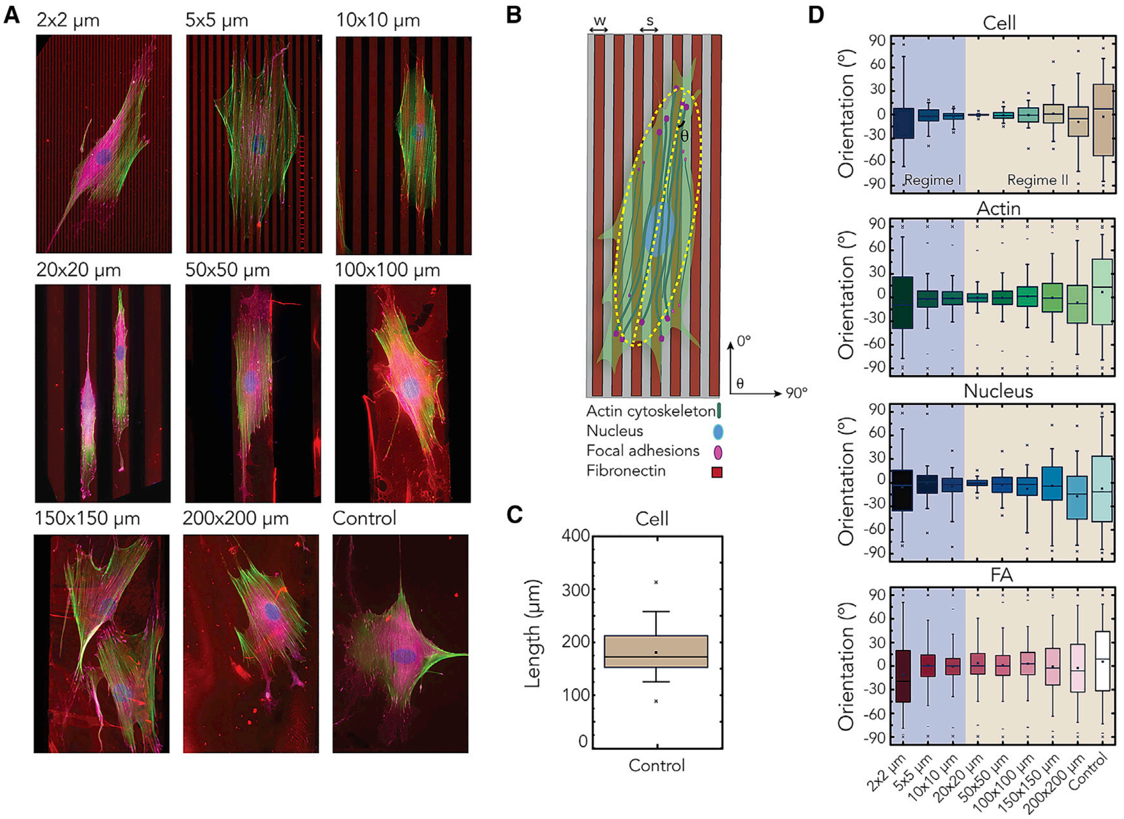

Figure 1. Two Regimes of Cellular Alignment

(A) Representative immunofluorescence images of myofibroblasts on parallel lines ($w \times s$ µm) of fibronectin (red) stained for the FAs (magenta), actin cytoskeleton (green), and nucleus (blue).

(B) Schematic diagram showing the analysis of cell orientation based on the best-fitted ellipse (dashed yellow).

(C) The length of a cell on a homogeneous substrate (control).

(D) The cell, actin fiber, nucleus, and FA orientation, where 0° represents the direction of the lines. The boxes of the boxplots represent the quartiles of the distributions, with the whiskers indicating the outliers in the experiments and the 5th and 95th percentiles of the distributions. Note that, with this data representation, the median is at 0° and the box ranges from –45° to 45° when the distribution of cell orientation is perfectly isotropic. The data reported are results from three independent samples; at least 60 cells were considered per condition.

These data demonstrate that the cellular orientation response can be divided into two regimes: regime I for $w < 20$ µm, where cell alignment was induced by multiple lines, and regime II for $w \geq 20$ µm, where cell alignment was influenced by the spatial confinement within single lines. In regime II, as we previously observed,[13] cell alignment decreased with increasing w. In regime I, cell alignment decreased with decreasing w. The two regimes were also captured by the order parameter Θ (see Equation 1 in the Experimental Procedures and Figure S1), clearly showing that the order transitions at $w = 20$ µm.

Cell alignment on the micropatterned lines was accompanied by changes in cell morphology, as quantified through aspect ratio and adhesion area (Figure S2A). The largest degree of alignment was observed when cells were most elongated. Because cell morphology is governed by the dynamics and properties of intracellular structural components, such as the nucleus and individual actin fibers,[18] we hypothesized that the two observed regimes should also reflect in the organization of these components. Indeed, the actin fibers and nuclei showed similar trends as for the cell orientations, including the two regimes of alignment (Figures 1D and

S2B). This confirms that micrometer-scale variations in the extracellular patterns can tune cell orientation as well as intracellular organization.

Contact Guidance Does Not Require Constrained Alignment of FAs

On patterns with dimensions smaller than FAs, it has been previously reported that spatially constrained alignment of FAs play a central role in contact guidance.[9] Generally, when cells adhere to substrates homogeneously coated with fibronectin, they first form small, nascent FAs (0–2 μm long), which can either disappear or develop into 2- to 6-μm-long, mature FAs.[19] Because a minimum length of 2 μm has been shown to be required for local contacts to establish adhesions[20] and the FAs of our cells can grow to lengths much larger than 2 μm (Figure S3), we reasoned that the lines with $w = 2$ μm would provide an area for FA maturation and orientation only in the direction of the lines and that FAs could have a wide distribution of orientations on 5- and 10-μm lines, where FA maturation is not spatially constrained. By examining the FA and cell orientation on these line widths, we therefore can test the requirement of FA alignment for cellular contact guidance. Indeed, Ray et al.[9] have recently suggested that relaxing spatial constraints on FA maturation and consequently FA organization diminishes cellular alignment. Surprisingly, we obtained the opposite result: on lines of $w = 2$ μm, a wide distribution of orientation angles of FAs was observed, whereas the FAs became slightly more aligned at larger line width w (Figure 1D). This trend is similar to, but weaker than, the orientation response of the cells. Therefore, our data show that, at length scales larger than FA size, increasing the adhesive area for FAs leads to the counterintuitive increase of FA and cell alignment in the direction of the lines. This suggests that contact guidance at these length scales does not arise from spatially constrained alignment of FAs, which is an underlying mechanism of contact guidance at smaller scales.[9,12]

To further confirm this observation, we investigated in more detail the morphology and organization of FAs in regime I. The analysis showed that lines of $w = 2$ μm were able to constrain FA maturation. In particular, vinculin staining showed that FAs were primarily formed on the adhesive fibronectin lines and that the distributions of the FAs were markedly influenced by the lines (Figure 2A). Specifically, for $w = 5$ and 10 μm, we observed rows of FAs lining up along the edge of the lines (Figure 2A, insets). For cells on the 2-μm-wide lines, we found small, non-aligned adhesions, in contrast to the large, non-aligned adhesions on homogeneously coated substrates of fibronectin. Moreover, FAs were significantly smaller for 2-μm lines relative to the homogeneous substrate (Figure S2C), consistent with the notion that lines of 2-μm width provided a geometrical constrain for FA maturation. Increasing the line width w resulted in more aligned, elongated FAs in the direction of the lines (Figure 2A). We further characterized the size and shape of individual FAs by determining their length and aspect ratio, respectively. The length and aspect ratio of FAs on 2-μm lines were significantly smaller compared to those on homogeneous substrates, whereas the long axes of FAs on 5- and 10-μm lines were equal to those on the homogeneous substrates (Figures 2C and 2D). The aspect ratio of FAs on 5-μm lines was smaller compared to FAs on 10-μm lines, meaning that FAs on 5-μm lines are long and wide, although FAs on 10-μm lines are thinner.

Given these differences in the FA morphology, we classified the FAs into three categories: “round” FAs are those with aspect ratio smaller than 1.6; “non-aligned” FAs are non-round FAs whose orientation angle θ is larger than 20°; and “aligned” FAs are non-round FAs with θ smaller than 20°.[17] We found that the fraction of aligned FAs increases and the fraction of non-aligned FAs decreases for increasing line widths (Figure 2E). Interestingly, for 2-μm lines, only a small amount of FAs aligned in direction

CellPress
OPEN ACCESS

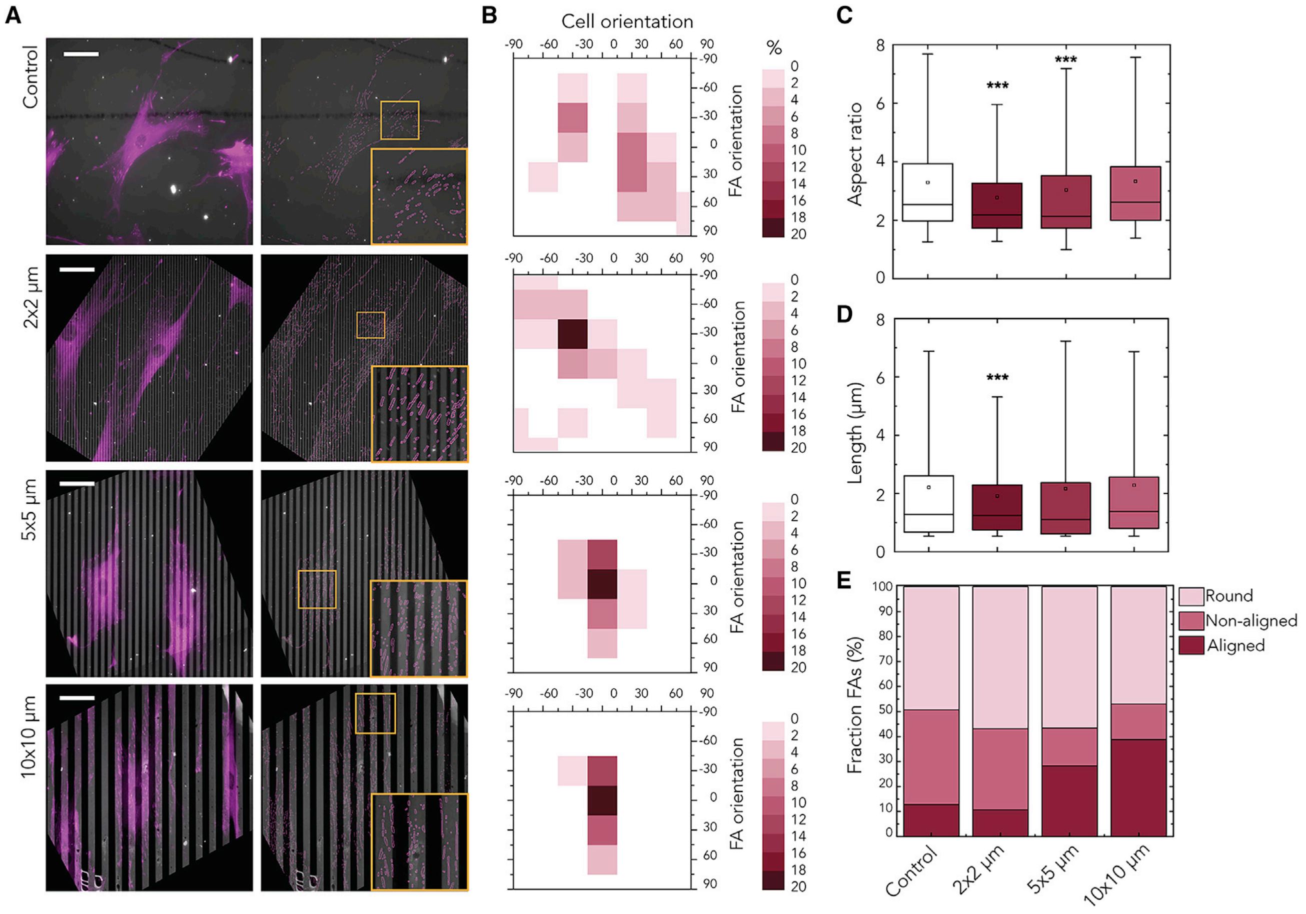

Figure 2. Constrained FAs Do Not Guide Cellular Alignment

(A) Representative immunofluorescence images of the FAs (magenta) of myofibroblasts on parallel lines ($w \times s$ μm) of fibronectin (gray). The detected outlines are shown in magenta, and the orange rectangles marked areas show zoom-in images of the FAs. Scale bars: 50 μm.

(B) 2D density plots for each line width showing the correlation between cell and FA orientation, where 0° represents the direction of the lines. The color scales show the percentage of FAs in a specific orientation with more intense pink corresponding to more FAs in this direction. These plots indicate that cellular alignment was not fully governed by constrained FAs.

(C–E) Quantitative analysis of the aspect ratio (C), length of long axis (D), and percentage of FAs that are round, non-aligned, or aligned (E). The boxes of the boxplots in (C) and (D) represent the quartiles of the distributions, with the whiskers indicating the outliers in the experiments and the 5th and 95th percentiles of the distributions. The data reported are results from three independent samples, and at least 20 cells were considered per condition. ***$p < 0.001$ with respect to control.

of the lines (11%), comparable to that on a homogeneous substrate (13%). These results strongly suggest that constrained FA maturation on 2-μm lines was not enough to induce a cell alignment distribution different than for cells on homogeneous substrates.

To evaluate further whether the orientation of FAs has a role in contact guidance for linear patterns larger than FAs, we constructed 2D density plots showing the distribution of the orientations of cells and FAs. The data show that strong cell alignment (i.e., contact guidance) does not correspond to a similarly strong alignment of mature FAs. Specifically, the density plots demonstrate that the distribution of orientation angles of FAs was scattered, showing all possible orientations (Figure 2B). This was again comparable to the orientations of FAs on homogeneous substrates (control), despite the increasing cell alignment for increasing *w*. Together, these results indicate that, at the investigated length scales, a mechanism independent of FA alignment determines contact guidance of myofibroblasts.

Cells Minimize the Number of Non-adhesive Gaps to Bridge

In an earlier study, we demonstrated that contact guidance for cells constrained on single lines (i.e., in regime II) emerges from an entropy-mediated mechanism, driven by the non-thermal shape fluctuations of cells.[13] We therefore asked whether such a mechanism may in fact be responsible for contact guidance over both regimes that we observed in the present study. To test this, we extended the statistical homeostatic mechanics framework developed by Shishvan et al.[14] to model cell behavior. This framework was adopted because it captures an essential statistical feature of cell behavior: cells exhibit large fluctuations in terms of single-cell geometrical parameters (e.g., area, orientation, and aspect ratio), with clear trends that arise only when the statistics of these observables are compared for different experimental conditions. This is evident in our experimental observations (e.g., Figure 1D). The main hypothesis is that cells continually change their shape, orientation, and intracellular components by (1) minimizing their free energy over the short-term period (seconds), while (2) maintaining an average homeostatic free energy over the long-term period (minutes). Recent reports strongly suggest that these hypotheses hold for different cell types. For example, Suresh et al.[21] adopted an analogous framework to predict stem cell differentiation.

Shishvan et al.[14] computed the free energy associated with each cell configuration on a substrate, f, by accounting for the energy contribution of the stress fiber cytoskeleton f_{cyto} and the passive elasticity of other cellular components Φ_{elas}. By simulating a large number of possible cell configurations, each with its free energies, previous studies have shown that this modeling framework can give critical insights into the ensemble behavior of adherent cells cultured on homogeneous substrates of varying stiffnesses[14] or on single adhesive lines of different width.[13] To model cell behavior on substrates with heterogeneous distribution of ligands as in the micropatterned substrates used in the present study, we extended this framework by considering the free-energy contribution of cell adhesions f_{adh}, which are approximated as linear springs with spring constants k_n and k_a on non-adhesive and adhesive areas, respectively (see Experimental Procedures for a detailed description). As a consequence of these energy contributions, cells elongate up to a certain extent to minimize the contribution of both the stress fibers and passive elastic components to the cell free energy, while at the same time avoid forming adhesions on non-adhesive areas of the substrates because they are associated with high values of free energy.

A comparison between the computational and experimental results for myofibroblasts on substrates with lines of width w is shown in Figures 3 and S6. Remarkably, the simulations were able to model the two distinct regimes of cellular alignment observed experimentally (Figure 3A). Similar to the experimental observations, in regime I, cells increasingly aligned and elongated in the direction of the lines with increasing w up to a maximum at $w = 20$ μm (Figures 3A and 3B). It is worth emphasizing that these two regimes of cellular alignment emerged solely from the inclusion of f_{adh}, without adjusting any parameters for the other free-energy contributions. Furthermore, dynamic cellular protrusions were not simulated, leading to the expected underestimation of cell area compared to experimental data (Figure 3C). The simulations predicted that cells bridge a decreasing number of adhesive lines with increasing w (Figure 3D). This was in qualitative agreement with the experiments and indicated that cells thin along the direction perpendicular to the lines, thereby resulting in a reduced number of gaps they are in contact with. In regime II, cells spread on single adhesive lines (Figure 3D). At the onset of regime II (i.e.,

CellPress
OPEN ACCESS

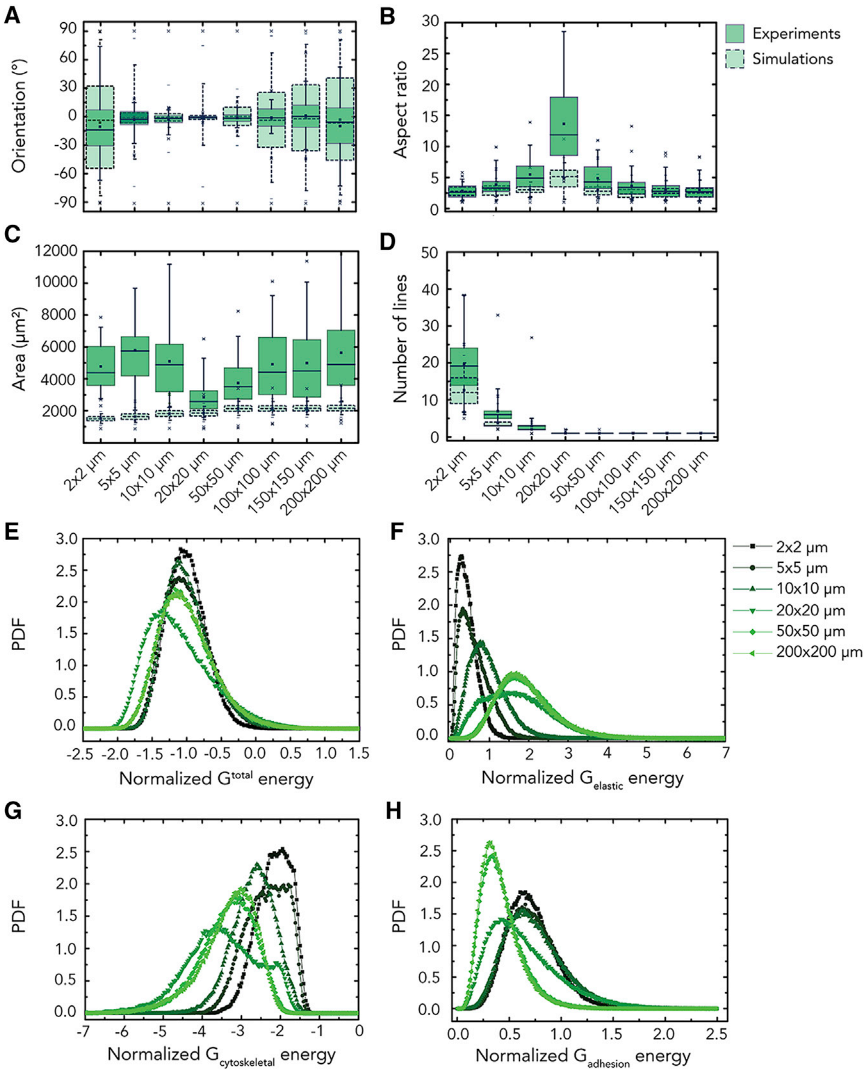

Figure 3. Modeling of Cellular Contact Guidance

(A–D) The computational (dashed green boxes) results for myofibroblasts on parallel lines of fibronectin (2–200 μm) is compared against the corresponding experimental results (solid green boxes), in terms of the distribution of the cellular orientation (A), aspect ratio (B), area (C), and number of lines touched by single cells (D). The boxes of the boxplots represent the quartiles of the distributions, with the whiskers indicating the outliers in the experiments and the 5th and 95th percentiles of the distributions.

(E–H) Probability density functions (PDFs) of the energies of myofibroblasts associated with the total Gibbs free energy (E), elasticity (F), cytoskeletal (G), and adhesion Gibbs free energy of the cell (H) are also shown. Each color represents a specific pattern of parallel lines ($w \times s$ μm). The energies are normalized by the energy of the free-standing cell. The data reported are results from three independent samples, and at least 60 cells were considered per condition.

on 20-μm-wide lines), cells fit on single lines by thinning along the perpendicular direction, while strongly elongating and orienting in the direction of the lines (Figures 3A and 3B). With increasing *w*, the perpendicular thinning and longitudinal elongation necessary for cells to fit on single lines are smaller and therefore cells have increasing freedom to vary their main orientation, consistent with our earlier study.[13] These results indicate that the hypotheses of free-energy minimization over the short timescale (seconds) and homeostasis over the long timescale (minutes) can explain

the variation of cell morphologies observed for cells on substrates with alternating (non-)adhesive lines.

Given the agreement between the model and experimental observations, we proceeded to analyze the model features that are responsible for cell alignment in the simulations. The probability density functions of the total Gibbs free energy of cells in regime I (i.e., $w < 20$ μm) exhibited smaller interquartile ranges compared to cells in regime II (Figure 3E), suggesting that adhesion to multiple lines forces cells to explore less free-energy states and morphologies. In addition, we found increasing absolute values of the elastic Gibbs free energy for increasing line widths (Figure 3F), corresponding to an increase in cell deformation, which was compensated by a corresponding decrease in the cytoskeletal Gibbs free energy (Figure 3G). The Gibbs free energy of cell adhesions was relatively large for cells on multiple lines compared to that for the other line widths (Figure 3H). This suggests that cells reduce the adhesion energy by minimizing the contact with the non-adhesive lines by (1) reducing their total spread area (Figure 3C) or by (2) thinning in the direction perpendicular to the lines, resulting in overall cell alignment (Figures 3A and 3B). Interestingly, cells on 50 μm and 200 μm show almost identical probability density functions for the cellular free energies, although a much higher alignment was observed for the 50-μm-wide lines compared to the 200-μm ones (Figures 3A and 3E). Thus, it appears that, in regime II, a relatively high degree of alignment can be achieved without substantially perturbing the cell free energy. Notably, cells on 20-μm-wide lines have energy profiles that are remarkably different from all the others; the total and cytoskeletal Gibbs free energies are much lower, with wider interquartile ranges compared to the other line widths (Figures 3E and 3G). The Gibbs free energy of cells was affected by forcing cells to spread along single lines that are small compared to the size of the cell, inducing a very high degree of alignment. Taken together, the statistical thermodynamics framework suggests that contact guidance results from the tendency of cells to spread to minimize their cytoskeleton free energy while avoiding the formation of cell adhesions on non-adhesive lines. In other words, contact guidance may emerge from the maximization of actomyosin polymerization into stress fibers and minimization of FA formation on non-adhesive areas.

The Non-adhesive Gap Size Determines the Degree of Cell Alignment

The insights from the model imply that cellular contact guidance may in fact be induced by the cells' reluctance to bridge across non-adhesive gaps. The gaps between adhesive patterns have been previously suggested to act as a barrier to the formation of stable adhesions and protrusions.[22] Live-cell imaging of myofibroblasts stained for the actin cytoskeleton (silicon rhodamine [SiR] actin) and FAs (GFP-Talin) for 24 h indicated that, while spreading, cells formed numerous protrusions and adhesions at both ends of the cell body, and smaller adhesions were also formed on the side of the cells, even after complete cell spreading and alignment. Moreover, cells were observed to form exploratory protrusions extending perpendicular to the lines, bridging the non-adhesive area, but these were often short lived and eventually retracted without initiating further cell spreading in that direction (see an example in Figure S3 and Video S1). As a result, over time, the cells elongated and oriented in the direction of the lines. This corroborates the notion that non-adhesive gaps between the adhesive lines hinder stable adhesions and thereby promote anisotropic cell spreading.

An implication of these experimental and computational findings is that contact guidance at length scales from the FA to the cell size is induced not by the width

CellPress
OPEN ACCESS

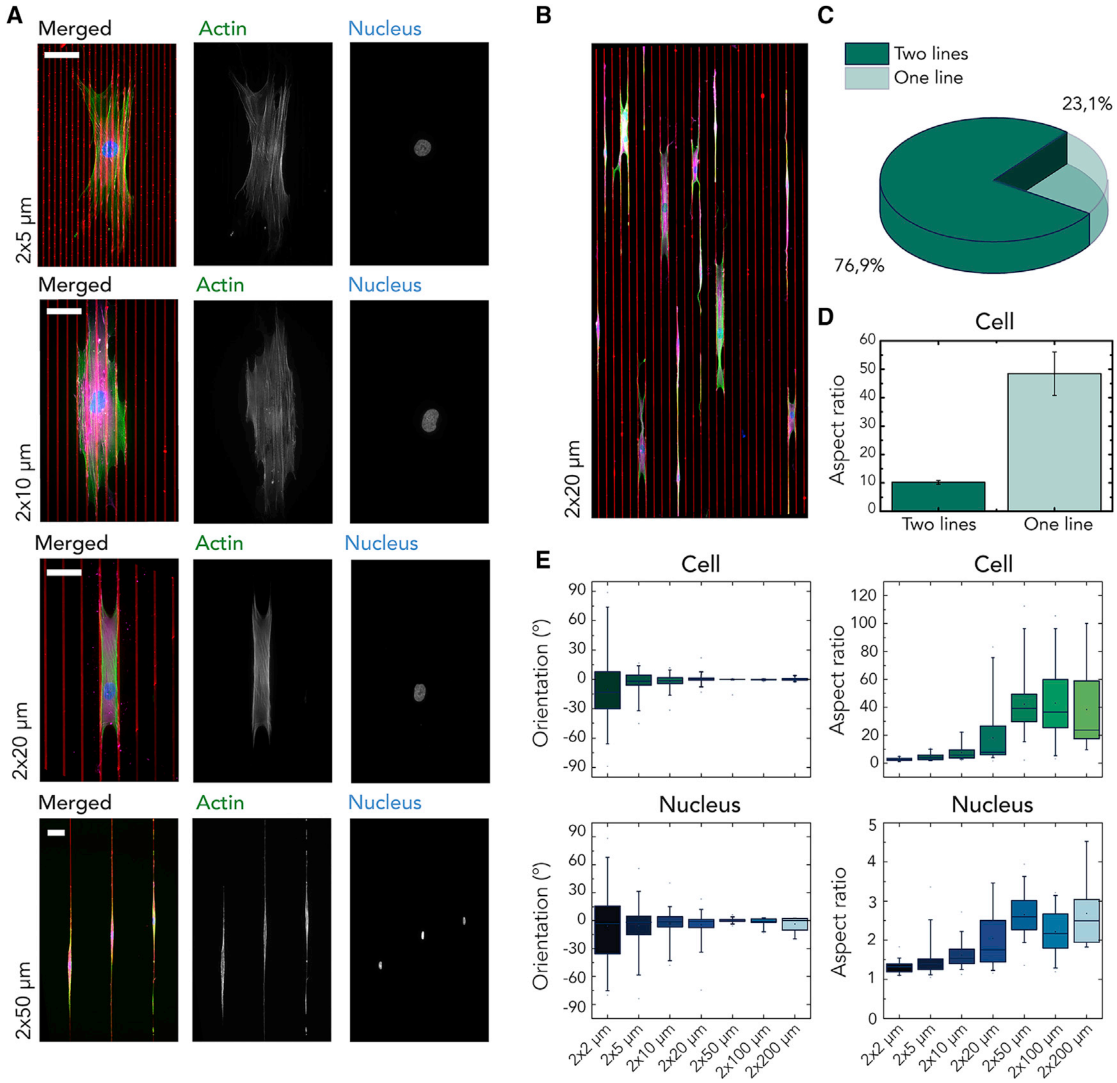

Figure 4. Influence of Inter-line Spacing on Cellular Alignment

(A) Representative immunofluorescence images of myofibroblasts on fibronectin lines of w = 2 μm (red) and varying inter-line spacings (s = 2–50 μm), stained for the actin cytoskeleton (green), nucleus (blue), and FAs (magenta). Scale bars: 50 μm.

(B) Cells on 2 × 20 μm (width × spacing) wide lines of fibronectin showing the transition from cells that bridge the non-adhesive gap to cells that do not bridge the non-adhesive gap.

(C) The probability of cells spreading on either one or two lines on 2 × 20 μm patterns.

(D) The corresponding aspect ratio of these cells. Data are represented as the mean ± standard error of the mean (SEM).

(E) Quantitative analysis of cells (top) and nuclei (bottom) demonstrates that the inter-line spacing has a clear influence on the orientation and aspect ratio. The boxes show the quartiles of the distributions, with the whiskers indicating the outliers and the 5th and 95th percentiles of the distributions of the cell and nucleus orientation and aspect ratio. The data reported are results from three independent samples; at least 60 cells were considered per condition.

of the fibronectin lines w but by the inter-line spacing s. To verify this inference, we performed a new set of experiments where we varied s in the range of 2–50 μm while keeping w constant (2 μm). In myofibroblasts spreading over substrates with 2- to 20-μm-wide spacing, actin fibers were observed running in all directions throughout the cells, often crossing several adhesive and non-adhesive areas (Figure 4A). The inter-line spacing of s = 20 μm was found to be the transition point where myofibroblasts either spread between two lines (76.9% of all cells on this pattern) or spread along single lines (23.1%; Figures 4B and 4C). These two configurations were also reflected

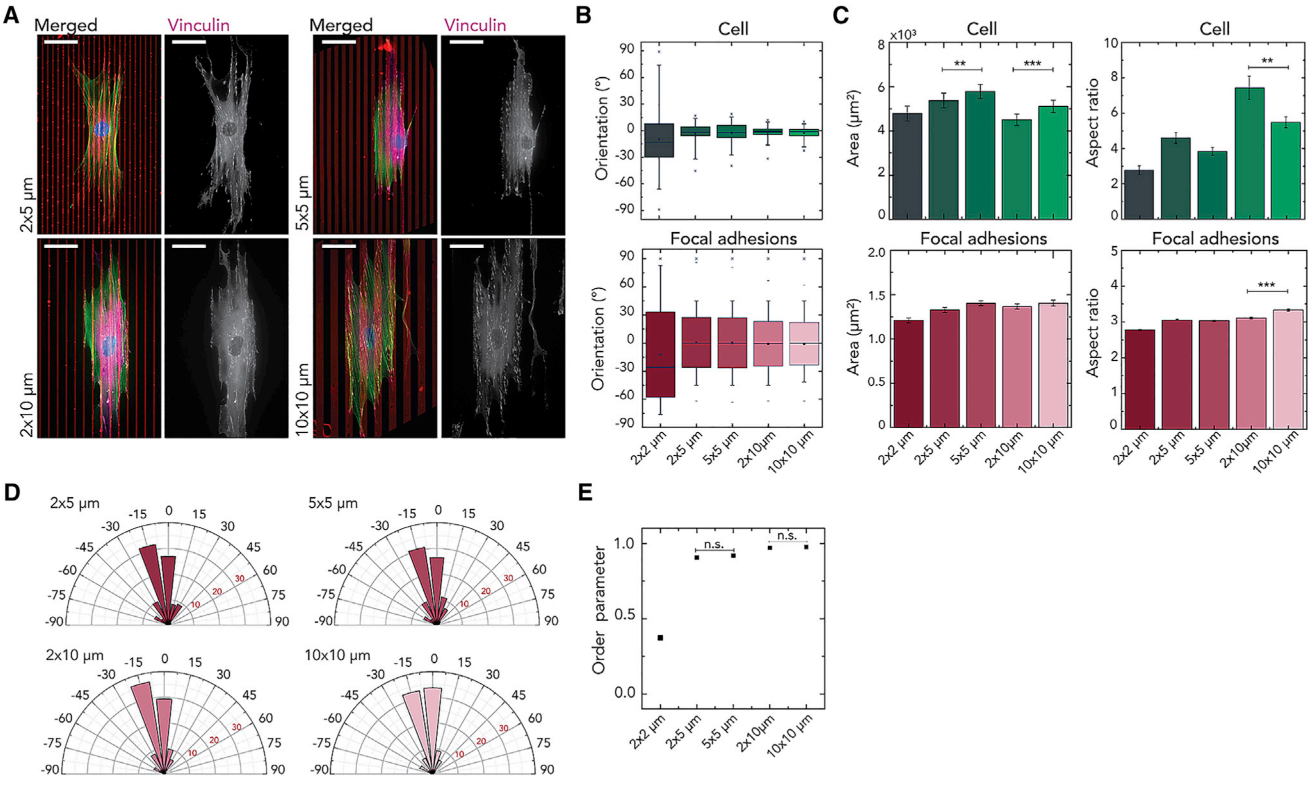

Figure 5. Influence of Line Width on the Cellular and FA Organization

(A) Representative immunofluorescence images of myofibroblasts on fibronectin lines (red) of various and inter-line spacings (w = 2, 5, and 10 µm and s = 2, 5, and 10 µm) stained for the actin cytoskeleton (green), nucleus (blue), and FAs (magenta). Scale bars: 50 µm. The width of the lines has no effect on the cell orientation, although it affects cell area and aspect ratio (B and C). The corresponding FAs are also not affected by line width (B and D).

(B) Boxplots represent the mean with 5th–95th percentile range, with the whiskers indicating the outliers of the cell and FA orientation.

(C) The mean ± SEM of the cellular and FA area and aspect ratio. *p < 0.05; **p < 0.01; ***p < 0.001. The data reported come from three independent samples, and at least 20 cells were considered per condition.

(D) The angular histogram of the FA orientations, where 0° represents the direction of the lines.

(E) Measurements of the cell orientation order parameter Θ versus the line dimensions (width × spacing µm). The values of Θ range within 0–1, corresponding to random alignment when Θ = 0 and perfect alignment when Θ = 1. The results are from three independent experiments. At least 60 cells were considered per condition.

in the cell aspect ratio, which was much higher when cells stayed within single lines (Figure 4D). When s > 20 µm, the myofibroblasts failed to reach the neighboring adhesive line and became highly elongated, resulting in a pronounced alignment in direction of the lines (Figure 4E). This was accompanied by intracellular remodeling, such as increasing organization of the actin fibers and elongation of the nucleus. Remarkably, the transition at s = 20 µm coincides exactly with the transition from regime I to regime II that we saw earlier (Figure 1D), indicating that the latter is caused by the myofibroblasts' (in)ability to bridge multiple lines separated by large gaps.

We also checked the effect of varying line widths w, but not the inter-line spacing s, by comparing patterns of 2 × 5 µm with 5 × 5 µm and 2 × 10 µm with 10 × 10 µm lines. Consistent with our hypothesis, varying the line width w alone had little effect on the orientation of cells and FAs (Figures 5B, 5D, and 5E). By contrast, inter-line spacing s has a clear effect on cell morphology, as quantified by their area and aspect ratio (Figure 5C). For increasing s, cells spread more; however, the FAs were not affected. By comparing the distribution median of the absolute

CellPress
OPEN ACCESS

value of cell angles (using the independent-samples median test), we observed that the difference between cells on substrates with different values of *w* and equal values of *s* was not statistically significant (Figure 5E). In contrast, changing values of *s* generally led to statistically different median values ($p < 0.05$ for all comparisons except for 2 × 5 μm and 2 × 10 μm, $p = 0.54$).

Taken together, our experiments combined with computational modeling support the idea that cell alignment induced by anisotropic cues larger than FAs and smaller than the typical cell length can result from the minimization of cellular adhesions on non-adhesive gaps—a phenomenon we define as "gap avoidance."

DISCUSSION

The ability of cells to align in the direction of topographical or biochemical linear patterns, i.e., contact guidance, is critical in various physiological contexts.[23–25] Previous studies on contact guidance have shown that anisotropic features created by microcontact printing can direct cell alignment.[16,26–30] Although numerous studies have already extensively characterized the ability of a variety of cells to respond to anisotropic features present in their environment, a universal mechanism underlying this phenomenon remains elusive. Particularly, although spatial constriction of FAs[9,12,31] or actin fiber structures (e.g., stress fibers, filopodia, lamellipodia)[6,30,32,33] have been proposed to play a role in this process (Figure 5D), the contribution of each of these intracellular components to the emergence of contact guidance at intermediate length scales is still unclear.

Our combined experimental and computational work demonstrates the existence of two distinct regimes of cellular alignment, governed by the length scale of the anisotropic cue (Figure 1D). These distinct regimes have not been observed previously, as previous studies have focused on limited ranges of line widths, in some cases using microfabricated substrates that present confounding additional cues in the third dimension as well, such as grooves and ridges (see Tamiello et al.[11] for a review). For w <20 μm, myofibroblasts aligned parallel to the lines, bridging multiple lines. Decreasing *w* results in reduced cellular alignment, and at $w = s =$ 2 μm, contact guidance effect is greatly decreased. A recent study by Ramirez-San Juan et al.[30] has observed a much stronger alignment of NIH 3T3 fibroblasts on parallel fibronectin lines with $w = s = 2$ μm than that of the myofibroblasts in our study. The discrepancy between this previous study and our observations might result from the differences in cell size. Indeed, Ray et al.[9] showed that the degree to which different cell types respond to anisotropic substrates can vary significantly, and 3T3 fibroblasts are typically much smaller than human myofibroblasts.[34] The model presented in the present study suggests that the energy penalty of a gap size has in proportion a stronger effect if the cell size is smaller. Thus, the stronger alignment of 3T3 cells compared to that of myofibroblasts in response to 2 × 2 μm lines might derive from the different sizes of these two cell types. To test whether this model prediction is worth considering, we analyzed the alignment of human cardiomyocyte progenitor cells (CMPCs) (average spread length 100 μm) on our fibronectin lines. We found that CMPCs showed a very similar contact-guidance response to that of myofibroblasts (average spread length 180 μm), as expected (Figure S4). Moreover, the transition from cell alignment on single lines to alignment on multiple lines for CMPCs occurs at $s = 10$ μm, smaller than for myofibroblasts ($s = 20$ μm), in qualitative agreement with our model prediction. A future comparative study including different cell types with

systematically varying spacing between adhesive lines could further elucidate the role of cell type and size on contact guidance.

We observed that the two regimes of cellular orientation were also reflected in the orientation of the individual actin fibers and nucleus, although the two regimes were less clear for FAs. Previous studies demonstrated that FA dynamics are affected by the geometric dimensions of anisotropic substrates.[9,12,31,35] In addition, higher levels of aligned mature adhesions were observed along lines or microgrooves. However, a closer examination of the FAs of cells in regime I revealed that many FAs were not fully aligned in direction of the lines, although the cells showed a clear increase in alignment for increasing line widths (Figures 1A and 2B). Given that the typical FA size is larger than 2 μm (Figure 2D), we expected that, on 2-μm-wide lines, FAs would orient and elongate in direction of the lines. However, we found that FAs were non-aligned, smaller, and less elongated for $w = 2$ μm compared to on homogeneously coated substrates (Figures 2C and 2D). These results indicate that, at intermediate-length scales, contact guidance arises from a mechanism other than constrainment of FA alignment.

In a seminal study on contact guidance, Dunn and Heath[6] postulated that this phenomenon arises when non-adhesive gaps are wide enough to cause a mechanical restriction for the formation of actin protrusions, causing cells to align in direction of the lines. Although the alignment of filopodia and lamellipodia[22,30,33] has been proposed to play a role in this process, the exact contribution of these protrusions is not fully clear. To determine the effects of non-adhesive gaps on cell alignment, we varied s and showed that cells can still form protrusions to bridge the non-adhesive gaps of length scales much larger than the characteristic length of filopodia (~5 μm; Figures 4A and 4B).[36] This confirms that the formation of actin protrusions was not restricted and mechanical restriction of the formation of actin protrusions is not responsible for cellular contact guidance. Additionally, previous studies have also found that blocking filopodia formation did not affect contact guidance.[37] Figure 5B shows that cellular alignment is not significantly affected by increasing w but is enhanced by increasing s, indicating that the interline spacing s is a more important parameter in guiding cellular alignment than line width w.

To understand the mechanisms determining contact guidance, several computational models have been developed that account for the (re)organization of intracellular structural components, i.e., FAs, actin stress fibers, and actin protrusions. The computational model of Loosli et al.[38] successfully predicted experimental observations reported in the literature,[27] but the hypotheses at the basis of the model were phenomenological. Similarly, Vigliotti et al.[39] could predict contact guidance for cells on nanogrooved substrates[40] with a cooperative feedback model for the interplay between FA formation and actin stress fibers development. However, this approach can only be used for groove sizes that are very small compared to the cell size. Recently, an increasing number of computational models were established for cell spreading on substrates, based on the hypothesis that free energy minimization drives this biological process.[41–44] This hypothesis successfully explained the spreading of cells on smooth substrates[42] or patterned islands.[43] However, these models were unable to account for the full range of experimental observations, except by including a fitting (phenomenological) parameter for the cell fluctuations. Shishvan et al.[14] recently postulated that the fluctuations in cell morphology are constrained by the fact that cells remodel their shape and intracellular structures to maintain an overall homeostatic state over the long-term period.

Here, we extended this framework by taking the adhesive properties of the substrate into account. With this inclusion, the experimentally observed regimes of cellular alignment and aspect ratio were successfully captured.

The proposed computational framework does not include the directionality of FA formation or the dynamics of actin-rich protrusions. Still, the model was able to fully predict the two regimes of cellular alignment, clearly suggesting that alignment of FA or protrusions are not necessary ingredients for the alignment of cells in response to anisotropic adhesive lines, in agreement with our experimental results. The model further suggests that the tendency of cells to align in the direction of the lines is a direct consequence of minimizing the number of non-adhesive gaps to bridge in order to reduce the contribution of the cellular adhesion energy to the free energy. This is consistent with the observation of Romsey et al.,[29] who demonstrated that the number of lines a cell contacts affects the fidelity of contact guidance. Indeed, the average number of adhesive gaps to bridge or number of adhesive lines that each cell contacted decreased with increasing *s* (Figure 3D). Altogether, our study showed that cellular alignment is determined by the adhesion properties between the cells and the non-adhesive gaps. Future studies should be directed to dissecting in greater detail the role of dynamic processes, such as actin protrusions, in this gap-avoidance behavior. Moreover, it will be instructive to explore the role of these cellular processes in the transition between adhesion-mediated and gap-mediated contact guidance, for example, by systematically monitoring cellular responses above and below 2 μm, which is technically impossible using our current experimental approach.

To summarize, we have shown that alignment of myofibroblasts can be induced by anisotropic geometrical cues ranging from micron to hundreds-of-micron scale. Our experimental and computational results suggest that cell alignment at these length scales emerges from the tendency of cells to elongate and maximize actin polymerization, while avoiding the formation of cellular adhesions on non-adhesive gaps ("gap avoidance"). This is a generic biophysical mechanism underlying the morphological fluctuations of cells that does not require specific biochemical regulation or molecular pathway. Thus, this understanding not only offers an attractive, alternative explanation for how substrate anisotropy regulates the cellular orientation response but can also be relevant for devising new experimental strategies for directing tissue morphogenesis and regeneration.

EXPERIMENTAL PROCEDURES

Design and Fabrication of Stamps

Microcontact printing was used to pattern adhesive lines ranging from 2 μm to 200 μm. The micropatterned substrates were fabricated via standard photolithography techniques, according to previous protocols.[45] Briefly, the desired features were generated onto a silicon master by deep reactive-ion etching (Philips Innovation Services, Eindhoven, the Netherlands) from a chromium photomask (Toppan Photomask, Corbeil Essonnes, France). The silicon surface was passivated with a fluorosilane, and microstamps were obtained by molding the silanized silicon master with polydimethylsiloxane (PDMS) (Sylgard 184; Dow Corning) and curing agent (10:1), which was cured at 65°C overnight. The cured PDMS stamps containing the desired features were then peeled off from the master.

Microcontact Printing

For microcontact printing, the stamps were cleaned by sonicating in 70% ethanol for 30 min and dried using compressed air. The structured surface of the PDMS

stamps were incubated for 1 h at room temperature with a 50 μg/mL rhodamine fibronectin (FN) solution (Cytoskeleton). The substrates for printing were flat PDMS-coated glass coverslips that were oxidized in a UV/Ozone cleaner (PDS UV-ozone cleaner; Novascan, Ames, IA) for 8 min just before use. The FN-coated stamps were dried under sterile air flow and gently deposited on the substrates for 15 min at room temperature. Uncoated regions were blocked by immersing the micropatterned coverslips for 5 min in a 1% solution of Pluronic F-127 (Sigma-Aldrich). Finally, the coverslips were three times washed with phosphate-buffered saline (PBS) and stored in PBS at 4°C before use. As a control substrate, a homogeneous fibronectin coating was obtained using a flat PDMS stamp.

Cell Culture

Human vena saphena cells (HVSCs) were harvested from the vena saphena magna obtained from patients according to Dutch guidelines of secondary used material and have previously been characterized as myofibroblasts.[46] The myofibroblasts were cultured in advanced Dulbecco's modified Eagle's medium (Invitrogen, Breda, the Netherlands) supplemented with 10% fetal bovine serum (Greiner Bio-one), 1% penicillin/streptomycin (Lonza, Basel, Switzerland), and 1% GlutaMax (Invitrogen). Only cells with a passage lower than 7 were used in this study. To avoid effects on cell alignment deriving from cell-cell contacts, the microcontact printed substrates were seeded with a cell density of 2,000 cells/cm^2. The myofibroblasts were cultured for 24 h at 37°C and 5% CO_2 on these substrates.

Human fetal cardiomyocyte progenitor cells (CMPCs) were isolated and cultured as described previously.[47,48] In this study, the L9TB CMPC cell line, a kind gift from Prof. Marie-José Goumans (Leiden UMC), was used, immortalized by lentiviral transduction of hTert and BMI-1. The CMPCs were cultured in SP++ growth medium consisting of M199 (Gibco)/EGM2 (3:1) supplemented with 10% (v/v) FBS (Greiner bio-one), 1% (v/v) non-essential amino acids (Gibco), and 1% (v/v) penicillin/streptomycin (Lonza) on 0.1% (w/v) gelatin (Sigma-Aldrich)/PBS (Sigma)-coated flasks until transfer onto the microcontact printed substrates. The CMPCs were seeded with a cell density of 2,000 cells/cm^2 and were used with passage number 44–46. The CMPCs were cultured for 24 h at 37°C and 5% CO_2 on these substrates.

Immunofluorescence Labeling

After culture on the micropatterned substrates, the myofibroblasts were washed with PBS and fixed with 3.7% formaldehyde in PBS (Sigma-Aldrich) for 15 min at room temperature. The cells were permeabilized with 0.5% Triton X-100 (Merck) in PBS for 10 min at room temperature. Fixed cells were incubated for 30 min with 4% goat serum in PBS in order to block non-specific binding. Subsequently, samples were incubated with Alexa Fluor 647 goat anti-mouse (Molecular Probes) diluted at 1:500 and Phalloidin-Atto (15500, Phalloidin-Atto 488, Sigma-Aldrich) diluted at 1:200 for staining the actin cytoskeleton. Finally, the samples were incubated with DAPI (Sigma-Aldrich) diluted at 1:500 for 1 h at room temperature for immunofluorescence of the nucleus and mounted onto glass slides using Mowiol (Sigma-Aldrich). Fluorescence images were obtained with an inverted microscope (Zeiss Axiovert 200M equipped with an AxioCam HR camera; Zeiss, Sliedrecht, the Netherlands) using a 20×/0.25 Ph1 (0.68 μm/pixel) or a 40×/0.95 (0.16 μm/pixel) objective.

Live-Cell Imaging

For time-lapse imaging of cell spreading, microcontact printed coverslips were mounted in a custom-made chamber. Prior to the live-cell experiments, the cells

were transduced with Talin-GFP CellLight BacMam 2.0 (Life Technologies) according to manufacturer recommendations to stain for the FAs. Culture flasks were incubated with approximately 30 BacMam particles/cell for at least 24 h. Then, the cells were trypsinized and resuspended in 100 nM SiR-actin (Cytoskeleton) for 45 min at 37°C. This cell suspension was centrifuged for 7 min at 1,000*g* and resuspended in medium. We added 100 nM of SiR-actin to the medium and seeded the cells at 2,000 cells/cm^2 on the printed substrate. After 30 min incubation to allow cell adhesion, the sample was placed in the microscopy incubator under controlled temperature (37°C) and CO_2 (5%) conditions. Data were collected approximately 90 min after cell seeding by imaging at 512 × 512 pixels every 15–20 min over 24 h with a confocal microscope (Leica TCS SP 5 confocal microscope equipped with a Leica MC170 HD camera) using a 20×/0.7 (0.32 μm/pixel) objective.

Image Analysis

The cellular, nuclear, and FA morphological parameters were assessed from Phalloidin-Atto, DAPI, and vinculin-stained images, respectively, that were analyzed using a custom-built script publicly available either in Mathematica (Wolfram Research, Mathematica, Version 11.1, Champaign, IL, USA, 2017)[17] or in MATLAB (The Mathworks, Natick, MA, USA; https://gitlab.tue.nl:443/stem/sfalab/tree/v0.01). Briefly, the images of cells, nuclei, and FAs were binarized and fitted with an ellipse using a least-square algorithm. This was used to quantify the orientation, which was defined as the angle θ between the major axis of the ellipse and the direction of the patterned lines, where θ = 0° represents the direction of the lines. The cell orientation order can be characterized by the order parameter Θ:[49]

$$\Theta = \sqrt{\langle \cos(2\theta) \rangle^2 + \langle \sin(2\theta) \rangle^2}, \quad \text{(Equation 1)}$$

where <. > denotes ensemble averaging over all measurements. The values of Θ range from 0, representing random alignment, to 1, representing perfect alignment. To represent the shape of the cells, nuclei, and FAs, we computed the aspect ratio (i.e., the ratio of the major axis to the minor axis) and area. For the quantification of the FA orientation, only elongated FAs (aspect ratio larger than 1.6) were taken into account. Actin fiber orientation was quantified using a fiber orientation algorithm, based on the work by Frangi et al.[50]

Statistical Analysis

The data were obtained and pooled from at least three independent experiments (substrates), each containing multiple (4–8) micropatterns. For each condition, we analyzed at least 60 cells unless indicated otherwise. To assess differences between the different line widths on the morphological features of the FAs, one-way ANOVA with a Bonferroni post hoc test was used.

Modeling

To get a better understanding of the mechanisms determining the response of myofibroblasts to multiple adhesive (fibronectin) lines alternated with non-adhesive lines, we extended a recently developed statistical thermodynamics framework for cells.[14] In this study, we extended this framework to simulate cells spreading on multiple (non-)adhesive lines of different width. In what follows, the main characteristics of this framework are briefly summarized, together with the features of the model extension. A complete description of the model and numerical implementation can be found in the original model[14] and in the Supplemental Experimental Procedures.

A system comprising a single cell in contact with a substrate and surrounded by a nutrient bath was analyzed. This is an open system because the cell exchanges molecular species with the surrounding nutrient bath. Over the short-term period of seconds, this exchange is negligible and the system can be assumed as closed. Therefore, the state of the intracellular molecules over this short-term period can be predicted by Gibbs free-energy minimization as in classical statistical mechanics. Over the long-term period of minutes to hours, the exchange of molecules through the cellular membrane is not negligible. Over this time span, the cell is then out of equilibrium in terms of classical statistical mechanics and exhibits fluctuations in terms of its observables. However, it is known that cells, via intracellular biochemical processes, actively strive to maintain an average homeostatic number of molecular species within their body.[5] This, in terms of Gibbs free energy, translates to enforcing the constraint that the average Gibbs free energy of the system is constant over the long-term period.[14] By enforcing that the molecular species within the cell body minimize their Gibbs free energy over the short-term period (seconds), while the average Gibbs free energy of the system is constant over the long-term period (minutes to hours), the probability distributions of the cell orientation, aspect ratio, etc. can be obtained. Due to the cell movement and exchange of nutrients, these observables will exhibit fluctuations much larger than thermal fluctuations but proportional to a homeostatic temperature, a concept that was introduced in Shishvan et al.[14]

Similar to previous studies,[13,14] the Gibbs free energy of a cell configuration (j) was defined as

$$G^{(j)} = \int_{V_{cell}} f dV, \tag{Equation 2}$$

where V_{cell} denotes the cell volume and f is the specific Helmholtz free energy of the cell. This last term was computed as

$$f = f_{cyto} + \Phi_{elas} + f_{adh}, \tag{Equation 3}$$

where the terms f_{cyto} and Φ_{elas}, respectively associated with the stress fiber and cell passive components, were defined as in Shishvan et al.[14] Compared to previous studies, here, the term f_{adh} was added to consider the Helmholtz free energy of cell adhesions. Specifically, cell adhesions on the substrate were approximated as linear springs, and it was assumed that springs associated with non-adhesive parts of the substrate have a very low stiffness k_n compared to the stiffness k_a of springs on adhesive areas ($k_n << k_a$). From this, it follows that the Helmholtz free energy of a cell adhesion at a cell material point $\mathbf{x}$ can be computed as

$$f_{adh} = \begin{cases} \dfrac{(F(\mathbf{x}))^2}{2k_a}, & \text{if } \mathbf{x} \text{ is adhesive,} \\ \dfrac{(F(\mathbf{x}))^2}{2k_n}, & \text{if } \mathbf{x} \text{ is non} - \text{adhesive,} \end{cases} \tag{Equation 4}$$

where $F(\mathbf{x})$ is the magnitude of the force (in material point $\mathbf{x}$) that the linear spring has to exert to equilibrate the residual forces resulting from the interaction between stress fibers and cell passive components. We observe that, from $k_n <<$ k_a, it follows $\frac{(F(\mathbf{x}))^2}{2k_n} \gg \frac{(F(\mathbf{x}))^2}{2k_a}$. Consequently, cell configurations without cell adhesions on non-adhesive points have much lower values of Helmholtz (and Gibbs) free energy and are considerably more likely to occur. Therefore, the approach efficiently models the preference of cells to form and mature adhesions only on adhesive lines.

Material Parameters for Myofibroblasts

A single set of material parameters was adopted and unchanged for all simulations of this study. Most of the model parameters describe inherent properties of stress fiber proteins that are not expected to differ among cell types. Differences among cell types are recapitulated in terms of the stress fiber protein volume fraction F_0, the passive elastic properties described by the shear modulus of the cell (μ), the in-plane bulk modulus of the cell k, and m (a material constant that describes the nonlinearity of the deviatoric elastic cell response). Overall, increasing the term F_0 causes a widening of the Gibbs free-energy distribution and an increase of the homeostatic area of a cell on a homogeneous substrate, although an increase of the cellular elastic properties has the opposite effect. In addition to these parameters, the homeostatic area can also change because of the inherent size of a cell type. In the model, this parameter is represented by the scaling parameter l_w. For more details on the model parameters, we refer the reader to the Supplemental Information and to previous studies.[14,51]

The parameters adopted in the present study are based on validations performed in previous works.[14,52] In particular, all parameters except for k_a and k_n were taken in agreement with Shishvan et al.[14] (k_a and k_n were not present in this previous study). Shishvan et al. demonstrated that, with these parameters, the model can predict morphological features (e.g., area and aspect ratio) of smooth muscle cells spreading on substrates with different stiffness. Myofibroblasts have very similar mechanical properties as smooth muscle cells, and therefore, the same parameters were adopted in the present study. The parameter k_a was then chosen such that the focal adhesion energy computed for cells on homogeneous and stiff substrates was similar to the energy reported in McEvoy et al.,[52] who analyzed the focal adhesion free energy for cells on substrates with different rigidities. Finally, k_a/k_n = 100 was chosen such that adhesions formed on non-adhesive areas were hardly observed in the simulations. A list of the material parameters is given in Table S1.

Computational Simulation

The cell in the initial, undeformed (circular) configuration was discretized with three-noded triangular elements. Different configurations (j) were then obtained by varying the displacements $\mathbf{u}^{(j)}$, which uniquely identify the strains in the cell. From these values, by considering the chemical equilibrium in the cell body, f_{cyto} and Φ_{elas} are computed, as well as the passive stress within the cell and the stress fiber stress. Given these passive and active stress values, the residual traction forces $T(\mathbf{x}^{(j)})$ can be identified at each node, together with the consequential values of $F(\mathbf{x}^{(j)}) = -T(\mathbf{x}^{(j)})$. From these forces, the Helmholtz free energy f_{adh} and the total Gibbs free energy can be computed. For obtaining the statistics of cell shapes on a substrate, the homeostatic Gibbs free energy and the associated homeostatic temperature were first computed iteratively using Metropolis algorithm, analyzing 2,000,000 cell-shape perturbations. The same procedure was then repeated to simulate cells on microcontact printed substrates. This was achieved without changing the material parameters but by changing the homeostatic temperature of the system to enforce the condition that the average Gibbs free energy of a cell in every substrate is equal to the homeostatic energy found for the free-standing cell. See Supplemental Experimental Procedures for a complete description of the simulations.

The morphologies of representative cells from the simulations and experiments are compared in Figure S6. In general, the cellular shapes obtained with the

computational simulations are comparable to those experimentally observed. For each cell shape resulting from the simulations, the cellular orientation and aspect ratio were obtained by fitting the outline of the cell with the best-fitting ellipse (in a least-square sense), as in the experiments. The statistics of these simulations are reported in Figure S5.

Data and Code Availability

Codes used to analyze the experimental images are publicly available at https://gitlab.tue.nl:443/stem/sfalab/tree/v0.01 and https://doi.org/10.1371/journal.pone.0195201. Codes used to perform the computational modeling of cells are publicly available at https://zenodo.org/record/3710132. All data associated with the study are included in the paper and the Supplemental Information or from the lead author upon reasonable request.

SUPPLEMENTAL INFORMATION

Supplemental Information can be found online at https://doi.org/10.1016/j.xcrp.2020.100055.

ACKNOWLEDGMENTS

The authors thank Prof. M.-J. Goumans for kindly sharing CMPC cell line. This work has been supported by the Impuls Program of the Eindhoven University of Technology; the Netherlands Cardio Vascular Research Initiative: The Dutch Heart Foundation, Dutch Federation of University Medical Centers, the Netherlands Organization for Health Research, and Development and the Royal Netherlands Academy of Sciences ("CVON 1Valve"); the Ministry of Education, Culture and Science for the Gravitation Program 024.003.103 "Materials Driven Regeneration"; and the European Research Council (grant 851960; N.A.K.).

AUTHOR CONTRIBUTIONS

Conceptualization, A.B.C.B., N.A.K., and C.V.C.B.; Methodology, A.B.C.B. and D.M.; Software, T.R., S.S.S., and V.S.D.; Investigation, A.B.C.B., T.R., D.M., and M.C.v.T.; Resources, S.S.S., M.C.v.T., N.A.K., V.S.D., and C.V.C.B.; Writing – Original Draft, A.B.C.B., T.R., and N.A.K.; Writing – Review & Editing, S.S.S., S.L., V.S.D., and C.V.C.B.; Visualization, A.B.C.B.; Supervision, S.L., N.A.K., V.S.D., and C.V.C.B.; Funding Acquisition, C.V.C.B.

DECLARATION OF INTERESTS

The authors declare no competing interests.

Received: October 4, 2019
Revised: February 28, 2020
Accepted: March 20, 2020
Published: May 20, 2020

REFERENCES

1. Feinberg, A.W., Alford, P.W., Jin, H., Ripplinger, C.M., Werdich, A.A., Sheehy, S.P., Grosberg, A., and Parker, K.K. (2012). Controlling the contractile strength of engineered cardiac muscle by hierarchal tissue architecture. Biomaterials *33*, 5732–5741.

2. Barthes, J., Özçelik, H., Hindié, M., Ndreu-Halili, A., Hasan, A., and Vrana, N.E. (2014). Cell microenvironment engineering and monitoring for tissue engineering and regenerative medicine: the recent advances. BioMed Res. Int. *2014*, 921905.

3. Gasiorowski, J.Z., Murphy, C.J., and Nealey, P.F. (2013). Biophysical cues and cell behavior: the big impact of little things. Annu. Rev. Biomed. Eng. *15*, 155–176.

4. Werner, M., Kurniawan, N.A., and Bouten, C.V.C. (2020). Cellular geometry sensing at different length scales and its implications for scaffold design. Materials (Basel) *13*, 963.

5. Weiss, P. (1945). Experiments on cell and axon orientation in vitro; the role of colloidal exudates in tissue organization. J. Exp. Zool. *100*, 353–386.

6. Dunn, G.A., and Heath, J.P. (1976). A new hypothesis of contact guidance in tissue cells. Exp. Cell Res. *101*, 1–14.

7. Wieringa, P., Tonazzini, I., Micera, S., and Cecchini, M. (2012). Nanotopography induced contact guidance of the F11 cell line during neuronal differentiation: a neuronal model cell line for tissue scaffold development. Nanotechnology *23*, 275102.

8. Lee, G., Atia, L., Lan, B., Sharma, Y., Nissim, L., Wu, M.R., Pery, E., Lu, T.K., Park, C.Y., Butler, J.P., and Fredberg, J.J. (2018). Contact guidance and collective migration in the advancing epithelial monolayer. Connect. Tissue Res. *59*, 309–315.

9. Ray, A., Lee, O., Win, Z., Edwards, R.M., Alford, P.W., Kim, D.-H., and Provenzano, P.P. (2017). Anisotropic forces from spatially constrained focal adhesions mediate contact guidance directed cell migration. Nat. Commun. *8*, 14923.

10. Kurniawan, N.A. (2019). The ins and outs of engineering functional tissues and organs: evaluating the in-vitro and in-situ processes. Curr. Opin. Organ Transplant. *24*, 590–597.

11. Tamiello, C., Buskermolen, A.B.C., Baaijens, F.P.T., Broers, J.L.V., and Bouten, C.V.C. (2016). Heading in the right direction: understanding cellular orientation responses to complex biophysical environments. Cell. Mol. Bioeng. *9*, 12–37.

12. Saito, A.C., Matsui, T.S., Ohishi, T., Sato, M., and Deguchi, S. (2014). Contact guidance of smooth muscle cells is associated with tension-mediated adhesion maturation. Exp. Cell Res. *327*, 1–11.

13. Buskermolen, A.B.C., Suresh, H., Shishvan, S.S., Vigliotti, A., DeSimone, A., Kurniawan, N.A., Bouten, C.V.C., and Deshpande, V.S. (2019). Entropic forces drive cellular contact guidance. Biophys. J. *116*, 1994–2008.

14. Shishvan, S.S., Vigliotti, A., and Deshpande, V.S. (2018). The homeostatic ensemble for cells. Biomech. Model. Mechanobiol. *17*, 1631–1662.

15. Lehnert, D., Wehrle-Haller, B., David, C., Weiland, U., Ballestrem, C., Imhof, B.A., and Bastmeyer, M. (2004). Cell behaviour on micropatterned substrata: limits of extracellular matrix geometry for spreading and adhesion. J. Cell Sci. *117*, 41–52.

16. Xia, N., Thodeti, C.K., Hunt, T.P., Xu, Q., Ho, M., Whitesides, G.M., Westervelt, R., and Ingber, D.E. (2008). Directional control of cell motility through focal adhesion positioning and spatial control of Rac activation. FASEB J. *22*, 1649–1659.

17. Buskermolen, A.B.C., Kurniawan, N.A., and Bouten, C.V.C. (2018). An automated quantitative analysis of cell, nucleus and focal adhesion morphology. PLoS ONE *13*, e0195201.

18. Chalut, K.J., and Paluch, E.K. (2016). The actin cortex: a bridge between cell shape and function. Dev. Cell *38*, 571–573.

19. Goffin, J.M., Pittet, P., Csucs, G., Lussi, J.W., Meister, J.J., and Hinz, B. (2006). Focal adhesion size controls tension-dependent recruitment of alpha-smooth muscle actin to stress fibers. J. Cell Biol. *172*, 259–268.

20. Izzard, C.S., and Lochner, L.R. (1976). Cell-to-substrate contacts in living fibroblasts: an interference reflexion study with an evaluation of the technique. J. Cell Sci. *21*, 129–159.

21. Suresh, H., Shishvan, S.S., Vigliotti, A., and Deshpande, V.S. (2019). Free-energy-based framework for early forecasting of stem cell differentiation. J. R. Soc. Interface *16*, 20190571.

22. Kubow, K.E., Shuklis, V.D., Sales, D.J., and Horwitz, A.R. (2017). Contact guidance persists under myosin inhibition due to the local alignment of adhesions and individual protrusions. Sci. Rep. *7*, 14380.

23. Chien, K.R., Domian, I.J., and Parker, K.K. (2008). Cardiogenesis and the complex biology of regenerative cardiovascular medicine. Science *322*, 1494–1497.

24. Sharma, B., and Elisseeff, J.H. (2004). Engineering structurally organized cartilage and bone tissues. Ann. Biomed. Eng. *32*, 148–159.

25. Provenzano, P.P., and Vanderby, R., Jr. (2006). Collagen fibril morphology and organization: implications for force transmission in ligament and tendon. Matrix Biol. *25*, 71–84.

26. Clark, P., Connolly, P., and Moores, G.R. (1992). Cell guidance by micropatterned adhesiveness in vitro. J. Cell Sci. *103*, 287–292.

27. Zimerman, B., Arnold, M., Ulmer, J., Blümmel, J., Besser, A., Spatz, J.P., and Geiger, B. (2004). Formation of focal adhesion-stress fibre complexes coordinated by adhesive and non-adhesive surface domains. IEE Proc., Nanobiotechnol. *151*, 62–66.

28. Rossier, O.M., Gauthier, N., Biais, N., Vonnegut, W., Fardin, M.A., Avigan, P., Heller, E.R., Mathur, A., Ghassemi, S., Koeckert, M.S., et al. (2010). Force generated by actomyosin contraction builds bridges between adhesive contacts. EMBO J. *29*, 1055–1068.

29. Romsey, N.R., Hou, Y., Rodriguez, L.L., and Schneider, I.C. (2014). The number of lines a cell contacts and cell contractility drive the efficiency of contact guidance. Cell. Mol. Bioeng. *7*, 122–135.

30. Ramirez-San Juan, G.R., Oakes, P.W., and Gardel, M.L. (2017). Contact guidance requires spatial control of leading-edge protrusion. Mol. Biol. Cell *28*, 1043–1053.

31. Ohara, P.T., and Buck, R.C. (1979). Contact guidance in vitro. A light, transmission, and scanning electron microscopic study. Exp. Cell Res. *121*, 235–249.

32. Teixeira, A.I., Abrams, G.A., Bertics, P.J., Murphy, C.J., and Nealey, P.F. (2003). Epithelial contact guidance on well-defined micro- and nanostructured substrates. J. Cell Sci. *116*, 1881–1892.

33. Sales, A., Holle, A.W., and Kemkemer, R. (2017). Initial contact guidance during cell spreading is contractility-independent. Soft Matter *13*, 5158–5167.

34. Rajagopalan, P., Marganski, W.A., Brown, X.Q., and Wong, J.Y. (2004). Direct comparison of the spread area, contractility, and migration of balb/c 3T3 fibroblasts adhered to fibronectin- and RGD-modified substrata. Biophys. J. *87*, 2818–2827.

35. den Braber, E.T., de Ruijter, J.E., Ginsel, L.A., von Recum, A.F., and Jansen, J.A. (1998). Orientation of ECM protein deposition, fibroblast cytoskeleton, and attachment complex components on silicone microgrooved surfaces. J. Biomed. Mater. Res. *40*, 291–300.

36. Schäfer, C., Faust, U., Kirchgessner, N., Merkel, R., and Hoffmann, B. (2011). The filopodium: a stable structure with highly regulated repetitive cycles of elongation and persistence depending on the actin cross-linker fascin. Cell Adhes. Migr. *5*, 431–438.

37. Rajnicek, A., and McCaig, C. (1997). Guidance of CNS growth cones by substratum grooves and ridges: effects of inhibitors of the cytoskeleton, calcium channels and signal transduction pathways. J. Cell Sci. *110*, 2915–2924.

38. Loosli, Y., Vianay, B., Luginbuehl, R., and Snedeker, J.G. (2012). Numerically bridging lamellipodial and filopodial activity during cell spreading reveals a potentially novel trigger of focal adhesion maturation. Integr. Biol. *4*, 508–521.

39. Vigliotti, A., McMeeking, R.M., and Deshpande, V.S. (2015). Simulation of the cytoskeletal response of cells on grooved or patterned substrates. J. R. Soc. Interface *12*, 20141320.

40. Lamers, E., Walboomers, X.F., Domanski, M., te Riet, J., van Delft, F.C.M.J.M., Luttge, R., Winnubst, L.A.J.A., Gardeniers, H.J.G.E., and Jansen, J.A. (2010). The influence of nanoscale grooved substrates on osteoblast behavior and extracellular matrix deposition. Biomaterials *31*, 3307–3316.

41. Jiang, L., Yang, C., Zhao, L., and Zheng, Q. (2014). Stress fiber response to mechanics: a free energy dependent statistical model. Soft Matter *10*, 4603–4608.

42. McEvoy, E., Deshpande, V.S., and McGarry, P. (2017). Free energy analysis of cell spreading. J. Mech. Behav. Biomed. Mater. *74*, 283–295.

43. Vianay, B., Käfer, J., Planus, E., Block, M., Graner, F., and Guillou, H. (2010). Single cells spreading on a protein lattice adopt an energy minimizing shape. Phys. Rev. Lett. *105*, 128101.

44. Livne, A., Bouchbinder, E., and Geiger, B. (2014). Cell reorientation under cyclic stretching. Nat. Commun. *5*, 3938.

45. Yang, M.T., Fu, J., Wang, Y.K., Desai, R.A., and Chen, C.S. (2011). Assaying stem cell mechanobiology on microfabricated elastomeric substrates with geometrically modulated rigidity. Nat. Protoc. *6*, 187–213.

46. Mol, A., Rutten, M.C., Driessen, N.J., Bouten, C.V., Zünd, G., Baaijens, F.P., and Hoerstrup, S.P. (2006). Autologous human tissue-engineered heart valves: prospects for systemic application. Circulation *114*, 152–159.

47. Goumans, M.J., de Boer, T.P., Smits, A.M., van Laake, L.W., van Vliet, P., Metz, C.H., Korfage, T.H., Kats, K.P., Hochstenbach, R.,

Pasterkamp, G., et al. (2007). TGF-beta1 induces efficient differentiation of human cardiomyocyte progenitor cells into functional cardiomyocytes in vitro. Stem Cell Res. *1*, 138–149.

48. Smits, A.M., van Vliet, P., Metz, C.H., Korfage, T., Sluijter, J.P., Doevendans, P.A., and Goumans, M.J. (2009). Human cardiomyocyte progenitor cells differentiate into functional mature cardiomyocytes: An in vitro model for studying human cardiac physiology and pathophysiology. Nat. Protoc. *4*, 138–149.

49. Duclos, G., Garcia, S., Yevick, H.G., and Silberzan, P. (2014). Perfect nematic order in confined monolayers of spindle-shaped cells. Soft Matter *10*, 2346–2353.

50. Frangi, A.F., Niessen, W.J., Vincken, K.L., and Viergever, M.A. (1998). Multiscale vessel enhancement filtering. In Medical Image Computing and Computer-Assisted Inrvervention – MICCAI'98. MICCAI 1998. Lecture Notes in Computer Science, W.M. Wells, A. Colchester, and S. Delp, eds. (Springer), pp. 130–137.

51. Vigliotti, A., Ronan, W., Baaijens, F.P.T., and Deshpande, V.S. (2016). A thermodynamically motivated model for stress-fiber reorganization. Biomech. Model. Mechanobiol. *15*, 761–789.

52. McEvoy, E., Shishvan, S.S., Deshpande, V.S., and McGarry, J.P. (2018). Thermodynamic modeling of the statistics of cell spreading on ligand-coated elastic substrates. Biophys. J. *115*, 2451–2460.

Cell Reports Physical Science, Volume *1*

Supplemental Information

Cellular Contact Guidance

Emerges from Gap Avoidance

Antonetta B.C. Buskermolen, Tommaso Ristori, Dylan Mostert, Mark C. van Turnhout, Siamak S. Shishvan, Sandra Loerakker, Nicholas A. Kurniawan, Vikram S. Deshpande, and Carlijn V.C. Bouten

Supplemental Experimental Procedures

Modeling framework

To predict the shape and orientation of cells on multiple adhesive lines of fibronectin, we extended the statistical framework of Shishvan et al. [1], which is here briefly described. In what follows, we refer to: "the cell", to indicate a generic cell to model; "the substrate", as a generic substrate with alternating (non-)adhesive lines; "cellular configuration", to indicate the mapping of the cell material points on the substrate; and "the system", as the system composed of the cell and the substrate.

Characterization of a configuration

The cell was approximated as a two-dimensional body in the $x_1 - x_2$ plane, with through-thickness stress $\Sigma_{33} = 0$. $\boldsymbol{X}$ indicates the coordinates of each material point of the cell in the undeformed configuration, omitting the index of all different material points for brevity. The cell in the undeformed configuration was assumed cylindrical, with radius R_0 and thickness $b_0 = R_0/5$. The other configurations were characterized by the displacements of each material point $\boldsymbol{u}^{(j)}(\boldsymbol{X}) \equiv \boldsymbol{u}^{(j)}$, such that $\boldsymbol{x}^{(j)} = \boldsymbol{X} + \boldsymbol{u}^{(j)}$ are the coordinates of the material points of the cell in the configuration with index (j).

The probability of a configuration

By assuming a rigid and purely elastic substrate not deformed by cells, the Gibbs free-energy of a configuration (j) was defined as:

$$G^{(j)} = \int_{V_{cell}} f dV, \tag{1}$$

where V_{cell} denotes the volume of the cell and f is the specific Helmholtz free-energy of the cell. As described in Shishvan et al. [1], by assuming Gibbs free-energy minimization over the short-term period (seconds) and homeostasis over the long-term period (minutes), it can be derived that a configuration (j) has probability

$$P_{eq}^{(j)} = \frac{1}{Z} \exp\left[-\zeta G^{(j)}\right], \tag{2}$$

where $Z = \sum_j \exp\left[-\zeta G^{(j)}\right]$ is the partition function and ζ is a constant such that

$$\sum_j P_{eq}^{(j)} G^{(j)} = \frac{1}{Z} \sum_j G^{(j)} \exp\left[-\zeta G^{(j)}\right] = \bar{G}. \tag{3}$$

Here, $\bar{G}$ is the homeostatic Gibbs free-energy, corresponding to the free-energy of a free-standing cell (i.e. isolated, not interacting with any substrate). The average magnitude of the fluctuations of the energy of a cell on a specific substrate is proportional to $1/\zeta$, which is referred to as homeostatic temperature [1]. This quantity is a characteristic of the interaction between the cell and the substrate, and was computed for each different substrate.

Definition of the specific Helmholtz free-energy f

In Shishvan et al. [1], the Helmholtz free-energy f was computed as $f = f_{cyto} + \Phi_{elas}$, where f_{cyto} and Φ_{elas} are the specific Helmholtz free-energies associated with stress fibers and other cellular passive components (e.g. cell membrane), respectively. Here, we added a term f_{adh} corresponding to the specific Helmholtz free-energy of cell adhesions to account for the heterogeneity of the substrate in exam, where cells can easily form adhesions on the adhesive lines, while the formation of cell adhesions on the non-adhesive lines is obstructed:

$$f = f_{cyto} + \Phi_{elas} + f_{adh}. \tag{4}$$

The terms f_{cyto} and Φ_{elas} were computed as in [1], by using the model of Vigliotti et al. [2] to compute f_{cyto}, and a two-dimensional adaptation of the Ogden hyperelastic model [3] to calculate Φ_{elas}. In what follows, the computation of these terms is summarized, followed by the definition of the term f_{adh} introduced in the present study.

The specific Helmholtz free-energy of stress fibers f_{cyto}

Vigliotti et al. [2] envisioned stress fibers as formed by a number of functional units with reference length l_0. The quantities describing stress fibers are defined in a representative volume element (RVE) that, in its undeformed configuration, is defined as a cylinder with the axis along the thickness of the two-dimensional cell, radius $n^R l_0/2$, and thickness b_0 (such as the cell). Stress fibers initiate from the center of this RVE, oriented at angles ϕ $(-\pi/2 \leq \phi < \pi/2)$ with respect to the axis x_1. In the undeformed state, n^R functional units are present in the stress fibers in each direction while, along the thickness, n_s layers of identically distributed stress fibers are present. Given an infinitesimal area of the cylinder of the RVE oriented at an angle ϕ, the number of stress fibers crossing this area is $d\Pi = \eta d\phi$, such that $\eta(\phi)$ is defined as the angular stress fiber concentration.

When a nominal strain $\varepsilon_{nom}(\phi)$ is applied along the normal of the surface dA, the stress fibers in that direction deform and, in response, remodel. In particular, due to the deformation of stress fibers and their functional units, the number of these functional units increases (in case of tensile strain) or decreases (for compressive strain) till their deformed length reaches an optimal value l_{ss}. It follows that, in addition to the nominal strain of the RVE, we can define a nominal strain $\tilde{\varepsilon}_{nom}(\phi)$ for the stress fiber functional units, relative to l_0. A relationship between $\varepsilon_{nom}(\phi)$ and $\tilde{\varepsilon}_{nom}(\phi)$ can be found by observing that $n_0 \equiv n^R [1 + \varepsilon_{nom}]$ functional units of length l_0 are necessary to cover the deformed stress fiber length $n^R l_0 [1 + \varepsilon_{nom}]$. In contrast, if n functional units are present along such length, each of them has the length $n^R l_0 [1 + \varepsilon_{nom}]/n$, and thus

$$\tilde{\varepsilon}_{nom} = \frac{n^R l_0 [1 + \varepsilon_{nom}]}{l_0 n} - 1 = \frac{n_0}{n} - 1. \tag{5}$$

At steady-state each unit has length l_{ss}, thus

$$\tilde{\varepsilon}_{nom} = l_{ss}/l_0 - 1 \equiv \tilde{\varepsilon}^{ss}_{nom}, \tag{6}$$

and

$$n^{ss} = n^R \frac{[1 + \varepsilon_{nom}]}{[1 + \tilde{\varepsilon}^{ss}_{nom}]} \tag{7}$$

are the nominal strain and number of stress fiber functional units along a direction ϕ at steady-state.

At this point, we define

$$N_b \equiv \int_{-\pi/2}^{\pi/2} \eta n^{ss} d\phi \tag{8}$$

as the total number of stress fiber functional units within the entire RVE at steady-state. At the same time, within the same RVE, there are a number of unbound functional units N_u, such that the total number of functional units within the RVE is $N_T = N_b + N_u$. In the time-scale analyzed in this study, the total number of functional units within the cell normalized over the cell volume was assumed to be constant and it was denoted with $N_0 \equiv \int_{V_{cell}} N_T dV/V_0$, where V_{cell} and V_0 represent the volume of the cell in the deformed and undeformed configurations, respectively. Given the conservation of this value, normalized quantities were defined as $\hat{N}_u \equiv N_u/N_0$, $\hat{N}_T \equiv N_T/N_0$, $\hat{N}_b = N_b/N_0$, $\hat{\eta} \equiv \eta n^R/N_0$, and $\hat{n}^{ss} \equiv n^{ss}/n^R$.

When the cell is at steady-state, $\dot{\varepsilon}_{nom} = \dot{\tilde{\varepsilon}}_{nom} = 0$, and from the assumption that stress fibers exert tension with mechanisms similar to striated muscle, it follows that in these conditions they exert the maximum stress σ_{max}. For these conditions, the chemical potential associated with each functional unit that is part of a stress fiber is

$$\mathcal{X}_b = \frac{\mu_b}{n^R} + kT \ln \left[\left(\frac{\pi \hat{\eta} \hat{n}^{ss}}{\hat{N}_u \left(1 - \frac{\hat{\eta}}{\hat{\eta}_{max}}\right)} \right)^{\frac{1}{n^{ss}}} \left(\frac{\hat{N}_u}{\pi \hat{N}_L} \right) \right] \tag{9}$$

where the first term on the right-hand side accounts for the enthalpy μ_b of n^R functional units, while the second term considers the entropy of mixing between packets of unbound and bound stress fiber proteins with sites for bound proteins, and the entropy of mixing between unbound proteins and lattice sites. For a complete derivation of this chemical potential, we refer the reader to [1]. In Eq. (9), $\hat{\eta}_{max}$ represents the maximum value of $\hat{\eta}$ such that all available lattice sites N_L are occupied, while $\hat{N}_L \equiv N_L/N_0$. The enthalpy μ_b of bound proteins can be quantified as

$$\mu_b = \mu_{b0} - \sigma_{max}\Omega(1 + \tilde{\varepsilon}^{ss}_{nom}), \tag{10}$$

where μ_{b0} is the internal energy of n^R bound stress fiber functional units and $\Omega \equiv A_0 n^R l_0$ is the volume of n^R functional units of length l_0. The chemical potential associated with every unbound functional unit is

$$\mathcal{X}_u = \frac{\mu_u}{n^R} + kT \ln \left[\frac{\hat{N}_u}{\pi \hat{N}_L} \right], \tag{11}$$

with μ_u the internal energy of unbound functional units potentially forming n^R stress fiber units. Now, given $\rho_0 \equiv N_0/V_{cell}$, the specific Helmholtz free-energy accounting for the stress fiber cytoskeleton is

$$f_{cyto} = \rho_0 \left(\hat{N}_u \mathcal{X}_u + \int_{-\pi/2}^{\pi/2} \hat{\eta} \hat{n}^{ss} \mathcal{X}_b d\phi \right). \tag{12}$$

This quantity can be computed, given the previous definitions, once calculated $\hat{\eta}$ and $\hat{N}_u$. Note that f_{cyto} has to be computed for equilibrium morphological microstates, which means $d\mathcal{G}^{(j)} = 0$. It can

be demonstrated [1] that this requirements translates to the constraints of chemical and mechanical equilibria $\mathcal{X}_b = \mathcal{X}_u$ and $\Sigma_{ij} = 0$, where Σ_{ij} is the total Cauchy stress, characterized as a summation between the stress fiber stress, the stress from the cellular adhesions, and the passive stress of the other passive cellular components. From the chemical equilibrium $\mathcal{X}_b = \mathcal{X}_u$ and Eqs. (9) and (11), an expression for $\hat{\eta}$ in terms of $\hat{N}_u$ follows:

$$\hat{\eta} = \frac{\hat{N}_u \hat{\eta}_{max} \exp\left[\frac{\hat{n}^{ss}(\mu_u - \mu_b)}{kT}\right]}{\hat{n}^{ss}\hat{\eta}_{max} + \hat{N}_u \exp\left[\frac{\hat{n}^{ss}(\mu_u - \mu_b)}{kT}\right]}. \tag{13}$$

The normalized value $\hat{N}_u$ can be computed by recalling that $N_T = N_u + N_b$ is the total number of functional units in a material point x_i, while $N_0 V_0 / V_R$ is the total number of functional units that can be formed within the cell. These two quantities are related as

$$\frac{N_0 V_0}{V_R} = \int_{V_{cell}} N_T dV = \int_{V_{cell}} N_u dV + \int_{V_{cell}} N_b dV. \tag{14}$$

The integral $\int_{V_{cell}} N_u dV$ can be easily computed because, from the chemical equilibrium, it follows that $\mathcal{X}_u$ is constant across the cell and therefore, due to Eq. (11), also N_u is constant. Now, from the definitions of $\hat{N}_u = N_u/N_0$ and $\hat{N}_b = N_b/N_0$ and (14), we have

$$1 = \hat{N}_u + \frac{1}{V_0} \int_{V_{cell}} \int_{-\pi/2}^{\pi/2} \hat{\eta} \hat{n}^{ss} d\phi dV, \tag{15}$$

from which $\hat{N}_u$ can be computed, since it is the only unknown (given Eq. (13)).

The specific Helmholtz free-energy of cellular passive components Φ_{elas}

The specific Helmholtz free-energy of passive components of the cell was modeled with a modified version of the Ogden hyperelastic strain energy density function, with the modification necessary to specialize the formulation to a two-dimensional setting. In particular,

$$\begin{aligned} \Phi_{elas} \equiv \frac{2\mu}{m^2}\left[\left(\frac{\lambda_I}{\lambda_{II}}\right)^{\frac{m}{2}} + \left(\frac{\lambda_{II}}{\lambda_I}\right)^{\frac{m}{2}} - 2\right] + \frac{\kappa}{2}(\lambda_1 \lambda_{II} - 1)^2 - \\ - \bar{\kappa}\mathcal{H}(J_c - \lambda_I \lambda_{II}) \ln(\lambda_I \lambda_{II} + 1 - J_c) \end{aligned} \tag{16}$$

where μ is the shear modulus, m a material constant, λ_I and λ_{II} are the principal stretches, κ is the in-plane bulk modulus, while $\bar{k}$ and J_c are material parameters introduced to account for the penalty associated with cellular configurations with an areal stretch $\lambda_I \lambda_{II}$ below a threshold J_c. For a complete derivation and motivation of Eq. (16), we refer the reader to [1].

The specific Helmholtz free-energy of cell adhesions f_{adh}

Cell adhesions on the substrate were approximated as linear springs, and it was assumed that springs associated with non-adhesive parts of the substrate have a very low stiffness k_n compared to the stiffness k_a of springs on adhesive areas ($k_n \ll k_a$). From this it follows that

$$f_{adh} = \begin{cases} \frac{(F(\boldsymbol{x}^{(j)}))^2}{2k_a}, & \text{if } \boldsymbol{x}^{(j)} \text{ is adhesive,} \\ \\ \frac{(F(\boldsymbol{x}^{(j)}))^2}{2k_n}, & \text{if } \boldsymbol{x}^{(j)} \text{ is non-adhesive,} \end{cases} \tag{17}$$

where $F(\boldsymbol{x}^{(j)})$ is the magnitude of the force (in material point $\boldsymbol{x}^{(j)}$) that the linear spring has to exert to equilibrate the residual forces resulting from the interaction between stress fibers and cell passive components. From $k_n \ll k_a$ it follows $\frac{(F(\boldsymbol{x}^{(j)}))^2}{2k_n} \gg \frac{(F(\boldsymbol{x}^{(j)}))^2}{2k_a}$; therefore, the approach efficiently models the preference of cells to form and mature adhesions only on adhesive lines (Eqs. (1)-(17)).

The magnitude of the force of cellular adhesions

In the present study, we assumed that cellular adhesions exert forces of a magnitude equal to the forces necessary to mechanically equilibrate the active and passive forces of the cell. This translates to assume that, given the total Cauchy stress Σ_{ij} as a summation between the stress fiber stress, the stress from cellular adhesions, and the passive stress of the other passive cellular components, we have

$$0 = \Sigma_{ij} = \sigma_{ij}^{sf} + \sigma_{ij}^{p} + \sigma_{ij}^{adh}, \tag{18}$$

with σ_{ij}^{sf} stress fiber stress, σ_{ij}^{adh} stress due to cellular adhesions, and σ_{ij}^{p} stress from other passive cellular components. It follows that, to quantify the magnitude of the force of cellular adhesions, computing σ_{ij}^{sf} and σ_{ij}^{p} is sufficient.

In agreement with [1], the stress fiber stress was computed as

$$\begin{bmatrix} \sigma_{11}^{sf} & \sigma_{12}^{sf} \\ \sigma_{12}^{sf} & \sigma_{22}^{sf} \end{bmatrix} = \frac{\mathcal{F}_0 \sigma_{max}}{2} \int_{-\pi/2}^{\pi/2} \hat{\eta}[1 + \varepsilon_{nom}(\phi)] \begin{bmatrix} 2\cos^2\phi^* & \sin 2\phi^* \\ \sin 2\phi^* & 2\sin^2\phi^* \end{bmatrix} d\phi, \tag{19}$$

with $\mathcal{F}_0 \equiv n_s(A_0 l_0)\rho_0$, while ϕ^* is the angle with respect to x_1, in the deformed configuration, that stress fibers originally oriented at an angle ϕ have in the deformed configuration. Note that Eq. (19) was determined with the assumption the cell is incompressible. This assumption, leads also to the determination of the Cauchy stress of the passive components of the cell:

$$\sigma_{ij}^{p} p_j^{(k)} = \sigma_k^{p} p_i^{(k)} \tag{20}$$

where $\sigma_k^p \equiv \lambda_k \frac{\partial \Phi_{elas}}{\partial \lambda_k}$ is the principal passive stress, with associated $p_i^{(k)}$ and $p_j^{(k)}$ unit vectors indicating the principal directions ($k = I, II$).

Numerical method for computing the Gibbs free-energy of a configuration

The cell in the undeformed configuration was discretized with three-noded triangular elements of size $R_0/10$. Different configurations (j) were then obtained by varying the displacements $\boldsymbol{u}^{(j)}$, which uniquely identify the strains in the cell. From these values, by considering the chemical equilibrium in the cell body, f_{cyto} and Φ_{elas} can be computed, as well as the passive stress within the cell and the stress fiber stress. Given these passive and active stress values, the residual traction forces $T(\boldsymbol{x}^{(j)})$ can be identified at each node, together with the consequential values of $F(\boldsymbol{x}^{(j)}) = -T(\boldsymbol{x}^{(j)})$. From

these forces, the Helmholtz free-energy f_{adh} (Eq. (17)) and the total Gibbs free-energy (Eqs. (1) and (4)) can be computed.

For the calculation of the statistics of cell shapes on a substrate (Eq. (17)), the values of $\bar{G}$ and ζ needs to be determined. First, the homeostatic energy $\bar{G}$ of a free-standing cell was computed. Only one equilibrium configuration is possible for a free-standing cell: the cell needs to be traction free and in chemical equilibrium without the contribution of the adhesions with the substrate. The total Gibbs free-energy can be computed for this unique configuration: $\bar{G} = \sum_j P_{eq}^{(j)} G^{(j)} = P_{eq}^1 G^1 = G^1$, where the index 1 refers to the unique configuration of the free-standing cell (which has $P_{eq}^1 = 1$). An iterative method was pursued to calculate ζ. Given a hypothesized value ζ_0, the corresponding average Gibbs free-energy $G_0 = \frac{1}{Z} \sum_j G^{(j)} \exp\left[-\zeta_0 G^{(j)}\right]$ was approximated with the Metropolis algorithm [4]. If $G_0 = \bar{G}$ (with a maximum absolute error equal to $2\%\bar{G}$), ζ_0 was accepted; otherwise, the procedure was repeated with a new value ζ_1. For the random walks among the possible configurations required for the Metropolis algorithm, M points were chosen among the material points of the cell. Hypothetical and random deformations $U_L^{(j)}$ (with $L = 1, \text{âĂę}, M$) were assigned to these points. The actual deformations $\boldsymbol{u}^{(j)}$ of the entire cell were generated via Non-Uniform Rational B-Splines [5], by using the values $U_L^{(j)}$ as weights. A perturbation of a specific configuration was then performed by varying the value of one of the weights $U_L^{(j)}$.

Material parameters for myofibroblasts

The material parameters were chosen similar to Buskermolen et al. [6], who simulated myofibroblasts with the same statistical framework, omitting the specific Helmholtz free-energy associated with cell adhesions and considering the contribution of the cell nucleus. The parameter differences between the two studies derive from these dissimilarities. The parameters for the cell adhesions were chosen by considering the assumption that $k_n \ll k_a$. A list of the material parameters is given in Table S1.

Orientation, aspect ratio, and area of a configuration

For each configuration (j), as in experiments, the cellular orientation and aspect ratio were obtained by fitting the outline of the cell with the best-fitting ellipse (in a least square sense), calculated using a least-square algorithm. The area of the cell was calculated by summing the areas of the deformed elements composing the cell in the deformed configuration (j).

Scaling of the computational results

We first performed computational simulations of myofibroblasts cultured on substrates with alternating adhesive and non-adhesive lines equal in widths, where the size of these widths is normalized to the diameter of the undeformed cell. In what follows, we describe the procedure to find the scaled version of these widths.

We first simulated the experiments for a large range of different line widths, spanning from lines that were small enough to enable the cell to spread on multiple lines, to lines wide enough to accommodate the cell on a single line. The computational results were presented with boxplots, with each boxplot corresponding to a different width of adhesive and non-adhesive lines. The value of the line widths is reported on the horizontal axis normalized to the diameter of the undeformed cell. Hereafter, we indicate this normalized value with l_w.

The simulations predicted that cells generally tend to co-align with adhesive lines, with the variability of alignment dependent on l_w. In particular, the alignment increased with l_w when $l_w \leq 0.3$ and decreased with l_w for $l_w \geq 0.7$. Interestingly, l_w values between 0.4 and 0.6 corresponded to a

change of trend, with alignment increasing from 0.3 to 0.5 and again decreasing for l_w from 0.5 to 0.7.

To compare the computational results with the experiments, the normalized line widths l_w have to be scaled with experimental measures. In our experiments, 20 μm seems a characteristic size for myofibroblasts, corresponding to the smallest line width where cells can spread on single lines (Figure 1 of the main text). Moreover, cells on these lines exhibited the maximum degree of alignment. Therefore, the normalized line widths were scaled by identifying the value of l_w that corresponds to 20 μm. For brevity, we indicate this value with l_w^{ref}. To determine l_w^{ref} we observe that, in the computational simulations, cells start spreading on single lines when $l_w \geq 0.6$ (Figure S4C). In addition, cells exhibit very high degrees of alignment for values of l_w in between 0.6 and 0.7. Consequently, given the similarities with the experimental results for 20 μm lines, we can assume that $0.6 < l_w^{ref} \leq 0.7$. To make the scaling more specific, another requirement for l_w^{ref} can be deduced by observing the experimental results of cells on large lines (such as 100 μm and 150 μm); cells on such lines still exhibit a relatively high parallel alignment. However, in the computational simulations, large values of l_w correspond to decreased alignment. Therefore, large experimental measures of line widths should correspond to relatively small l_w, which translates to the requirement that l_w^{ref} should be relatively small. Considering these two requirements, as a first approximation, we assumed that 20 μm corresponds to $l_w^{ref} = 0.625$ (such that $l_w = 1$ becomes equivalent to 32 μm). The computational simulations were then repeated for values of l_w corresponding with the other line widths analyzed in the experiments. The results of these computational simulations are reported in Figure 5 of the main text. In Figure **??**, morphologies of representative cells from the simulations and experiments are compared. In general, the cellular shapes obtained with the computational simulations are similar to the ones that were experimentally observed.

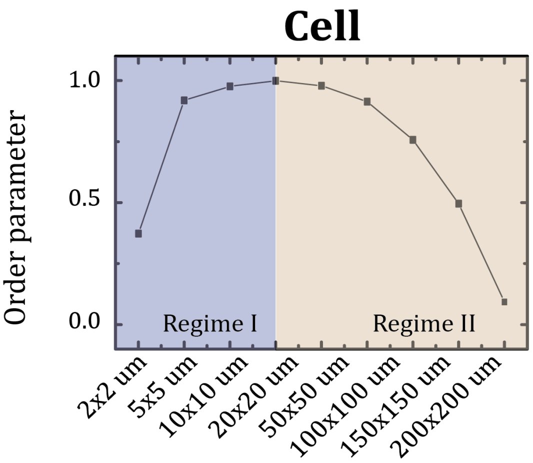

Figure S1. Measurements of the cell orientation order parameter Θ versus the line dimensions (width × spacing μm). The values of Θ range from 0–1, representing random alignment (Θ = 0) for 2 × 2 μm lines and for increasing width perfect alignment (Θ = 1). For $w > 20$ μm, Θ decreases for increasing line widths. This order parameter clearly shows the two different observed regimes in cellular alignment transitioning at a line width $w = 20$ μm. The results are expressed as the mean and are from three independent experiments. At least 60 cells were considered per condition.

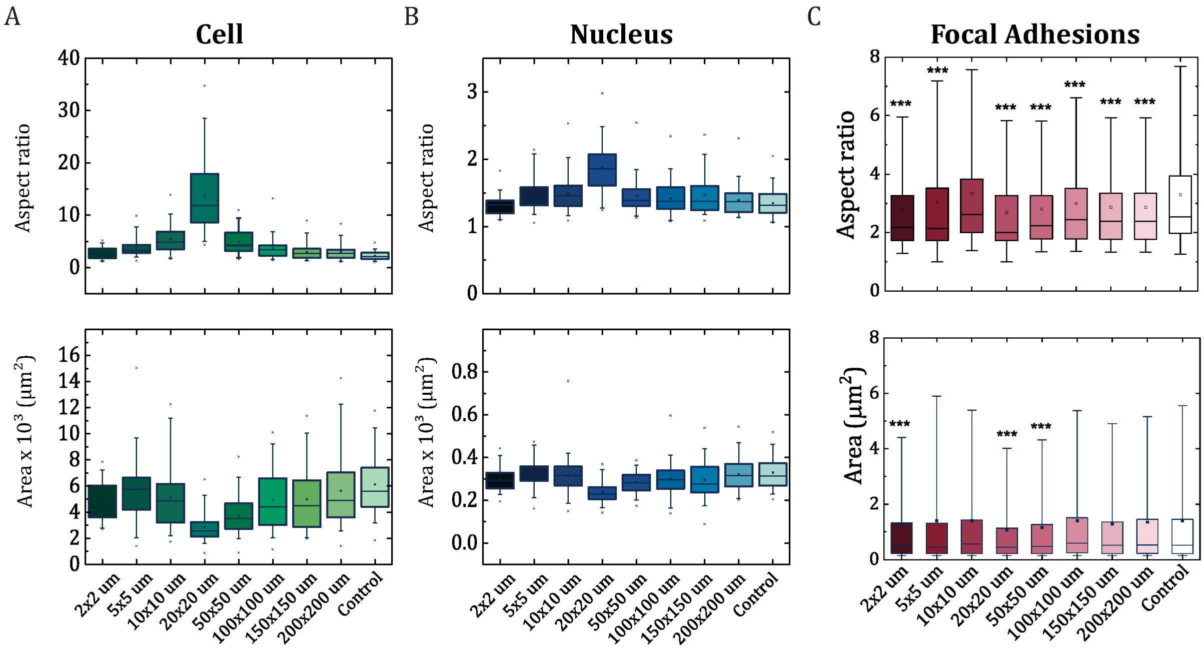

Figure S2. Distributions of area and aspect ratio for A) cells, B) nuclei, and C) FAs determined for myofibroblasts on fibronectin lines of various dimensions (width $\times$ spacing μm). The boxes of the boxplots represent the quartiles of the distributions with the whiskers indicating the outliers in the experiments and the 5th and 95th percentiles of the distributions. C) The data reported are results from three independent experiments, at least 60 (cells and nuclei) or 20 cells (FAs) were considered per condition. ***: $p < 0.001$ with respect to control.

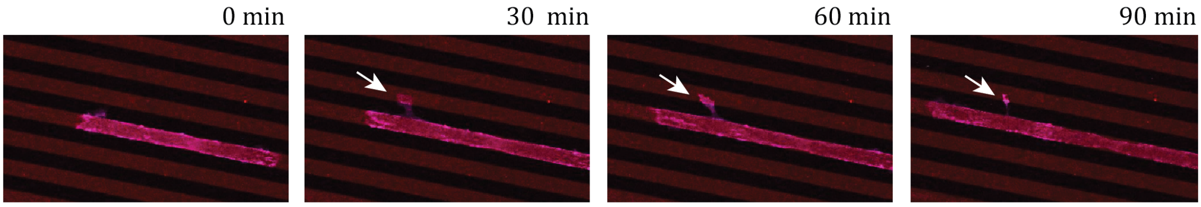

Figure S3. Snapshots from a movie of a spreading myofibroblast on 20 × 20 μm lines of fibronectin (red). The cell is stained for the FAs (magenta) and forms adhesions primarily on the periphery of the cell on the edges of the lines. The cell can be seem to form a protrusion perpendicular to the line bridging the non adhesive line as indicated by the white arrow. However, this protrusion is not stable and retracts.

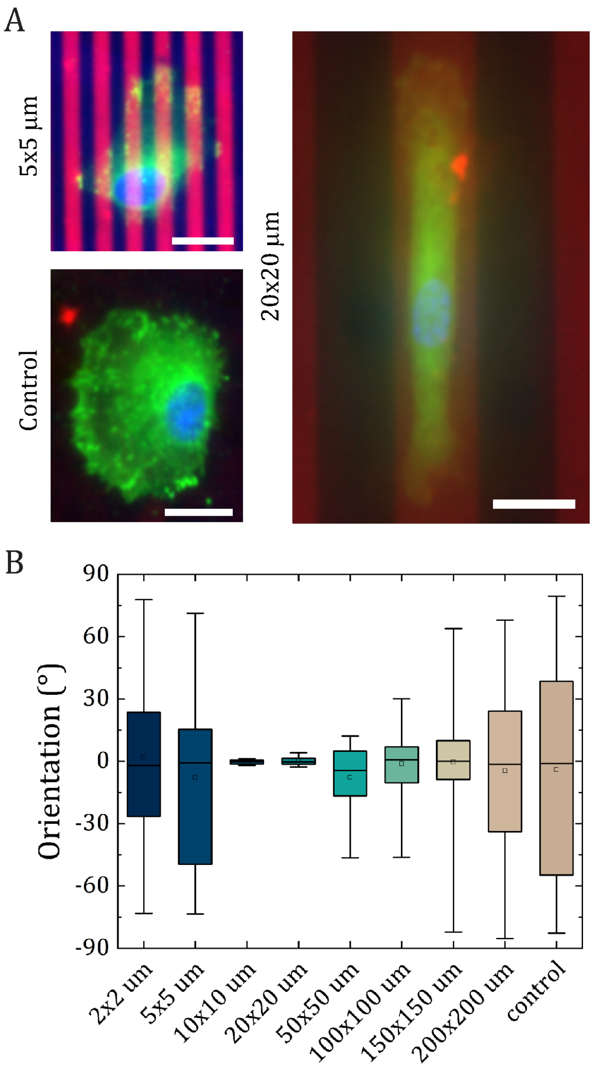

Figure S4. Substrate-pattern-guided contact guidance of cardiomyocyte progenitor cells (CMPCs). (A) Representative images of CMPCs on parallel fibronectin lines ($w \times s$ μm) stained for fibronectin (red), FAs (green), and nucleus (blue). Scale bar = 20 μm. (B) Box-and-whisker plot of the cell orientation distributions. At least 20 cells were analyzed per condition.

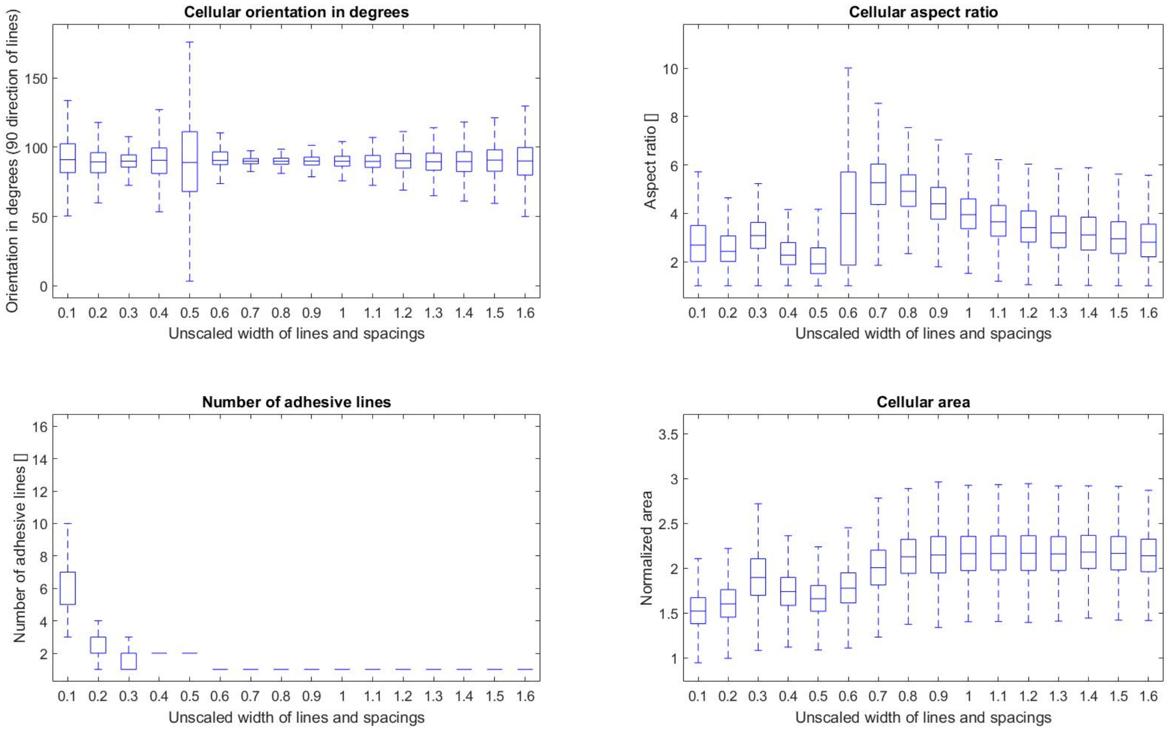

Figure S5. Overview of the results of the simulation of cells on substrates with alternating adhesive and non-adhesive lines of different line widths. The values of these widths are normalized with respect to the diameter of the undeformed cell. For simplicity, the outliers were not reported in the graphs.

Table S1. Parameter set for computational simulation of myofibroblasts on substrates with alternating adhesive and non-adhesive lines

Parameter	Value	Brief description
T	310 K	Thermodynamic temperature
μ	1.67 kPa	In-plane shear modulus of the cellular passive components
κ	35 kPa	In-plane bulk modulus of the cellular passive components
m	6	Material constant modulating the nonlinearity of the deviatoric elastic response of the cellular passive components
$\bar{k}$	1 GPa	Parameter modulating the elastic penalty as a result of a large reduction of cell area
J_c	0.6	Penalty parameter associated with a large reduction of cell area
σ_{max}	240 kPa	Maximum stress-fibre contractile stress
ρ_0	$3 \cdot 10^6$ μm^{-3}	Density of stress-fibre proteins
$\mathcal{F}_0$	0.032	Stress-fibre protein volume fraction
$\tilde{\varepsilon}^{ss}_{nom}$	0.35	Steady state stress-fibre functional unit strain
$\mu_{b0} - \mu_u$	$1kT$	Difference between bound and unbound stress-fibre potential
Ω	$10^{-7.1}$ μm^3	Volume of the reference number of functional units that are within a stress-fibre in the undeformed representative volume element
$\hat{\eta}_{max}$	1	Concentration of bound stress-fibre proteins
b_0/R_0	0.2	Ratio between the thickness and radius of an undeformed cell
$k_n/(\mu R_0)$	$1.061 \cdot 10^2$	Nondimensional representation of the stiffness of linear springs approximating focal adhesions on non-adhesive lines
k_a/k_n	10^2	Ratio between the stiffness of linear springs approximating focal adhesions on adhesive areas and the value for non-adhesive areas

Supplemental References

[1] Shishvan SS, Vigliotti A, Deshpande VS. The homeostatic ensemble for cells. Biomechanics and Modeling in Mechanobiology. 2018;17(6):1631–1662.

[2] Vigliotti A, Ronan W, Baaijens FPT, Deshpande VS. A thermodynamically motivated model for stress-fiber reorganization. Biomechanics and Modeling in Mechanobiology. 2016;15(4):761–789.

[3] Ogden RW. Large Deformation Isotropic Elasticity: On the Correlation of Theory and Experiment for Compressible Rubberlike Solids. Proceedings of the Royal Society A: Mathematical, Physical and Engineering Sciences. 1972;328(1575):567–583.

[4] Metropolis N, Rosenbluth AW, Rosenbluth MN, Teller AH, Teller E. Equation of State Calculations by Fast Computing Machines. The Journal of Chemical Physics. 1953;21(6):1087–1092.

[5] Piegl L, Tiller W. Symbolic operators for NURBS. CAD Computer Aided Design. 1997;29(5):361–368.

[6] Buskermolen ABC, Suresh H, Shishvan S, Vigliotti A, DeSimone A, Kurniawan NA, et al. Entropic forces drive cellular contact guidance. Biophysical Journal. 2019;116(10):1994–2008.
